# 5-Deazaflavin derivatives as inhibitors of p53 ubiquitination by HDM2^☆^

**DOI:** 10.1016/j.bmc.2013.09.038

**Published:** 2013-11-15

**Authors:** Michael P. Dickens, Patricia Roxburgh, Andreas Hock, Mokdad Mezna, Barrie Kellam, Karen H. Vousden, Peter M. Fischer

**Affiliations:** a School of Pharmacy & Centre for Biomolecular Sciences, University of Nottingham, University Park, Nottingham NG7 2RD, UK; b The Beatson Institute for Cancer Research, Garscube Estate, Switchback Road, Glasgow G61 1BD, UK

**Keywords:** Cancer, HDM2–p53, Ubiquitination inhibitors, Ubiquitin E3 ligase, Deazaflavin

## Abstract

Based on previous reports of certain 5-deazaflavin derivatives being capable of activating the tumour suppressor p53 in cancer cells through inhibition of the p53-specific ubiquitin E3 ligase HDM2, we have conducted an structure–activity relationship (SAR) analysis through systematic modification of the 5-deazaflavin template. This analysis shows that HDM2-inhibitory activity depends on a combination of factors. The most active compounds (e.g., **15**) contain a trifluoromethyl or chloro substituent at the deazaflavin C9 position and this activity depends to a large extent on the presence of at least one additional halogen or methyl substituent of the phenyl group at N10. Our SAR results, in combination with the HDM2 RING domain receptor recognition model we present, form the basis for the design of drug-like and potent activators of p53 for potential cancer therapy.

## Graphical abstract

**Figure:**
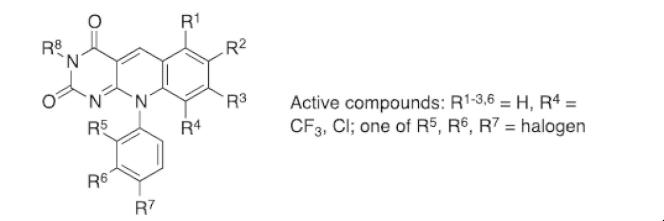

## 1 Introduction

The tumour suppressor p53 is central to the protection of mammalian cells against genetic damage and p53 is dysfunctional in a large proportion of cancer cells.^1^ The oncogene HDM2 and p53 are linked in a negative feed-back loop in which p53 activates HDM2, the latter acting as a p53-specific ubiquitin E3 ligase and thus promoting degradation of p53 protein through the ubiquitin–proteasome system.^2^ Tumours that retain wild-type p53 frequently show aberrations in p53 regulation, most commonly through overexpression of the p53 negative regulator HDM2.^3^ A strategy for the reactivation of the pro-apoptotic p53 activities in such tumours is therefore to interrupt the p53–HDM2 feed-back loop, either by blocking the protein–protein interaction between the p53 N-terminal domain and HDM2, or by inhibiting the E3 ligase activity of HDM2.^4^

**Figure 1:**
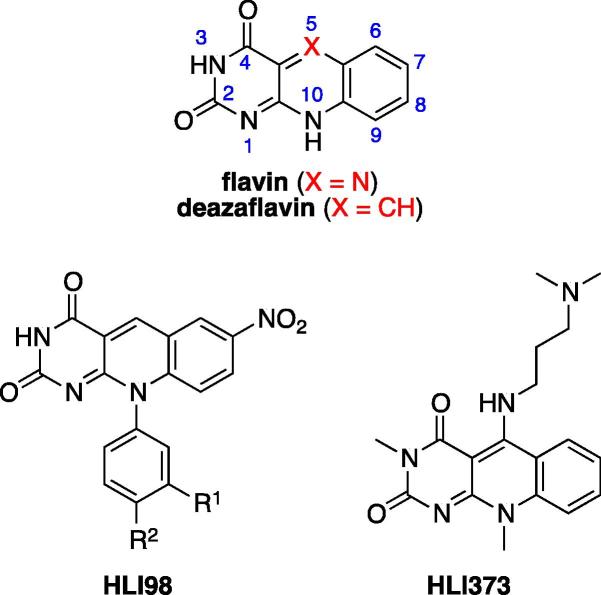
The chemical structures of flavin and 5-deazaflavin (numbering system indicated in blue), as well as derivatives of 5-deazaflavin known as HDM2 ligase inhibitors (HLI).^5^, ^6^, ^8^

We have previously reported on a family of 7-nitro-5-deaza-flavin compounds, which were discovered in a screen for inhibitors of HDM2 E3 activity.^5^ This group of compounds was named the HDM2 ligase inhibitor 98 class (HLI98; Fig. 1). A compound known as HLI373 (Fig. 1), whose structure differs significantly from active HLI98 compounds, has also been reported to inhibit the E3 ubiquitin ligase activity of HDM2 and thereby selectively to kill cancer cells in a p53-dependent manner.^6^ Whereas 7-nitro-5-deazaflavins inhibit both HDM2-mediated p53 ubiquitination and auto-ubiquitination, other reported compounds that target the ubiquitin E3 ligase activity of HDM2 apparently are selective for p53 ubiquitination.^7^

Using surface plasmon resonance spectroscopy we found that active, but not inactive 5-deazaflavin analogues, bound to the HDM2 RING domain. In cellular assays, active 5-deazaflavin compounds inhibited p53 ubiquitination, stabilised p53, and induced the expression of p53 targets.^9^

The flavin and 5-deazaflavin (also known as 5-deaza-isoalloxazine or pyrimido[4,5-*b*]quinoline-2,4(3*H*,10*H*)-dione (Fig. 1)) substructures are found in a number of naturally occurring redox cofactors.^10^, ^11^ Various synthetic 5-deazaflavin derivatives have been reported to possess antibacterial, antiparasitic, and anticancer pharmacological activities, although the molecular targets for these activities remain unknown in most cases.^12^, ^13^

Because of the high reduction potential of nitro-5-deazaflavins, they easily undergo biological one-electron reduction to generate nitro anion radicals, which, when present in flat heteroaromatic systems that can interact with DNA,^14^ are known to induce cytotoxicity through DNA damage.^15^, ^16^, ^17^ It has been shown that certain nitro-5-deazaflavins have antitumour activities^18^ and that 6- and 8-nitro derivatives (containing long-chain alkyl groups at N10 instead of the phenyl group present in HLI98 compounds) generate stable one-electron reduction products with selective cytotoxicity against hypoxic cells.^19^ Furthermore, direct interaction between a 5-deazaflavin–oligonucleotide conjugate and DNA has been demonstrated.^20^, ^21^

With regards to the HLI98 class of compounds as leads for pharmacological p53 reactivation, we were interested in abolishing the possibility of their promiscuous cytotoxic activity through bioreductive activation and DNA damage. Preliminary SAR studies indicated that the cellular activity of 10-aryl-5-deazaflavins was in fact not due to the C7-nitro function, since both cell-inactive 7-nitro derivatives and cell-active analogues devoid of the 7-nitro group were observed.^8^ Here we report on the synthesis and structure–activity relationships (SARs) with respect to HDM2 E3 ligase inhibition and cellular activity of an extensive set of 5-deazaflavin derivatives.

## 2 Chemistry

**Scheme 1:**
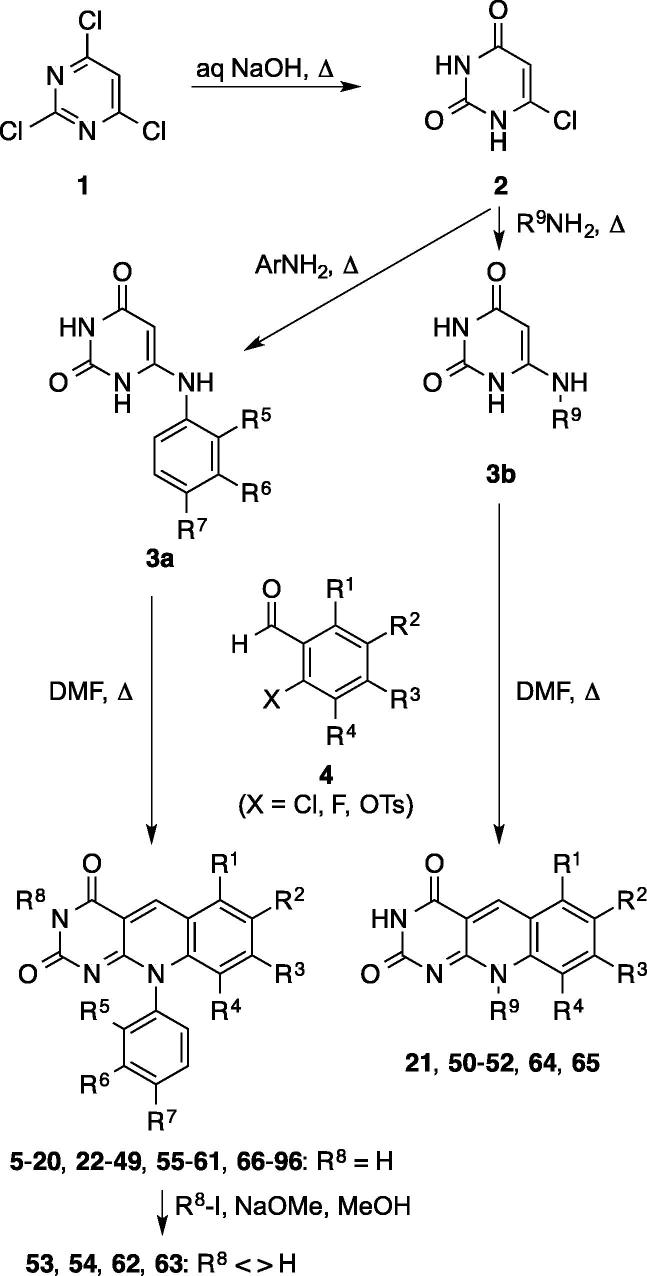
Synthesis of 5-deazaflavin derivatives **5**–**96**. For definition of substituents R^1–9^ refer to Table 1.

The 5-deazaflavins **5**–**96** (Table 1 & Table S1) were prepared as shown in Scheme 1. 6-Chlorouracil **2**, prepared by hydrolysis of 2,4,6-trichloropyrimidine **1**,^22^ was fused with the appropriate anilines or other primary amines to afford the 6-anilinouracils **3a** and 6-(ar)alkyluracils **3b** in moderate to high yield.^23^ These were then submitted to cyclocondensation with 2-halobenzaldehydes **4**.^8^, ^24^, ^25^ The latter were chosen depending on commercial availability. The 6-chlorodeazaflavins could be prepared unambiguously using 2-chloro-6-fluorobenzaldehyde, due to the enhanced reactivity of the *ortho*-fluoro compared to the *ortho*-chloro group in the cyclocondensation reaction.

**Table 1:** Inhibition of in vitro ubiquitination of p53 by HDM2 by substituted 10-aryl (**I**) and 10-alkyldeazaflavins (**II**)^a^

Compd										Prescreen	IC_50_ (μM)
	R^1^	R^2^	R^3^	R^4^	R^5^	R^6^	R^7^	R^8^	R^9^		
**5 (I)**	H	H	H	NO_2_	F	H	H	H	—	Active	>100
**6 (I)**	H	NO_2_	H	H	H	H	Cl	H	—	Active	>75
**7 (I)**	H	H	H	NO_2_	H	H	Cl	H	—	Active	>50
**8 (I)**	H	H	H	CF_3_	H	H	H	H	—	Active	17.5^b^
**9 (I)**	H	H	CF_3_	H	F	H	H	H	—	Active	>100
**10 (I)**	H	H	H	CF_3_	F	H	H	H	—	Active	19.6^b^
**11 (I)**	CF_3_	H	H	H	H	H	Cl	H	—	Active	>100
**12 (I)**	H	CF_3_	H	H	H	H	Cl	H	—	Active	>100
**13 (I)**	H	H	CF_3_	H	H	H	Cl	H	—	Active	>100
**14 (I)**	H	H	H	CF_3_	H	H	Cl	H	—	Active	11.9^b^
**15 (I)**	H	H	H	CF_3_	H	Cl	H	H	—	Active	1.5^b^
**16 (I)**	H	H	H	CF_3_	H	Cl	Cl	H	—	Active	10.7^b^
**17 (I)**	H	H	H	CF_3_	H	F	H	H	—	Active	12.3^b^
**18 (I)**	H	H	H	CF_3_	H	H	F	H	—	Active	13.1^b^
**19 (I)**	H	H	H	CF_3_	H	Me	H	H	—	Active	8.4^b^
**20 (I)**	H	H	H	CF_3_	H	H	Me	H	—	Active	32^b^
**21 (II)**	H	H	H	CF_3_	—	—	—	H	Bn	Active	>100
**22 (I)**	H	H	H	Cl	H	H	H	H	—	Active	>100
**23 (I)**	H	Cl	H	H	F	H	H	H	—	Active	>100
**24 (I)**	Cl	H	H	H	H	H	Cl	H	—	Active	>100
**25 (I)**	H	H	H	Cl	H	H	Cl	H	—	Active	15.0^b^
**26 (I)**	H	H	H	Cl	H	Cl	H	H	—	Active	>100
**27 (I)**	H	H	H	Cl	H	F	H	H	—	Active	>100
**28 (I)**	H	H	Me	H	H	H	Cl	H	—	Active	>100^b^
**29 (I)**	H	H	H	Br	H	H	H	H	—	Active	>100
**30 (I)**	H	H	H	Br	H	H	Cl	H	—	Active	49.5^b^

a For explanation of prescreen refer to text; for summary of inactive compounds refer to Table S1.

b IC_50_ values correspond to those reported previously^9^ for compounds **8**, **10**, **14**–**20**, **25**, **28**, and **30**; corresponding to MPD compounds **19**, **20**, **37**, **131**, **162**, **134**, **137**, **140**, **146**, **32**, **39**, and **159**, respectively, in Ref. 9.

Where no 2-halobenzaldehyde but 2-hydroxybenzaldehyde starting materials were available, 2-tosylbenzaldehydes (**4**, X = OTs) were prepared and used successfully in the deazaflavin cyclocondensation reaction. Other benzaldehydes were prepared by literature procedures. Thus 2-fluoro-6-nitrobenzaldehyde was prepared from 2-fluoro-6-nitrotoluene by the nitrone method,^26^ whereas 2-chloro-4-nitrobenzaldehyde^27^ was prepared from methyl 2-chlorobenzoate by (*i*Bu_2_AlH)_2_ reduction.^28^ 3-Formyl-2-hydroxybenzonitrile was obtained by Reimer-Tiemann *ortho*-formylation of 2-hydroxybenzonitrile.^29^

## 3 Results and discussion

**Figure 2:**
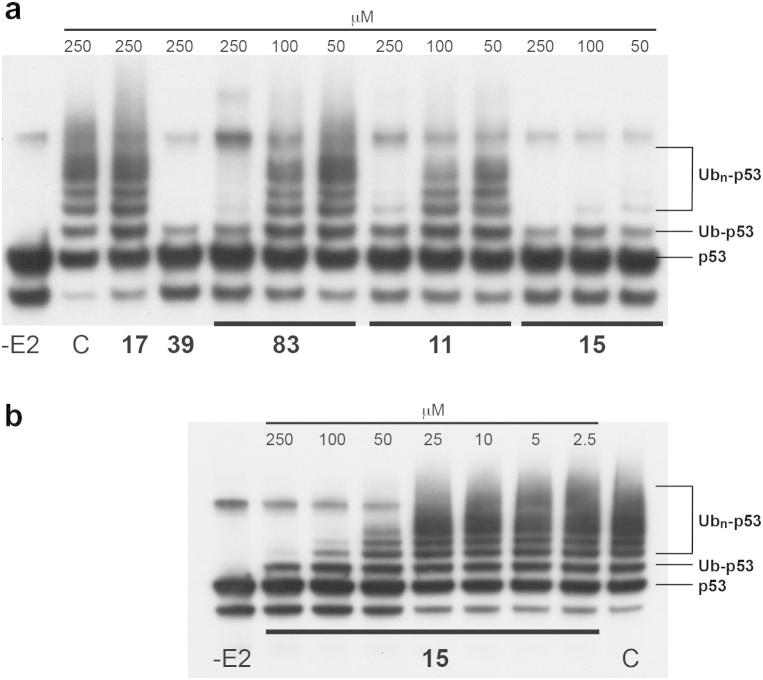
Example of p53 ubiquitination assay for selected compounds (refer Table 1). Western blots with anti-p53 antibody of SDS–PAGE separation of reaction mixtures after incubation of immobilised HDM2 with test compounds or diluent only (C) and p53, ubiquitin, and ubiquitin ligases (-E2 indicates reactions omitting E2 ligase). Prescreen at 50–250 μM for compounds with different activity levels (a) and titration of compound **15** (b). The positions of p53, mono-ubiquitinated (Ub-53), and poly-ubiquitinated p53 (Ub_n_-p53) are indicated.

Test compounds were assayed for inhibition of p53 ubiquitination^9^, ^30^ by incubation with GST-tagged HDM2, immobilised on glutathione-Sepharose, p53, ubiquitin, as well as E1 and E2 (UbcH5B) ligases, in an assay buffer containing ATP. The reaction products were then resolved by SDS–PAGE and p53 ubiquitination was quantitated by Western blotting using an anti-p53 antibody (Fig. 2).^31^ Test compounds that were active as inhibitors at concentrations below 100 μM in this assay (labelled ‘prescreen’ in Table 1) were then subjected to determination of half-maximal inhibition concentration (IC_50_) of p53 ubiquitination using essentially the same assay methodology, except that a fluorescently labelled form of ubiquitin was used. After completion of the ubiquitination reaction, excess fluorescent ubiquitin was removed from the immobilised p53–HDM2 complex by centrifugation, and incorporation of ubiquitin was measured by fluorescence spectroscopy.

Based on the original HLI98 7-nitro-5-deazaflavins, we examined the role of the nitro substituent in the benzene ring of the tricyclic deazaflavin system (Table 1 & Table S1). As expected,^5^, ^8^ we confirmed the activity of the 7-nitro derivative containing a *para*-chloro-substituted 10-phenyl group (**6**). However, we also found that introduction of a nitro group at C9 resulted in moderately active compounds in the context of either a 10-*para*-chlorophenyl (**7**) or 10-*ortho*-fluorophenyl group (**5**). Substituents on the 10-phenyl group appeared to be important, since the 7-nitro derivative containing an unsubstituted 10-phenyl group (**32**; refer Table S1 for summary of inactive compounds) was inactive.

As mentioned previously, we were interested in removing the nitro group altogether and therefore turned our attention to replacing this group with alternative substituents. The trifluoromethyl group has similar steric and electronic properties to the nitro group and we examined a number of 5-deazaflavin derivatives incorporating this function. We observed that in the absence of substituents on the 10-phenyl group, introduction of a trifluoromethyl group in the benzene ring of the 5-deazaflavin tricyclic system only afforded an active compound in the case of the 9-trifluoromethyl derivative **8**.

In the case of analogues with the 6-, 7-, 8-, or 9-trifluoro-methyl groups in combination with either the 10-*ortho*-fluorophenyl or 10-*para*-chlorophenyl substitutions, similar results were observed insofar as only the 9-trifluoromethyl derivatives **8** and **10** showed significant biological activity, whereas the compounds with the trifluoromethyl group at other positions were less active (**9**, **11**–**13**). The 9-trifluoromethyl compounds **8** and **10** were observed to have significantly enhanced potency compared to the corresponding 9-nitro compounds **5** and **7**. Further elaboration of the halogen substituents on the 10-phenyl group in the context of the 9-trifluoromethyl-5-deazaflavin system revealed that chloro and fluoro groups were in fact also tolerated in the *meta*- and *para*-positions (**14**–**18**), but not in the *ortho*-position in the case of the larger chloro group. The most potent compound in this series was the *meta*-chloro derivative **15**.

A similar situation was revealed when the 10-phenyl halogen substituents in 9-trifluoromethyl-5-deazaflavins were replaced with methyl groups. Of the three 10-toluyl derivatives assayed, the *meta*-isomer **19** was most active, followed by the less potent *para*-isomer **20**. As in the nitro series, modification of the active 9-trifluoro-methyl-10-aryl-5-deazaflavin core by alkylation at N3 (**62** and **63**; Table S1) abolished activity, and the only replacement of the 10-aryl group that was tolerated was with a benzyl group (**21**).

We next examined the effect of groups other than the strongly electron-withdrawing nitro and trifluoromethyl functions on the biological activity of 5-deazaflavin compounds. The weakly electron-withdrawing chloro substituent in the contexts of an unsubstituted, *ortho*-fluoro- or *para*-chloro-substituted 10-phenyl group resulted in poorly active (**22**–**24**) or inactive derivatives, with the exception of the combination of chloro substituents at both C9 and at the *para*-position of the 10-phenyl group, which afforded a compound (**25**) with reasonable activity. This situation is similar to the SARs in the 9-trifluoro-methyl series; however, here alternative halogen substitutions in the 10-phenyl group were less well tolerated (**26** and **27**).

Replacement of the electron-withdrawing nitro, trifluoromethyl, or chloro substituents in the benzene ring of the 5-deazaflavin ring system with the electron-releasing and sterically undemanding methyl group resulted in inactive compounds when the 10-phenyl group was unsubstituted. Unlike in the case of the 9-trifluoromethyl series, in the otherwise active context of the *para*-chloro- or *ortho*-fluoro-substituted 10-phenyl group, this resulted in a clear loss or decline in biological activity in all cases, with only compound **28** retaining marginal activity.

In the case of the more strongly electron-releasing hydroxyl group at positions C9 of the deazaflavin system, no biological activity was obtained, regardless of 10-phenyl substitution. However, 9-bromo (**29** and **30**) and 9-fluoro derivatives were also comparatively inactive, and the same was true for both derivatives with the strongly electron-withdrawing cyano group at C9 (**95** and **96**; Table S1).

Overall these results suggest that the SARs for inhibition of HDM2 E3 ubiquitin ligase activity in substituted 5-deazaflavin derivatives depend on a combination of factors. The most active compounds contain a trifluoromethyl or chloro substituent at C9 and this activity depends to a large extent on the presence of at least one additional halogen or methyl substituent of the phenyl group at N10. Although we have not yet tackled modification of the heterocyclic rings in the 5-deazaflavin system extensively, SARs in that region appear to be quite restrictive and neither replacement of the (substituted) 10-phenyl nor the N3-H appear to be tolerated. Selectivity of the 5-deazaflavins described here for the HDM2 E3 ubiquitin ligase has been demonstrated previously^9^ using similar assay systems for inhibition of auto-ubiquitination of either HDM2 or the related RING E3 ubiquitin ligase CBL.^32^

Some of the compound structures described here coincide with those reported earlier by Wilson et al.^8^ and in general there is agreement between the in vitro activity described here and the activity of the relevant compounds in cells, as measured by Wilson et al.^8^ However, two 6-chloro-5-deazaflavin derivatives described by Wilson et al. (**24** and **69**, corresponding to compounds **14** and **16**, respectively, in Wilson et al.^8^) were found to activate p53 in cells, while our data indicate that these compounds cannot directly inhibit HDM2. These results suggest that these compounds induce p53 through an indirect mechanism in cells, and that the cell activity is an off-target effect. In the in vitro assays described here, we show that the 9-trifluoromethyl-5-deazaflavins are the most active compounds. Only two of these were reported by Wilson et al.^8^ (**10** and **18**, corresponding to compounds **27** and **28**, respectively, in Wilson et al.^8^), and both were found to be inactive in cells. To resolve this apparent paradox, we compared our compound analyses with those reported by Wilson et al.^8^ We found that spectroscopic data matched well in some cases but detected discrepancies for those compounds where the pharmacological data is at variance (i.e. **10** and **18**, corresponding to compounds **27** and **28**, respectively, in Wilson et al.^8^). In these cases there is poor correlation between the ^13^C NMR spectra and in each case the ^1^H NMR description in Wilson et al.^8^ lacks the characteristic singlet peak for the C5-H resonance in the *δ* 9.0–9.3 region, which we observe in the spectra of all our compounds. During subsequent biological testing of the compounds synthesised here, we noted a cumulative loss of activity for several of the active compounds, suggesting that they may show instability. We therefore believe that this instability of these compounds may account for the apparent discrepancies in biological activity and spectroscopic data.

Both 5-deazaflavins (X = CH, Fig. 1) and flavins (X = N) contain a redox system and undergo facile hydride transfer reduction and the reduced 1,5-dihydro species are oxidised back readily.^11^, ^33^ In the case of 5-deazaflavins, which contain an electrophilic CH function at C5, nucleophilic attack at that position is comparatively facile. Electron withdrawing substituents such as nitro, trifluoromethyl, etc. in the tricyclic system enhance the reactivity of the redox system^34^ and it would therefore be important to develop potent analogues that have neutral or electron-donating substituents in the benzo ring, since these should be more stable. Apart from extending the SARs of the 5-deazaflavins through synthesis and testing of more analogues, the most important aspect would be to modulate the electrophilic reactivity of the system. This inherent property is undesirable because reactivity, especially as far as interference with physiological redox systems is concerned, might lead to poor bioavailability and potentially toxicity. It should be noted, however, that even flavins, which are more reactive than 5-deazaflavins, appear to have drug-like properties potential, based on the fact that certain flavin derivatives with antimalarial activity and close structural similarity to the 5-deazaflavins of the current report, have been shown to possess in vivo activity by both the parenteral and oral administration routes in a malaria mouse model.^13^, ^35^

**Figure 3:**
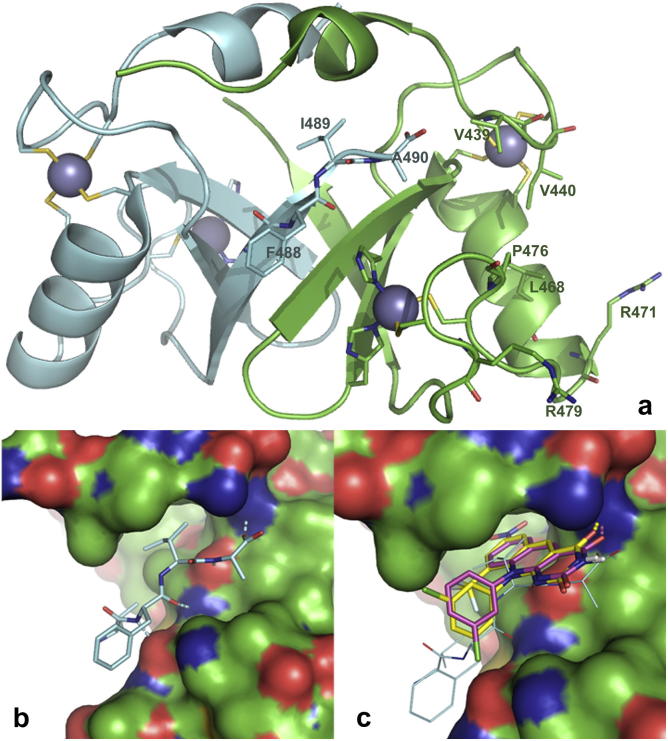
HDM2–HDMX RING domain heterodimer complex (constructed from PDB entry 2VJF). (a) Secondary structure cartoon with HDM2 in green and HDMX in cyan. The two zinc ions in each RING domain are shown as spheres with the coordinating residue side chains as sticks. A composite of one face of HDM2, together with the C-terminus of HDMX (key residues shown with side chains and labelled), forms the likely interaction site with the E2 ubiquitin conjugating enzyme. (b) Interaction of the C-terminus of HDMX (cyan) with HDM2 (green CPK surface). (c) Predominant docked poses of compounds HLI98C (magenta) and **15** (yellow).

Despite the availability of structural information on the HDM2 RING domain,^36^, ^37^, ^38^, ^39^ its E3 ligase catalytic mechanism remains unclear since neither the nucleotide-binding site nor the active site have been delineated. The structural basis of the inhibition of the HDM2 E3 ligase activity by our compounds thus also remains uncertain. It has been shown that the extreme C-terminal tripeptide of HDM2 is critical for the HDM2 E3 ligase activity^40^, ^41^ and a structural solution of an HDM2–HDMX RING domain heterodimer^38^ shows that this tripeptide binds a groove of the partner protein, thus forming a composite binding site for the E2-ubiquitin complex (Fig. 3a & b). Since this interaction is apparently required for HDM2 E3 ligase activity both in *trans* and in *cis*,^40^ it is possible that nonselective inhibitors, i.e. compounds such as our 5-deazaflavins that do not distinguish between inhibition of HDM2 auto-ubiquitination and ubiquitination of p53,^9^ target this E2-binding site.

We therefore extracted the coordinates of the HDM2 RING domain^38^ and docked bioactive 5-deazaflavins to this receptor by defining the entire domain as the receptor site. We found that all plausible and highly scoring poses put the ligands exactly where the tripeptide tail in the above complex structure is (Fig. 3c). In fact it can be observed that the 5-deazaflavins appear to mimic the terminal residues of the peptide, with the substituted fused phenyl ring of the 5-deazaflavin system occupying the position of the Ile residue, the pyrimidinedione ring in the position of the Ala residue, and the 10-aryl group projecting towards the Phe residue. The main determinant of activity, i.e. a 9-trifluoromethyl group, may be due to favourable interactions of that group with a pocket delineated by HDM2 residues Leu^430^ and Leu^458^. Substitution at C9 of the 5-deazaflavin system also imposes rotational limitations on the 10-aryl group, thus locking this group into conformations which positions its halogen substituents, which are important for activity, for interactions with either His^457^ or Leu^430^–Pro^431^. While these finding are suggestive, it remains to be shown experimentally that HDM2-inhibitory 5-deazaflavins compete directly with the C-terminal peptide.

## 4 Conclusion

Following our recent elucidation of the fact that bioactive 5-deazaflavins stabilize and activate p53 by binding directly to the HDM2 RING domain,^9^ here we have presented a comprehensive SAR analysis of 5-deazaflavin as inhibitors of p53 ubiquitination by the p53-specific ubiquitin E3 ligase HDM2. Together with the structural model of the mode of interaction of 5-deazaflavins with HDM2, it should now be possible to design and develop drug-like and potent inhibitors of HDM2 E3 ligase activity for potential therapeutic application in oncology.

## 5 Experimental section

### 5.1 General

Silica gel (Merck Kieselgel 60, 230–400) flash column chromatography was carried out as described^42^ or using Biotage Argonaut Flash Master II and Biotage Flash Master Personal instruments. Melting points were recorded using Gallenkamp and Bibby Stuart Scientific (SMP3) apparatus and are uncorrected. NMR spectra were recorded on a Bruker-AV 400 instrument at 400.1 MHz (^1^H) or 75 MHz (^13^C) with compound solutions in DMSO-*d*_6_ (unless otherwise indicated). Chemical shifts (*δ*) are reported in ppm with reference to the chemical shift of residual solvent or the internal standard SiMe_4_. Mass spectra (TOF-ES) were recorded using a Waters 2795 Separation Module and Micromass LCT platform. Infrared spectra were recorded using Avatar 360 Nicolet FT-IR and Nicolet IR200 FT-IR spectrophotometers. A Waters 2525 Binary Gradient Module was used for analytical RP-HPLC with a Waters 2487 Dual Wavelength Absorbance UV Detector. Analytical RP-HPLC was used to confirm purity for all compounds by chromatogram integration at 254 nm, using two systems. System A: Phenomenex Onyx monolithic reversed phase C_18_ column (100 × 3.0 mm) with a flow rate of 3 mL/min and using isocratic elution with 7:3 H_2_O–MeCN. System B: Phenomenex Kromasil reversed phase C_18_ column (250 × 4.6 mm) with a flow rate of 1 mL/min and using isocratic elution with 1:1 H_2_O–MeOH. Biological assays were carried out as described.^9^

### 5.2 General method for the preparation of 6-anilinouracils (3a)

To 6-chlorouracil (**2**, 1 equiv) was added the appropriate aniline (6 equiv) and the mixture was heated at 180–200 °C for 1.5–3 h. It was then cooled and the residue was triturated with Et_2_O. Precipitated product was filtered and washed on a sinter with Et_2_O and H_2_O. After drying, the crude product was crystallised from EtOH.

In this manner pure 6-anilinouracils **3a** were obtained, whose analytical details were in accord with the literature. E.g. 6-[(3-methylphenyl)amino]-1,2,3,4-tetrahydropyrimidine-2,4-dione (**3a**, R^5,7^ = H, R^6^ = Me) in 71% yield as an off-white solid with mp 303–304 °C (lit.^43^ 302–303 °C); IR (KBr): 3211 (NH), 3053 (NH), 1734 (C=O), 1625 (C=C) cm^−1^; ^1^H NMR: *δ* 2.30 (3H, s, Me), 4.68 (1H, s, C5-H), 6.95–7.02 (3H, m, Ph-H), 7.26 (1H, t, *J* = 7.5 Hz, Ph 5-H), 8.16 (1H, s, NH), 10.10 (1H, s, N1-H), 10.43 (1H, s, N3-H); ^13^C NMR: *δ* 17.78 (CH_3_), 75.21 (CH), 126.66 (CH), 127.07 (CH), 127.32 (CH), 131.45 (CH), 134.21 (Cq), 136.12 (Cq), 151.29 (Cq), 153.54 (Cq), 164.70 (Cq); HRMS (ESI^+^): calcd for C_11_H_12_N_3_O_2_ [M+H]^+^ 218.0930, found 218.0923.

### 5.3 General method for the preparation of 2-tosylbenzalde-hydes (4, X = OTs)

A mixture of the appropriate salicylaldehyde (1 equiv) and Na_2_CO_3_ (4 equiv) in Me_2_CO (5 mL/mmol salicylaldehyde) was stirred for 30 min under N_2_, when tosyl chloride (2 equiv) in Me_2_CO (2 mL/mmol) was added. The solution was heated under reflux for 5 h, cooled, and concentrated under reduced pressure. The residue was purified by flash chromatography (2:1 hexane–Et_2_O) to afford the product.

### 5.4 2-Formyl-5-methylphenyl 4-methylbenzene-1-sulfonate (4, R^1,2,4^ = H, R^3^ = Me, X = OTs)

Prepared using general method 5.3 from 2-hydroxy-4-methylbenzaldehyde (0.50 g, 3.7 mmol). White solid (0.63 g, 59%). Mp 93–94 °C; ^1^H NMR: *δ* 2.36 (3H, s, Me), 2.42 (3H, s, Me), 7.06 (1H, s, C3-H), 7.33 (1H, d, *J* = 7.8 Hz, C5-H), 7.48 (2H, d, *J* = 8.2 Hz, Ph 3-H), 7.70 (1H, d, *J* = 7.8 Hz, C6-H), 7.76 (2H, d, *J* = 8.2 Hz, Ph 2-H), 9.83 (1H, s, CHO); ^13^C NMR: *δ* 21.65 (CH_3_), 21.66 (CH_3_), 124.44 (CH), 126.96 (Cq), 128.86 (CH), 128.96 (CH), 129.31 (CH), 130.85 (CH), 130.90 (Cq), 146.95 (Cq), 147.74 (Cq), 150.51 (Cq), 187.45 (CH).

### 5.5 2-Bromo-6-formylphenyl 4-methylbenzene-1-sulfonate (4, R^1–3^ = H, R^4^ = Br, X = OTs)

Prepared using general method 5.3 from 3-bromo-2-hydroxy-benzaldehyde (0.57 g, 2.8 mmol). White solid (0.82 g, 82%). Mp 79–91 °C; ^1^H NMR: *δ* 2.46 (3H, s, Me), 7.44–7.58 (3H, m), 7.77–7.88 (3H, m), 8.05 (1H, d, *J* = 7.3 Hz, C4-H), 9.86 (1H, s, CHO); ^13^C NMR: *δ* 21.74 (CH_3_), 118.58 (Cq), 128.40 (CH), 129.17 (CH), 129.94 (CH), 131.02 (CH), 131.28 (Cq), 132.40 (Cq), 139.98 (CH), 147.44 (Cq), 147.65 (Cq), 187.53 (CH).

### 5.6 2-Cyano-6-formylphenyl 4-methylbenzene-1-sulfonate (4, R^1–3^ = H, R^4^ = CN, X = OTs)

Prepared using general method 5.3 from 3-formyl-2-hydroxybenzonitrile (0.34 g, 1.7 mmol).^29^ White solid (0.48 g, 94%). Mp 84–86 °C; ^1^H NMR: *δ* 2.46 (3H, s, Me), 7.55 (2H, d, *J* = 8.5 Hz, Ph 3-H), 7.75 (1H, t, *J* = 7.6 Hz, C5-H), 7.82 (2H, d, *J* = 8.5 Hz, Ph 2-H), 8.15 (1H, dd, *J* = 1.8, 7.6 Hz, C6-H), 8.28 (1H, dd, *J* = 1.8, 7.6 Hz, C4-H), 9.84 (1H, s, CHO); ^13^C NMR: *δ* 21.76 (CH_3_), 109.73 (Cq), 114.46 (Cq), 129.27 (CH), 129.55 (CH), 129.95 (Cq), 131.22 (Cq), 131.25 (CH), 133.78 (CH), 140.01 (CH), 148.06 (Cq), 150.68 (Cq), 186.55 (CH).

### 5.7 General method for the preparation of 10-aryl-5-deaza-flavins using 2-halobenzaldehydes or 2-tosylbenzaldehydes

A mixture of a 6-(arylamino)pyrimidine-2,4(1*H*,3*H*)-dione **3a** (1 equiv) and a 2-halo- or 2-tosyl-benzaldehyde **4** (X = F, Cl, or OTs; 1.2 equiv) in DMF (10 mL/mmol **3a**) was heated under reflux for 4 h. After cooling, the solution was evaporated under reduced pressure and the residue was purified by flash chromatography (5:95 MeOH–CH_2_Cl_2_) to afford the product.

### 5.8 10-(2-Fluorophenyl)-9-nitro-2H,3H,4H,10H-pyrimido[4, 5-b]quinoline-2,4-dione (5)

Prepared using general method 5.7 from **3a** (R^5^ = F, R^6,7^ = H; 62 mg, 0.28 mmol) and **4** (R^1–3^ = H, R^4^ = NO_2_, X = OTs; 108 mg, 0.34 mmol).^44^, ^45^ Yellow solid (23.0 mg, 23%). Mp: 305–307 °C; IR (KBr): 3.425 (NH), 1.712 (C=O), 1.679 (C=O), 1.619 (C=C), 1.487 (NO_2_), 1.328 (NO_2_) cm^−1^; ^1^H NMR: *δ* 7.25 (1H, t, *J* = 7.5 Hz, Ph 5-H), 7.35–7.44 (2H, m, Ph-H), 7.56–7.74 (2H, m, C7-H & Ph-H), 8.17 (1H, d, *J* = 7.3 Hz, C8-H), 8.50 (1H, d, *J* = 7.3 Hz, C6-H), 9.19 (1H, s, C5-H), 11.36 (1H, s, N3-H); ^13^C NMR: *δ* 116.72 (CH, d, *J* = 19.6 Hz), 117.13 (Cq), 124.05 (Cq), 125.11 (CH), 125.21 (Cq), 125.34 (Cq), 125.36 (CH), 130.03 (CH), 132.60 (CH), 133.12 (CH, d, *J* = 8.0 Hz), 136.17 (CH), 140.85 (Cq), 142.95 (CH), 156.53 (Cq), 158.52 (Cq, d, *J* = 252.4 Hz), 159.51 (Cq), 161.71 (Cq); anal. RP-HPLC: *t*_R_ 1.10 min (100%, A), 3.98 min (97.6%, B); HRMS (ESI^+^): calcd for C_17_H_10_FN_4_O_4_ [M+H]^+^ 353.0681, found 353.0676.

### 5.9 10-(4-Chlorophenyl)-9-nitro-2H,3H,4H,10H-pyrimido[4, 5-b]quinoline-2,4-dione (7)

Prepared using general method 5.7 from **3a** (R^5,6^ = H, R^7^ = Cl; 64 mg, 0.27 mmol) and **4** (R^1–3^ = H, R^4^ = NO_2_, X = OTs; 104 mg, 0.32 mmol).^44^, ^45^ Yellow solid (23.8 mg, 24%). Mp 336–338 °C; IR (KBr): 3.422 (NH), 1.706 (C=O), 1.672 (C=O), 1.620 (C=C), 1.427 (NO_2_), 1,352 (NO_2_) cm^−1^; ^1^H NMR: *δ* 7.41 (2H, d, *J* = 8.6 Hz, Ph 2-H), 7.55 (2H, d, *J* = 8.6 Hz, Ph 3-H), 7.64 (1H, t, *J* = 7.8 Hz, C7-H), 8.13 (1H, d, *J* = 7.8 Hz, C8-H), 8.48 (1H, d, *J* = 7.8 Hz, C6-H), 9.17 (1H, s, C5-H), 11.30 (1H, s, N3-H); ^13^C NMR: *δ* 117.45 (Cq), 123.98 (Cq), 124.87 (CH), 129.21 (CH), 131.40 (CH), 132.03 (CH), 132.52 (Cq), 134.63 (Cq), 135.89 (CH), 136.24 (Cq), 141.09 (Cq), 142.62 (CH), 156.53 (Cq), 160.37 (Cq), 161.86 (Cq); anal. RP-HPLC: *t*_R_ 1.63 min (98.4%, A), 4.75 min (100%, B); HRMS (ESI^+^): calcd for C_17_H_10_ClN_4_O_4_ [M+H]^+^ 369.0391, found 369.0419.

### 5.10 10-Phenyl-9-(trifluoromethyl)-2H,3H,4H,10H-pyrimido [4,5-*b*]quinoline-2,4-dione (8)

Prepared using general method 5.7 from **3a** (R^5^^–^^7^ = H; 58 mg, 0.28 mmol)^46^ and **4** (R^1–3^ = H, R^4^ = CF_3_, X = F; 65 mg, 0.34 mmol). Yellow solid (23.2 mg, 23%). Mp 312–314 °C; IR (KBr): 3.424 (NH), 1.716 (C=O), 1.659 (C=O), 1.618 (C=C) cm^−1^; ^1^H NMR: *δ* 7.30–7.36 (2H, m, Ph 2-H), 7.45–7.51 (3H, m, Ph-H), 7.66 (1H, t, *J* = 7.7 Hz, C7-H), 8.18 (1H, dd, ^4^*J* = 1.4 Hz, ^3^*J* = 7.7 Hz, C8-H), 8.48 (1H, dd, ^4^*J* = 1.4 Hz, ^3^*J* = 7.7 Hz, C6-H), 9.11 (1H, s, C5-H), 11.23 (1H, s, N3-H); ^13^C NMR: *δ* 116.79 (Cq), 120.91 (CH), 124.26 (Cq), 124.94 (CH), 128.44 (CH), 129.58 (CH), 129.90 (CH), 131.20 (CH), 136.79 (Cq), 139.46 (Cq), 139.67 (Cq), 143.32(CH), 156.80 (Cq), 161.18 (Cq), 162.07 (Cq); anal. RP-HPLC: *t*_R_ 2.12 min (100%, A), 5.20 min (100%, B); HRMS (ESI^+^): calcd for C_18_H_11_F_3_N_3_O_2_ [M+H]^+^ 358.0803, found 358.0765.

### 5.11 10-(2-Fluorophenyl)-8-(trifluoromethyl)-2H,3H,4H, 10H-pyrimido[4,5-*b*]quinoline-2,4-dione (9)

Prepared using general method 5.7 from **3a** (R^5^ = F, R^6,7^ = H; 59 mg, 0.27 mmol)^8^ and **4** (R^1,2,4^ = H, R^3^ = CF_3_, X = F; 61 mg, 0.32 mmol). Yellow solid (39.1 mg, 39%). Mp 315–318 °C; IR (KBr): 3.423 (NH), 1.708 (C=O), 1.672 (C=O), 1.622 (C=C) cm^−1^; ^1^H NMR: *δ* 6.90 (1H, s, C9-H), 7.56 (1H, t, *J* = 7.6 Hz, Ph 5-H), 7.62–7.69 (2H, m, Ph-H), 7.73–7.80(1H, m, Ph-H), 7.89 (1H, d, *J* = 8.4 Hz, C7-H), 8.51 (1H, d, *J* = 8.4 Hz, C6-H), 9.23 (1H, s, C5-H), 11.34 (1H, s, N3-H); ^13^C NMR: *δ* 112.97 (CH, d, *J* = 4.77 Hz), 117.88 (CH, d, *J* = 18.40 Hz), 118.28 (Cq), 121.10 (CH, d, *J* = 3.62 Hz), 123.86 (Cq), 124.29 (Cq, d, *J* = 13.29 Hz), 126.81 (CH, d, *J* = 3.52 Hz), 130.94 (CH), 133.06 (CH, *d*, *J* = 8.09 Hz), 133.93 (CH), 134.27 (Cq), 141.12 (Cq), 142.34 (CH), 156.63 (Cq), 157.58 (Cq, d, *J* = 251.92 Hz), 159.17 (Cq), 161.77 (Cq); anal. RP-HPLC: *t*_R_ 3.59 min (97.3%, A), 6.42 min (100%, B); HRMS (ESI^+^): calcd for C_18_H_10_F_4_N_3_O_2_ [M+H]^+^ 376.0709, found 376.0705.

### 5.12 10-(2-Fluorophenyl)-9-(trifluoromethyl)-2H,3H,4H, 10H-pyrimido[4,5-*b*]quinoline-2,4-dione (10)

Prepared using general method 5.7 from **3a** (R^5^ = F, R^6,7^ = H; 59 mg, 0.27 mmol)^8^ and **4** (R^1–3^ = H, R^4^ = CF_3_, X = F; 62 mg, 0.32 mmol). Yellow solid (65.7 mg, 66%). Mp: 292–293 °C (lit.^8^ >360 °C); IR (KBr): 3.418 (NH), 1.703 (C=O), 1.679 (C=O), 1,631 (C=C) cm^−1^; ^1^H NMR: *δ* 7.25–7.31(2H, m, Ph-H), 7.42 (1H, t, *J* = 9.7 Hz, Ph 5-H), 7.55–7.63 (1H, m, Ph-H), 7.69 (1H, t, *J* = 7.9 Hz, C7-H), 8.23 (1H, dd, ^4^*J* = 1.3 Hz, ^3^*J* = 7.9 Hz, C8-H), 8.52 (1H, dd, ^4^*J* = 1.3 Hz, ^3^*J* = 7.9 Hz, C6-H), 9.15 (1H, s, C5-H), 11.32 (1H, s, N3-H); ^13^C NMR: *δ* 115.80 (CH, d, *J* = 20.1 Hz), 116.05 (CH), 123.89 (Cq), 124.64 (Cq), 126.75 (CH, d, *J* = 3.6 Hz), 129.70 (CH), 130.95 (CH), 131.14 (Cq), 131.99 (CH, d, *J* = 7.64 Hz), 135.36 (Cq), 136.91 (Cq), 139.57 (Cq), 143.28 (CH), 156.26 (Cq), 158.77 (Cq, d, *J* = 251.8 Hz), 159.99 (Cq), 161.45 (Cq); anal. RP-HPLC: *t*_R_ 2.52 min (98.0%, A), 5.62 min (97.5%, B); HRMS (ESI^+^): calcd for C_18_H_10_F_4_N_3_O_2_ [M+H]^+^ 376.0709, found 376.0729.

### 5.13 10-(4-Chlorophenyl)-6-(trifluoromethyl)-2H,3H,4H, 10H-pyrimido[4,5-*b*]quinoline-2,4-dione (11)

Prepared using general method 5.7 from **3a** (R^5,6^ = H, R^7^ = Cl; 61 mg, 0.26 mmol)^25^ and **4** (R^1^ = CF_3_, R^2^^–^^4^ = H, X = F; 60 mg, 0.31 mmol). Yellow solid (11.1 mg, 11%). Mp 304–306 °C; IR (KBr): 3.410 (NH), 1.716 (C = O), 1.670 (C=O), 1624 (C=C) cm^−1^; ^1^H NMR: *δ* 7.16 (1H, d, *J* = 8.0 Hz, C9-H), 7.52 (2H, d, *J* = 8.8 Hz, Ph 2-H), 7.80 (2H, d, *J* = 8.8 Hz, Ph 3-H), 7.88 (1H, t, *J* = 8.0 Hz, C8-H), 7.97 (1H, d, *J* = 8.0 Hz, C7-H), 8.86 (1H, s, C5-H), 11.33 (1H, s, N3-H); ^13^C NMR: *δ* 116.89 (Cq), 118.04 (Cq), 122.94 (CH), 123.27 (CH), 127.48 (Cq), 130.86 (CH), 130.99 (CH), 134.66 (CH), 134.83 (Cq), 135.64 (CH), 136.70 (Cq), 143.28 (Cq), 156.53 (Cq), 158.80 (Cq), 161.88 (Cq); anal. RP-HPLC: *t*_R_ 3.98 min (100%, A), 7.47 min (96.2%, B); HRMS (ESI^+^): calcd for C_18_H_10_ClF_3_N_3_O_2_ [M+H]^+^ 392.0414, found 392.0439.

### 5.14 10-(4-Chlorophenyl)-8-(trifluoromethyl)-2H,3H,4H, 10H-pyrimido[4,5-*b*]quinoline-2,4-dione (13)

Prepared using general method 5.7 from **3a** (R^5,6^ = H, R^7^ = Cl; 84 mg, 0.35 mmol)^25^ and **4** (R^1,2,4^ = H, R^3^ = CF_3_, X = F; 81 mg, 0.42 mmol). Yellow solid (70.8 mg, 51%). Mp 348–349 °C (dec); IR (KBr): 3.430 (NH), 1.716 (C=O), 1.683 (C=O), 1.650 (C=C) cm^−1^; ^1^H NMR: *δ* 6.89 (1H, s, C9-H), 7.54 (2H, d, *J* = 8.7 Hz, Ph 2-H), 7.81(2H, d, *J* = 8.7 Hz, Ph 3-H), 7.86 (1H, d, *J* = 8.2 Hz, C7-H), 8.49 (1H, d, *J* = 8.2 Hz, C6-H), 9.22 (1H, s, C5-H), 11.28 (1H, s, N3-H); ^13^C NMR: *δ* 118.40 (CH), 120.69 (CH), 120.72 (CH), 122.36 (Cq), 123.96 (Cq), 130.94 (CH), 131.06 (CH), 133.59 (CH), 134.92 (Cq), 136.31 (Cq), 141.79 (Cq), 141.94 (CH), 156.67 (Cq), 159.58 (Cq), 161.97 (Cq); anal. RP-HPLC: *t*_R_ 6.18 min (100%, A), 8.60 min (97.4%, B). HRMS (ESI^+^): calcd for C_18_H_10_ClF_3_N_3_O_2_ [M+H]^+^ 392.0414, found 392.0374.

### 5.15 10-(4-Chlorophenyl)-9-(trifluoromethyl)-2H,3H,4H, 10H-pyrimido[4,5-*b*]quinoline-2,4-dione (14)

Prepared using general method 5.7 from **3a** (R^5,6^ = H, R^7^ = Cl; 61 mg, 0.26 mmol)^25^ and **4** (R^1^^–^^3^ = H, R^4^ = CF_3_, X = F; 60 mg, 0.31 mmol). Yellow solid (28.3 mg, 28%). Mp 372–375 °C; IR (KBr): 3.483 (NH), 1.715 (C=O), 1.659 (C=O), 1.618 (C=C) cm^−1^; ^1^H NMR: *δ* 7.39 (2H, d, *J* = 8.6 Hz, Ph 2-H), 7.56 (2H, d, *J* = 8.6 Hz, Ph 3-H), 7.67 (1H, t, *J* = 7.7 Hz, C7-H), 8.20 (1H, dd, ^4^*J* = 1.3 Hz, ^3^*J* = 7.7 Hz, C8-H), 8.49 (1H, dd, ^4^*J* = 1.3 Hz, ^3^*J* = 7.7 Hz, C6-H), 9.11 (1H, s, C5-H),11.25 (1H, s, N3-H); ^13^C NMR: *δ* 116.76 (Cq), 118.70 (Cq), 124.38 (Cq), 124.98 (CH), 128.57 (CH), 133.16 (CH), 134.23 (Cq), 135.60 (CH), 137.03 (CH), 139.24 (Cq), 140.15 (Cq), 143.47 (CH), 156.69 (Cq), 161.14 (Cq), 161.97 (Cq); anal. RP-HPLC: *t*_R_ 4.37 min (100%, A), 7.17 min (98.2%, B); HRMS (ESI^+^): calcd for C_18_H_10_ClF_3_N_3_O_2_ [M+H]^+^ 392.0414, found 392.0416.

### 5.16 10-(3-Chlorophenyl)-9-(trifluoromethyl)-2H,3H,4H, 10H-pyrimido[4,5-*b*]quinoline-2,4-dione (15)

Prepared using general method 5.7 from **3a** (R^5,7^ = H, R^6^ = Cl; 60 mg, 0.25 mmol)^8^ and **4** (R^1–3^ = H, R^4^ = CF_3_, X = F; 58 mg, 0.3 mmol). Yellow solid (54.6 mg, 55%). Mp: 342–343 °C (dec); IR (KBr): 3.421 (NH), 1.706 (C=O), 1.672 (C=O), 1.623 (C=C) cm^−1^; ^1^H NMR: *δ* 7.37 (1H, d, *J* = 7.7 Hz, Ph-H), 7.48–7.62 (3H, m, Ph-H), 7.68 (1H, t, *J* = 7.5 Hz, C7-H), 8.21 (1H, d, *J* = 7.5 Hz, C8-H), 8.49 (1H, d, *J* = 7.5 Hz, C6-H), 9.11 (1H, s, C5-H), 11.28 (1H, s, N3-H); ^13^C NMR: *δ* 116.76 (Cq), 124.45 (Cq), 125.03 (CH), 129.72 (CH), 130.04 (CH), 130.44 (CH), 131.19 (CH), 132.58 (Cq), 135.61 (CH), 135.66 (Cq), 137.06 (CH), 140.04 (Cq), 141.39 (Cq), 143.56 (CH), 156.70 (Cq), 161.16 (Cq), 161.95 (Cq); anal. RP-HPLC: *t*_R_ 3.54 min (97.8%, A), 8.69 min (98.9%, B); HRMS (ESI^+^): calcd for C_18_H_10_ClF_3_N_3_O_2_ [M+H]^+^ 392.0408, found 392.0404.

### 5.17 10-(3,4-Dichlorophenyl)-9-(trifluoromethyl)-2H,3H,4H, 10H-pyrimido[4,5-*b*]quinoline-2,4-dione (16)

Prepared using general method 5.7 from **3a** (R^5^ = H, R^6,7^ = Cl; 64 mg, 0.24 mmol)^47^ and **4** (R^1–3^ = H, R^4^ = CF_3_, X = F; 55 mg, 0.29 mmol). Yellow solid (62.3 mg, 62%). Mp 320–322 °C; IR (KBr): 3.486 (NH), 1.705 (C=O), 1.655 (C=O), 1.609 (C=C) cm^−1^; ^1^H NMR: *δ* 7.46 (1H, dd, ^4^*J* = 1.1 Hz, ^3^*J* = 8.6 Hz, Ph 6-H), 7.69 (1H, t, *J* = 7.8 Hz, C7-H), 7.73 (1H, d, ^4^*J* = 1.1 Hz, Ph 2-H), 7.81 (1H, d, *J* = 8.6 Hz, Ph 5-H), 8.23 (1H, dd, ^4^*J* = 1.6 Hz, ^3^*J* = 7.8 Hz, C8-H), 8.50 (1H, dd, ^4^*J* = 1.6 Hz, ^3^*J* = 7.8 Hz, C6-H), 9.12 (1H, s,C5-H), 11.30 (1H, s, N3-H); ^13^C NMR: *δ* 116.71 (Cq), 118.33 (Cq), 121.67 (Cq), 124.46 (Cq), 125.13 (CH), 130.37 (CH), 130.94 (Cq), 132.28 (CH), 132.58 (Cq), 133.14 (CH), 135.71 (CH), 137.30 (CH), 139.84 (Cq), 143.71 (CH), 156.61 (Cq), 161.14 (Cq), 161.88 (Cq); anal. RP-HPLC: *t*_R_ 6.40 min (99.4%, A), 6.35 min (98.7%, B) HRMS (ESI^+^): calcd for C_18_H_9_Cl_2_F_3_N_3_O_2_ [M+H]^+^ 426.0024, found 426.0034.

### 5.18 10-(4-Fluorophenyl)-9-(trifluoromethyl)-2H,3H,4H, 10H-pyrimido[4,5-*b*]quinoline-2,4-dione (18)

Prepared using general method 5.7 from **3a** (R^5,6^ = H, R^7^ = F; 58 mg, 0.26 mmol)^8^ and **4** (R^1–3^ = H, R^4^ = CF_3_, X = F; 60 mg, 0.31 mmol). Yellow solid (45.5 mg, 46%). Mp: >350 °C (lit.^8^ 327–329 °C); IR (KBr): 3.420 (NH), 1.716 (C=O), 1.660 (C=O), 1.620 (C=C) cm^−1^; ^1^H NMR: *δ* 7.29–7.36 (2H, m, Ph 2-H), 7.38–7.45 (2H, m, Ph 3-H), 7.67 (1H, t, *J* = 7.4 Hz, C7-H), 8.20 (1H, d, *J* = 7.4 Hz, C8-H), 8.49 (1H, d, *J* = 7.4 Hz, C6-H), 9.11 (1H, s, C5-H), 11.24 (1H, s, N3-H); ^13^C NMR: *δ* 115.41 (CH, d, *J* = 23.0 Hz), 116.76 (Cq), 118.60 (Cq), 118.91 (Cq), 124.34 (Cq), 124.88 (CH), 133.52 (CH, d, *J* = 9.3 Hz), 135.57 (CH), 136.97 (CH), 140.44 (Cq), 143.42 (CH), 156.75 (Cq), 162.00 (Cq), 161.31 (Cq), 162.27 (Cq, d, *J* = 247.93 Hz); anal. RP-HPLC: *t*_R_ 2.25 min (100%, A), 27.65 min (100%, B); HRMS (ESI^+^): calcd for C_18_H_10_F_4_N_3_O_2_ [M+H]^+^ 376.0709, found 376.0719.

### 5.19 10-(3-Methylphenyl)-9-(trifluoromethyl)-2H,3H,4H, 10H-pyrimido[4,5-*b*]quinoline-2,4-dione (19)

Prepared using general method 5.7 from **3a** (R^5,7^ = H, R^6^ = Me; 58 mg, 0.27 mmol)^43^ and **4** (R^1–3^ = H, R^4^ = CF_3_, X = F; 62 mg, 0.32 mmol). Yellow solid (38.6 mg, 39%). Mp 296–298 °C (dec); IR (KBr): 3.474 (NH), 1.716 (C=O), 1.657 (C=O), 1.619 (C=C) cm^−1^; ^1^H NMR: *δ* 2.31 (3H, s, Me), 7.08 (1H, s, Ph 2-H), 7.18 (1H, d, *J* = 7.6 Hz, Ph 4-H), 7.29 (1H, d, *J* = 7.6 Hz, Ph 3-H), 7.36 (1H, t, *J* = 7.6 Hz, Ph 5-H), 7.66 (1H, t, *J* = 7.6 Hz, C7-H), 8.18 (1H, dd, ^4^*J* = 1.5 Hz, ^3^*J* = 7.6 Hz, C8-H), 8.47 (1H, dd, ^4^*J* = 1.5 Hz, ^3^*J* = 7.6 Hz, C6-H), 9.10 (1H, s, C5-H), 11.22 (1H, s, N3-H); ^13^C NMR: *δ* 21.78 (CH_3_), 116.71 (Cq), 118.95 (Cq), 119.27 (Cq), 124.28 (Cq), 124.82 (CH), 128.12 (CH), 128.66 (CH), 130.10 (CH), 131.11 (CH), 135.51 (CH), 136.79 (CH), 137.96 (Cq), 140.49 (Cq), 143.29 (CH), 156.85 (Cq), 161.23 (Cq), 162.05 (Cq); anal. RP-HPLC: *t*_R_ 3.00 min (100%, A), 4.64 min (99.7%, B); HRMS (ESI^+^): calcd for C_19_H_13_F_3_N_3_O_2_ [M+H]^+^ 372.0960, found 372.0954.

### 5.20 10-(4-Methylphenyl)-9-(trifluoromethyl)-2H,3H,4H, 10H-pyrimido[4,5-*b*]quinoline-2,4-dione (20)

Prepared using method 5.7 from **3a** (R^5,6^ = H, R^7^ = Me; 59 mg, 0.27 mmol)^8^ and **4** (R^1–3^ = H, R^4^ = CF_3_, X = F; 62 mg, 0.32 mmol). Yellow solid (52.1 mg, 52%). Mp 344–346 °C (dec); IR (KBr): 3.438 (NH), 1.706 (C=O), 1.681 (C=O), 1.622 (C=C) cm^−1^; ^1^H NMR: *δ* 2.38 (3H, s, Me), 7.19 (2H, d, *J* = 8.4 Hz, Ph 3-H), 7.27 (2H, d, *J* = 8.4 Hz, Ph 2-H), 7.65 (1H, t, *J* = 7.9 Hz, C7-H), 8.17 (1H, dd, ^4^*J* = 1.3 Hz, ^3^*J* = 7.9 Hz, C8-H), 8.47 (1H, dd, ^4^*J* = 1.3 Hz, ^3^*J* = 7.9 Hz, C6-H), 9.09 (1H, s, C5-H), 11.21 (1H, s, N3-H); ^13^C NMR: *δ* 21.27 (CH_3_), 116.72 (Cq), 118.94 (Cq), 124.30 (Cq), 124.80 (CH), 128.94 (CH), 130.92 (CH), 135.52 (CH), 136.79 (CH), 138.18 (Cq), 139.12 (Cq), 140.56 (Cq), 143.28 (CH), 156.80 (Cq), 161.29 (Cq), 162.07 (Cq); anal. RP-HPLC: *t*_R_ 3.13 min (100%, A), 8.57 min (99.7%, B); HRMS (ESI^+^): calcd for C_19_H_13_F_3_N_3_O_2_ [M+H]^+^ 372.0960, found 372.0957.

### 5.21 10-Benzyl-9-(trifluoromethyl)-2H,3H,4H,10H-pyrimido-[4,5-*b*]quinoline-2,4-dione (21)

Prepared using method 5.7 from **3b** (R^9^ = Bn; 56 mg, 0.26 mmol)^48^ and **4** (R^1–3^ = H, R^4^ = CF_3_, X = F; 60 mg, 0.31 mmol). Yellow solid (11.5 mg, 12%). Mp 235–237 °C; IR (KBr): 3.416 (NH), 1.706 (C=O), 1.664 (C=O), 1.615 (C=C) cm^−1^; ^1^H NMR: *δ* 5.87 (2H, s, CH_2_), 6.81 (2H, d, *J* = 6.7 Hz, Ph 2-H), 7.13–7.21 (3H, m, Ph-H), 7.68 (1H, t, *J* = 7.8 Hz, C7-H), 8.31 (1H, dd, ^4^*J* = 1.3 Hz, ^3^*J* = 7.8 Hz, C8-H), 8.47 (1H, d, *J* = 7.8 Hz, C6-H), 9.02 (1H, s, C5-H), 11.26 (1H, s, N3-H); ^13^C NMR: *δ* 54.41 (CH_2_), 117.14 (Cq), 118.17 (Cq), 124.54 (Cq), 125.08 (CH), 126.70 (CH), 127.62 (CH), 128.86 (CH), 135.99 (CH), 136.69 (Cq), 137.34 (CH), 139.88 (Cq), 142.47 (CH), 156.73 (Cq), 160.83 (Cq), 162.02 (Cq); anal. RP-HPLC: *t*_R_ 2.34 min (97.5%, A), 8.22 min (96.8%, B); HRMS (ESI^+^): calcd for C_19_H_13_F_3_N_3_O_2_ [M+H]^+^ 372.0960, found 372.0973.

### 5.22 9-Chloro-10-phenyl-2H,3H,4H,10H-pyrimido[4,5-*b*]quinoline-2,4-dione (22)

Prepared using method 5.7 from **3a** (R^5^^–^^7^ = H; 62 mg, 0.31 mmol)^46^ and **4** (R^1–3^ = H, R^4^ = CF_3_, X = F; 59 mg, 0.37 mmol). Yellow solid (38.8 mg, 39%). Mp > 350 °C. IR (KBr): 3.441 (NH), 1.717 (C=O), 1.659 (C=O), 1.615 (C=C) cm^−1^; ^1^H NMR: *δ* 7.37 (2H, m, Ph 2-H), 7.45–7.54 (3H, m, Ph-H), 7.48 (1H, t, *J* = 7.8 Hz, C7-H), 7.83 (1H, dd, ^4^*J* = 1.5 Hz, ^3^*J* = 7.8 Hz, C8-H), 8.23 (1H, dd, ^4^*J* = 1.5 Hz, ^3^*J* = 7.8 Hz, C6-H), 9.10 (1H, s, C5-H), 11.18 (1H, s, N3-H); ^13^C NMR: *δ* 116.42 (Cq), 121.22 (Cq), 124.57 (Cq), 125.70 (CH), 129.10 (CH), 129.45 (CH), 130.33 (CH), 132.34 (CH), 137.67 (Cq), 139.26 (CH), 139.62 (Cq), 143.26 (CH), 156.75 (Cq), 160.75 (Cq), 162.08 (Cq); anal. RP-HPLC: *t*_R_ 1.53 min (97.2%, A), 4.59 min (100%, B); HRMS (ESI^+^): calcd for C_17_H_11_ClN_3_O_2_ [M+H]^+^ 324.0540, found 324.0513.

### 5.23 7-Chloro-10-(2-fluorophenyl)-2H,3H,4H,10H-pyrimido-[4,5-*b*]quinoline-2,4-dione (23)

Prepared using method 5.7 from **3a** (R^5^ = F, R^6,7^ = H; 65 mg, 0.29 mmol)^8^ and **4** (R^1–3^ = H, R^2^ = Cl, X = Cl; 55 mg, 0.35 mmol). Yellow solid (79.1 mg, 79%). Mp 329–330 °C; IR (KBr): 3.427 (NH), 1.707 (C=O), 1.652 (C=O), 1.615 (C=C) cm^−1^; ^1^H NMR: *δ* 6.84 (1H, d, *J* = 9.1 Hz, C9-H), 7.51–7.66 (3H, m, Ph-H), 7.71–7.77 (1H, m, Ph-H), 7.80 (1H, dd, ^3^*J* = 2.5 Hz, ^4^*J* = 9.1 Hz, C8-H), 8.41 (1H, d, ^4^*J* = 2.5 Hz, C6-H), 9.12 (1H, s, C5-H), 11.26 (1H, s, N3-H); ^13^C NMR: *δ* 117.05 (CH), 117.75 (CH, d, *J* = 19.1 Hz), 118.85 (CH), 122.46 (Cq), 124.72 (Cq, d, *J* = 13.6 Hz), 126.68 (CH), 129.30 (Cq), 130.67 (CH), 130.93 (CH), 132.75 (CH, d, *J* = 7.5 Hz), 135.54 (CH), 140.08 (Cq), 142.25 (CH), 157.72 (Cq), 157.57 (Cq, d, *J* = 251.6 Hz), 158.79 (Cq), 161.92 (Cq); anal. RP-HPLC: *t*_R_ 2.05 min (100%, A), 5.34 min (98.6%, B); HRMS (ESI^+^): calcd for C_17_H_10_ClFN_3_O_2_ [M+H]^+^ 342.0446, found 342.0399.

### 5.24 6-Chloro-10-(4-chlorophenyl)-2H,3H,4H,10H-pyrimido- [4,5-*b*]quinoline-2,4-dione (24)

Prepared using general method 5.7 from **3a** (R^5,6^ = H, R^7^ = Cl; 66 mg, 0.28 mmol)^25^ and **4** (R^1^ = Cl, R^2–4^ = H, X = F; 53 mg, 0.34 mmol). Yellow solid, (37.6 mg, 38%). Mp: 331–332 °C (lit.^8^ 220–222 °C, dec); IR (KBr): 3.422 (NH), 1.703 (C=O), 1.672 (C=O), 1.607 (C=C) cm^−1^; ^1^H NMR: δ 6.77 (1H, dd, ^4^*J* = 2.3 Hz, ^3^*J* = 7.1 Hz, C9-H), 7.48 (2H, d, *J* = 8.7 Hz, Ph 2-H), 7.68–7.71 (2H, m, C7-H & C8-H), 7.78 (2H, d, *J* = 8.7 Hz, Ph 3-H), 9.01 (1H, s, C5-H), 11.27 (1H, s, N3-H); ^13^C NMR: *δ* 117.22 (Cq), 117.30 (CH), 118.97 (Cq), 125.42 (CH), 130.86 (CH), 130.90 (CH), 134.25 (Cq), 134.72 (Cq), 135.78 (CH), 136.85 (Cq), 137.08 (CH), 143.50 (Cq), 156.69 (Cq), 159.08 (Cq), 162.04 (Cq); anal. RP-HPLC: *t*_R_ 2.50 min (99.6%; A), 6.05 min (100%, B); HRMS (ESI^+^): calcd for C_17_H_10_Cl_2_N_3_O_2_ [M+H]^+^ 358.0150, found 358.0155.

### 5.25 9-Chloro-10-(4-chlorophenyl)-2H,3H,4H,10H-pyrimido-[4,5-*b*]quinoline-2,4-dione (25)

Prepared using general method 5.7 from **3a** (R^5,6^ = H, R^7^ = Cl; 65 mg, 0.27 mmol)^25^ and **4** (R^1–3^ = H, R^4^ = Cl, X = F; 51 mg, 0.32 mmol). Yellow solid (14.6 mg, 15%). Mp > 350 °C; IR (KBr): 3.415 (NH), 1.715 (C=O), 1.655 (C=O), 1.613 (C=C) cm^−1^; ^1^H NMR: *δ* 7.43–7.52 (3H, m, C7-H & Ph 2-H), 7.60 (2H, d, *J* = 8.8 Hz, Ph 3-H), 7.85 (1H, dd, ^4^*J* = 1.5 Hz, ^3^*J* = 7.9 Hz, C8-H), 8.28 (1H, dd, ^4^*J* = 1.5 Hz, ^3^*J* = 7.9 Hz, C6-H), 9.09 (1H, s, C5-H), 11.22 (1H, s, N3-H); ^13^C NMR: *δ* 116.48 (Cq), 121.00 (Cq), 124.59 (Cq), 125.77 (CH), 129.18 (CH), 132.22 (CH), 132.45 (CH), 134.12 (Cq), 137.53 (Cq), 138.57 (Cq), 139.25 (CH), 143.33 (CH), 156.63 (Cq), 160.81 (Cq), 162.04 (Cq); anal. RP-HPLC: *t*_R_ 3.10 min (100%; A), 6.34 min (97.2%; B); HRMS (ESI^+^): calcd for C_17_H_10_Cl_2_N_3_O_2_ [M+H]^+^ 358.0150, found 358.0156.

### 5.26 9-Chloro-10-(3-chlorophenyl)-2H,3H,4H,10H-pyrimido-[4,5-*b*]quinoline-2,4-dione (26)

Prepared using general method 5.7 from **3a** (R^5,7^ = H, R^6^ = Cl, 65 mg, 0.27 mmol)^8^ and **4** (R^1–3^ = H, R^4^ = Cl, X = F; 51 mg, 0.32 mmol). Yellow solid (29.3 mg, 30%). Mp > 350 °C; IR (KBr): 3.416 (NH), 1.715 (C=O), 1.655 (C=O), 1.613 (C=C) cm^−1^; ^1^H NMR: *δ* 7.41–7.45 (1H, m, Ph-H), 7.49 (1H, t, *J* = 7.6 Hz, Ph 5-H), 7.56 (1H, t, *J* = 8.1 Hz, C7-H), 7.60–7.64 (2H, m, Ph-H), 7.86 (1H, dd, ^4^*J* = 1.2 Hz, ^3^*J* = 8.1 Hz, C8-H), 8.25 (1H, dd, ^4^*J* = 1.2 Hz, ^3^*J* = 8.1 Hz, C6-H), 9.10 (1H, s, C5-H), 11.21 (1H, s, N3-H); ^13^C NMR: *δ* 116.39 (Cq), 120.84 (Cq), 124.56 (Cq), 125.82 (CH), 129.40 (CH), 129.60 (CH), 130.35 (CH), 130.62 (CH), 132.46 (CH), 133.31 (Cq), 137.39 (Cq), 139.32 (CH), 140.86 (Cq), 143.46 (CH), 156.62 (Cq), 160.75 (Cq), 161.96 (Cq); anal. RP-HPLC: *t*_R_ 2.48 min (100%; A), 8.60 min (98.3%; B); HRMS (ESI^+^): calcd for C_17_H_10_Cl_2_N_3_O_2_ [M+H]^+^ 358.0150, found 358.0151.

### 5.27 9-Chloro-10-(3-fluorophenyl)-2H,3H,4H,10H-pyrimido-[4,5-*b*]quinoline-2,4-dione (27)

Prepared using general method 5.7 from **3a** (R^5,7^ = H, R^6^ = F; 65 mg, 0.29 mmol)^8^ and **4** (R^1–3^ = H, R^4^ = Cl, X = F; 55 mg, 0.35 mmol). Yellow solid (71.0 mg, 71%). Mp > 365 °C; IR (KBr): 3.417 (NH), 1.717 (C=O), 1.658 (C=O), 1.614 (C=C) cm^−1^; ^1^H NMR: *δ* 7.28 (1H, d, *J* = 7.8 Hz, Ph 6-H), 7.38–7.45 (2H, m, Ph-H), 7.49 (1H, t, *J* = 7.8 Hz, C7-H), 7.53–7.60 (1H, m, Ph-H), 7.86 (1H, dd, ^4^*J* = 1.3 Hz, ^3^*J* = 7.8 Hz, C8-H), 8.25 (1H, dd, ^4^*J* = 1.3 Hz, ^3^*J* = 7.8 Hz, C6-H), 9.10 (1H, s, C5-H), 11.21 (1H, s, N3-H); ^13^C NMR: *δ* 116.38 (Cq), 116.59 (CH, d, *J* = 21.2 Hz), 118.11 (CH, d, *J* = 23.8 Hz), 120.94 (Cq), 124.55 (Cq), 125.82 (CH), 126.86 (CH, d, *J* = 2.9 Hz), 130.58 (CH, d, *J* = 8.8 Hz), 132.45 (CH), 137.43 (Cq), 139.33 (CH), 140.88 (Cq, d, *J* = 10.9 Hz), 143.42 (CH), 156.64 (Cq), 160.73 (Cq), 161.96 (Cq), 162.35 (Cq, d, *J* = 243.7 Hz); anal. RP-HPLC: *t*_R_ 1.63 min (97.0%; A), 19.62 min (98.1%; B); HRMS (ESI^+^): calcd for C_17_H_10_ClFN_3_O_2_ [M+H]^+^ 342.0446, found 342.0453.

### 5.28 10-(4-Chlorophenyl)-8-methyl-2H,3H,4H,10H-pyrimido [4,5-*b*]quinoline-2,4-dione (28)

Prepared using general method 5.7 from **3a** (R^5,6^ = H, R^7^ = Cl; 71 mg, 0.3 mmol)^25^ and **4** (R^1,2,4^ = H, R^3^ = Me, X = OTs; 105 mg, 0.36 mmol). Yellow solid (24.3 mg, 24%). Mp 346–347 °C (dec); IR (KBr): 3.441 (NH), 1.694 (C=O), 1.663 (C=O), 1.602 (C=C) cm^−1^; ^1^H NMR: *δ* 2.36 (3H, s, Me), 6.57 (1H, s, C9-H), 7.36 (1H, d, *J* = 8.1 Hz, C7-H), 7.47 (2H, d, *J* = 8.5 Hz, Ph 2-H), 7.76 (2H, d, *J* = 8.5 Hz, Ph 3-H), 8.12 (1H, d, *J* = 8.1 Hz, C6-H), 9.08 (1H, s, C5-H), 11.04 (1H, s, N3-H); ^13^C NMR: *δ* 22.58 (CH_3_), 115.06 (Cq), 117.06 (CH), 119.64 (Cq), 126.66 (CH), 130.84 (CH), 130.99 (CH), 131.79 (CH), 134.46 (Cq), 136.95 (Cq), 142.29 (CH), 142.81 (Cq), 147.11 (Cq), 156.80 (Cq), 159.33 (Cq), 162.47 (Cq); anal. RP-HPLC: *t*_R_ 2.07 min (98.9%; A), 8.40 min (99.8%; B); HRMS (ESI^+^): calcd for C_18_H_13_ClN_3_O_2_ [M+H]^+^ 338.0696, found 338.0718.

### 5.29 9-Bromo-10-phenyl-2H,3H,4H,10H-pyrimido[4,5b]-qui-noline-2,4-dione (29)

Prepared using general method 5.7 from **3a** (R^5–7^ = H; 56 mg, 0.27 mmol) and **4** (R^1–3^ = H, R^4^ = Br, X = OTs; 115 mg, 0.32 mmol). Yellow solid (29.3 mg, 29%). Mp > 350 °C; IR (KBr): 3.413 (NH), 1.717 (C=O), 1.658 (C=O), 1.614 (C=C) cm^−1^; ^1^H NMR: *δ* 7.37–7.42 (3H, m), 7.51–7.55 (3H, m), 8.06 (1H, dd, ^4^*J* = 1.5 Hz, ^3^*J* = 7.8 Hz, C8-H), 8.27 (1H, dd, ^4^*J* = 1.5 Hz, ^3^*J* = 7.8 Hz, C6-H), 9.06 (1H, s, C5-H), 11.16 (1H, s, N3-H); ^13^C NMR: *δ* 109.28 (Cq), 116.31 (Cq), 124.76 (Cq), 126.06 (CH), 129.11 (CH), 129.63 (CH), 131.02 (Cq), 131.15 (CH), 132.87 (CH), 138.75 (CH), 138.88 (Cq), 143.19 (CH), 156.73 (Cq), 160.73 (Cq), 162.06 (Cq); anal. RP-HPLC: *t*_R_ 1.55 min (98.5%; A), 21.07 min (99.2%; B); HRMS (ESI^+^): calcd for C_17_H_11_BrN_3_O_2_ [M+H]^+^ 368.0035, found 367.9998.

### 5.30 9-Bromo-10-(4-chlorophenyl)-2H,3H,4H,10H-pyrimido-[4,5-*b*]quinoline-2,4-dione (30)

Prepared using general method 5.7 from **3a** (R^5,6^ = H, R^7^ = Cl, 60 mg, 0.25 mmol)^25^ and **4** (R^1–3^ = H, R^4^ = Br, X = OTs; 107 mg, 0.3 mmol). Yellow solid (36.4 mg, 36%). Mp > 350 °C; IR (KBr): 3.439 (NH), 1.714 (C=O), 1.655 (C=O), 1.610 (C=C) cm^−1^; ^1^H NMR: *δ* 7.40 (1H, t, *J* = 7.7 Hz, C7-H), 7.44 (2H, d, *J* = 8.7 Hz, Ph 2-H), 7.61 (2H, d, *J* = 8.7 Hz, Ph 3-H), 8.08 (1H, dd, ^4^*J* = 1.5 Hz, ^3^*J* = 7.7 Hz, C8-H), 8.27 (1H, dd, ^4^*J* = 1.5 Hz, ^3^*J* = 7.7 Hz, C6-H), 9.06 (1H, s, C5-H), 11.19 (1H, s, N3-H); ^13^C NMR: *δ* 109.12 (Cq), 116.30 (Cq), 124.76 (Cq), 126.15 (CH), 129.18 (CH), 132.95 (CH), 132.96 (CH), 134.33 (Cq), 137.72 (Cq), 138.72 (Cq), 143.19 (CH), 143.34 (CH), 156.68 (Cq), 160.84 (Cq), 162.04 (Cq); anal. RP-HPLC: *t*_R_ 2.73 min (97.6%; A), 4.17 min (100%; B); HRMS (ESI^+^): calcd for C_17_H_10_BrClN_3_O_2_ [M+H]^+^ 401.9645, found 401.9613.

### 5.31 Molecular modeling

Flexible docking was carried out using OMEGA, version 2.1.6, OEDocking, version 3.0.0, and VIDA, version 4.1.2, OpenEye Scientific Software, Inc., Santa Fe, NM, USA, www.eyesopen.com, 2012. Chain A (E3 ubiquitin-protein ligase domain of MDM2, residues 428–491) from PDB entry 2VJF was extracted and a docking receptor was constructed using the OEDocking application Make Receptor. The structure of the entire chain was set as the ligand box (23.0 Å × 29.7 Å × 25.7 Å; 17,513 Å^2^) and molecular cavity detection was used with default settings. The balanced binding site shape potential resulting from this included all protein surface cavities (total contour 2315 Å^2^), with an inner contour (53 Å^2^) encompassing the binding site of the C-terminus of HDMX (chain B ^488^FIA^490^) observed in 2VJF. Multiconformer databases of the bioactive compounds (Table 1) were created (OMEGA; default settings), and these were docked to the above receptor with default settings and no constraints. For each compound up to 10 binding poses were ranked using the Chemgauss 4 scoring function. The majority (>80%) of docking poses with scores <-3 kcal/mol placed the ligand at the C-terminal tripeptide HDMX binding site observed in 2VJF.

## Supplementary data

Supplementary data Supplementary Table S1, summarising inactive compounds **31**–**96**. ^1^H and ^13^C NMR spectra for compounds **10**, **18**, and **69** (with analytical and pharmacological data at variance with Wilson et al.^8^). Preparative and analytical data for pharmacologically inactive compounds **31**–**96**.
