# Supplementary material for: 5-Deazaflavin derivatives as inhibitors of p53 ubiquitination by HDM2
Source: Bioorg Med Chem. 2013 Nov 15;21(22):6868–77. doi: 10.1016/j.bmc.2013.09.038 (PMC3898830; doi:10.1016/j.bmc.2013.09.038)
Supplement: Supplementary data — Supplementary Table S1, summarising inactive compounds 31–96. 1H and 13C NMR spectra for compounds 10, 18, and 69 (with analytical and pharmacological data at variance with Wilson et al.8). Preparative and analytical data for pharmacologically inactive compounds 31–96. [file mmc1.doc]

Supplementary Data

5-Deazaflavin derivatives as inhibitors of p53 ubiquitination by HDM2

Michael P. Dickens^a^, Patricia Roxburgh^b^, Andreas Hock^b^, Mokdad Mezna^b^, Barrie Kellam^a^, Karen H. Vousden^b^ and Peter M. Fischera,[[1]](#footnote-2)*

^a^ School of Pharmacy & Centre for Biomolecular Sciences, University of Nottingham, University Park, Nottingham, NG7 2RD, UK

^b^ The Beatson Institute for Cancer Research, Garscube Estate, Switchback Road, Glasgow, G61 1BD, UK

**Table S1**

*Inhibition of in vitro ubiquitination of p53 by substituted 10-alkyl (****I****) and 10-aryldeazaflavins (****II****): summary of inactive compoundsa*

| Cmpd |  | | | | | | | | | Prescreen | IC_50_ (μM) |
| --- | --- | --- | --- | --- | --- | --- | --- | --- | --- | --- | --- |
|  | R^1^ | R^2^ | R^3^ | R^4^ | R^5^ | R^6^ | R^7^ | R^8^ | R^9^ |  |  |
| **31** (**I**) | NO_2_ | H | H | H | H | H | H | H | - | Inactive | - |
| **32** (**I**) | H | NO_2_ | H | H | H | H | H | H | - | Inactive | - |
| **33** (**I**) | H | H | NO_2_ | H | H | H | H | H | - | Inactive | - |
| **34** (**I**) | NO_2_ | H | H | H | F | H | H | H | - | Inactive | - |
| **35** (**I**) | H | NO_2_ | H | H | F | H | H | H | - | Inactive | - |
| **36** (**I**) | H | H | NO_2_ | H | F | H | H | H | - | Inactive | - |
| **37** (**I**) | NO_2_ | H | H | H | H | H | Cl | H | - | Inactive | - |
| **38** (**I**) | H | H | NO_2_ | H | H | H | Cl | H | - | Inactive | - |
| **39** (**I**) | H | H | H | H | H | H | H | H | - | Inactive | - |
| **40** (**I**) | H | H | H | H | H | H | Cl | H | - | Inactive | - |
| **41** (**I**) | H | H | H | H | F | H | H | H | - | Inactive | - |
| **42** (**I**) | H | H | H | H | H | F | H | H | - | Inactive | - |
| **43** (**I**) | H | H | H | H | H | H | F | H | - | Inactive | - |
| **44** (**I**) | H | H | H | H | Cl | H | H | H | - | Inactive | - |
| **45** (**I**) | H | H | H | H | H | Cl | H | H | - | Inactive | - |
| **46** (**I**) | H | H | H | H | F | H | Cl | H | - | Inactive | - |
| **47** (**I**) | H | H | H | H | Me | H | H | H | - | Inactive | - |
| **48** (**I**) | H | H | H | H | H | Me | H | H | - | Inactive | - |
| **49** (**I**) | H | H | H | H | H | H | Me | H | - | Inactive | - |
| **50** (**II**) | H | H | H | H | - | - | - | H | H | Inactive | - |
| **51** (**II**) | H | H | H | H | - | - | - | H | Me | Inactive | - |
| **52** (**II**) | H | H | H | H | - | - | - | H | Bn | Inactive | - |
| **53** (**I**) | H | H | H | H | H | H | H | Me | - | Inactive | - |
| **54** (**I**) | H | H | H | H | H | H | H | Et | - | Inactive | - |
| **55** (**I**) | CF_3_ | H | H | H | H | H | H | H | - | Inactive | - |
| **56** (**I**) | H | CF_3_ | H | H | H | H | H | H | - | Inactive | - |
| **57** (**I**) | H | H | CF_3_ | H | H | H | H | H | - | Inactive | - |
| **58** (**I**) | CF_3_ | H | H | H | F | H | H | H | - | Inactive | - |
| **59** (**I**) | H | CF_3_ | H | H | F | H | H | H | - | Inactive | - |
| **60** (**I**) | H | H | H | CF_3_ | Cl | H | H | H | - | Inactive | - |
| **61** (**I**) | H | H | H | CF_3_ | Me | H | H | H | - | Inactive | - |
| **62** (**I**) | H | H | H | CF_3_ | H | H | Cl | Me | - | Inactive | - |
| **63** (**I**) | H | H | H | CF_3_ | H | H | Cl | Et | - | Inactive | - |
| **64** (**II**) | H | H | H | CF_3_ | - | - | - | H | H | Inactive | - |
| **65** (**II**) | H | H | H | CF_3_ | - | - | - | H | Me | Inactive | - |
| **66** (**I**) | Cl | H | H | H | H | H | H | H | - | Inactive | - |
| **67** (**I**) | H | Cl | H | H | H | H | H | H | - | Inactive | - |
| **68** (**I**) | H | H | Cl | H | H | H | H | H | - | Inactive | - |

*^a^* For explanation of prescreen refer to main text.

**Table S1 (continued)**

*Inhibition of in vitro ubiquitination of p53 by substituted 10-alkyl (****I****) and 10-aryldeazaflavins (****II****): summary of inactive compoundsa*

| Cmpd |  | | | | | | | | | Prescreen | IC_50_ (μM) |
| --- | --- | --- | --- | --- | --- | --- | --- | --- | --- | --- | --- |
|  | R^1^ | R^2^ | R^3^ | R^4^ | R^5^ | R^6^ | R^7^ | R^8^ | R^9^ |  |  |
| **69** (**I**) | Cl | H | H | H | F | H | H | H | - | Inactive | - |
| **70** (**I**) | H | H | Cl | H | F | H | H | H | - | Inactive | - |
| **71** (**I**) | H | H | H | Cl | F | H | H | H | - | Inactive | - |
| **75** (**I**) | H | Cl | H | H | H | H | Cl | H | - | Inactive | - |
| **73** (**I**) | H | H | Cl | H | H | H | Cl | H | - | Inactive | - |
| **74** (**I**) | H | H | H | Cl | Cl | H | H | H | - | Inactive | - |
| **75** (**I**) | H | H | H | Cl | H | H | F | H | - | Inactive | - |
| **76** (**I**) | H | H | H | Cl | F | H | Cl | H | - | Inactive | - |
| **77** (**I**) | H | H | H | Cl | Me | H | H | H | - | Inactive | - |
| **78** (**I**) | H | H | H | Cl | H | Me | H | H | - | Inactive | - |
| **79** (**I**) | H | H | H | Cl | H | H | Me | H | - | Inactive | - |
| **80** (**I**) | Me | H | H | H | H | H | H | H | - | Inactive | - |
| **81** (**I**) | H | Me | H | H | H | H | H | H | - | Inactive | - |
| **82** (**I**) | H | H | Me | H | H | H | H | H | - | Inactive | - |
| **83** (**I**) | H | H | H | Me | H | H | H | H | - | Inactive | - |
| **84** (**I**) | Me | H | H | H | H | H | Cl | H | - | Inactive | - |
| **85** (**I**) | H | Me | H | H | H | H | Cl | H | - | Inactive | - |
| **86** (**I**) | H | H | H | Me | H | H | Cl | H | - | Inactive | - |
| **87** (**I**) | Me | H | H | H | F | H | H | H | - | Inactive | - |
| **88** (**I**) | H | Me | H | H | F | H | H | H | - | Inactive | - |
| **89** (**I**) | H | H | Me | H | F | H | H | H | - | Inactive | - |
| **90** (**I**) | H | H | H | Me | F | H | H | H | - | Inactive | - |
| **91** (**I**) | H | H | H | OH | H | H | H | H | - | Inactive | - |
| **92** (**I**) | H | H | H | OH | H | H | Cl | H | - | Inactive | - |
| **93** (**I**) | H | H | H | F | H | H | H | H | - | Inactive | - |
| **94** (**I**) | H | H | H | F | H | H | Cl | H | - | Inactive | - |
| **95** (**I**) | H | H | H | CN | H | Cl | H | H | - | Inactive | - |
| **96** (**I**) | H | H | H | CN | H | H | Cl | H | - | Inactive | - |

*^a^* For explanation of prescreen refer to main text.

**^1^H- and ^13^C-NMR spectra for compounds 10, 18, and 69**

**
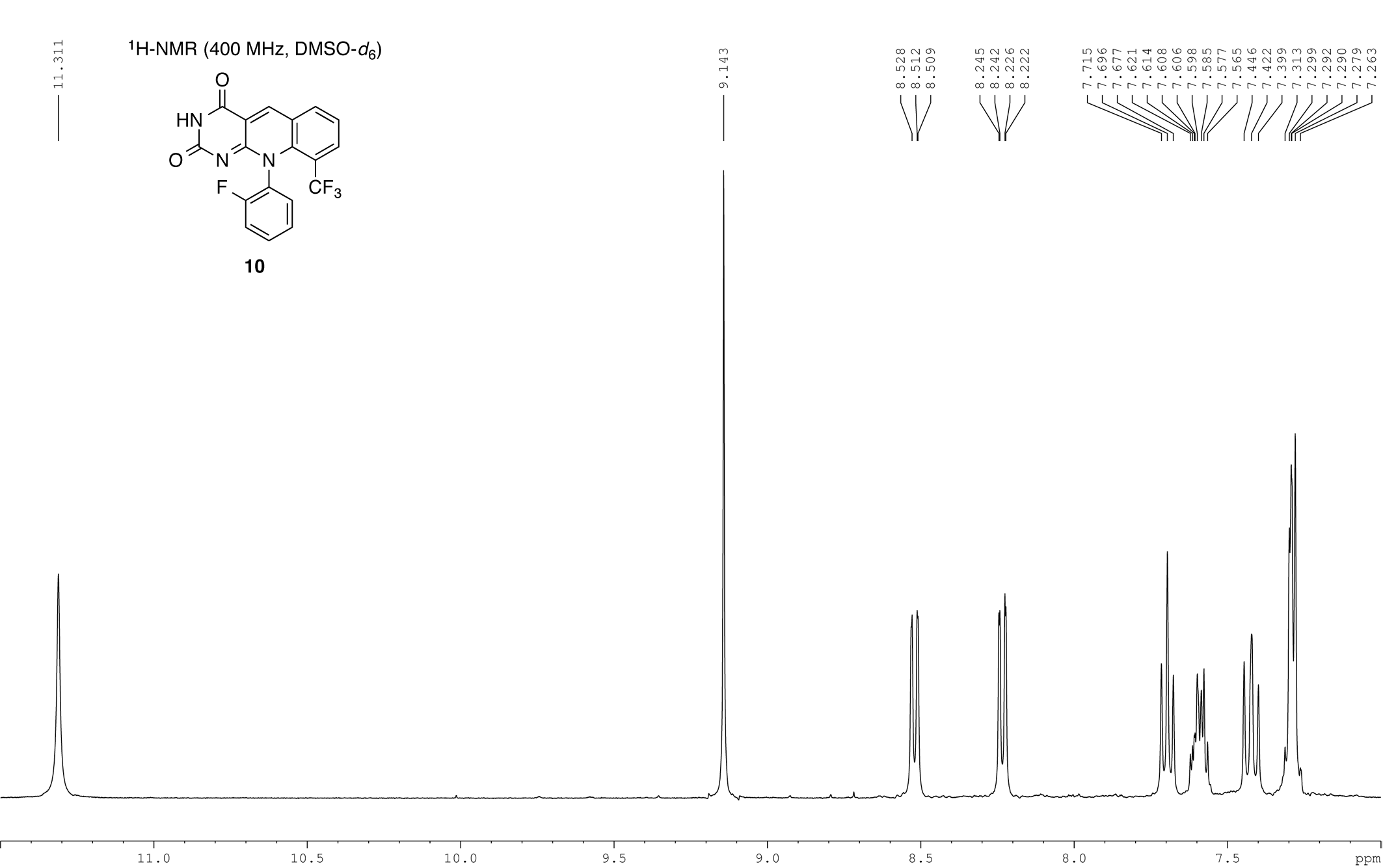
**

**
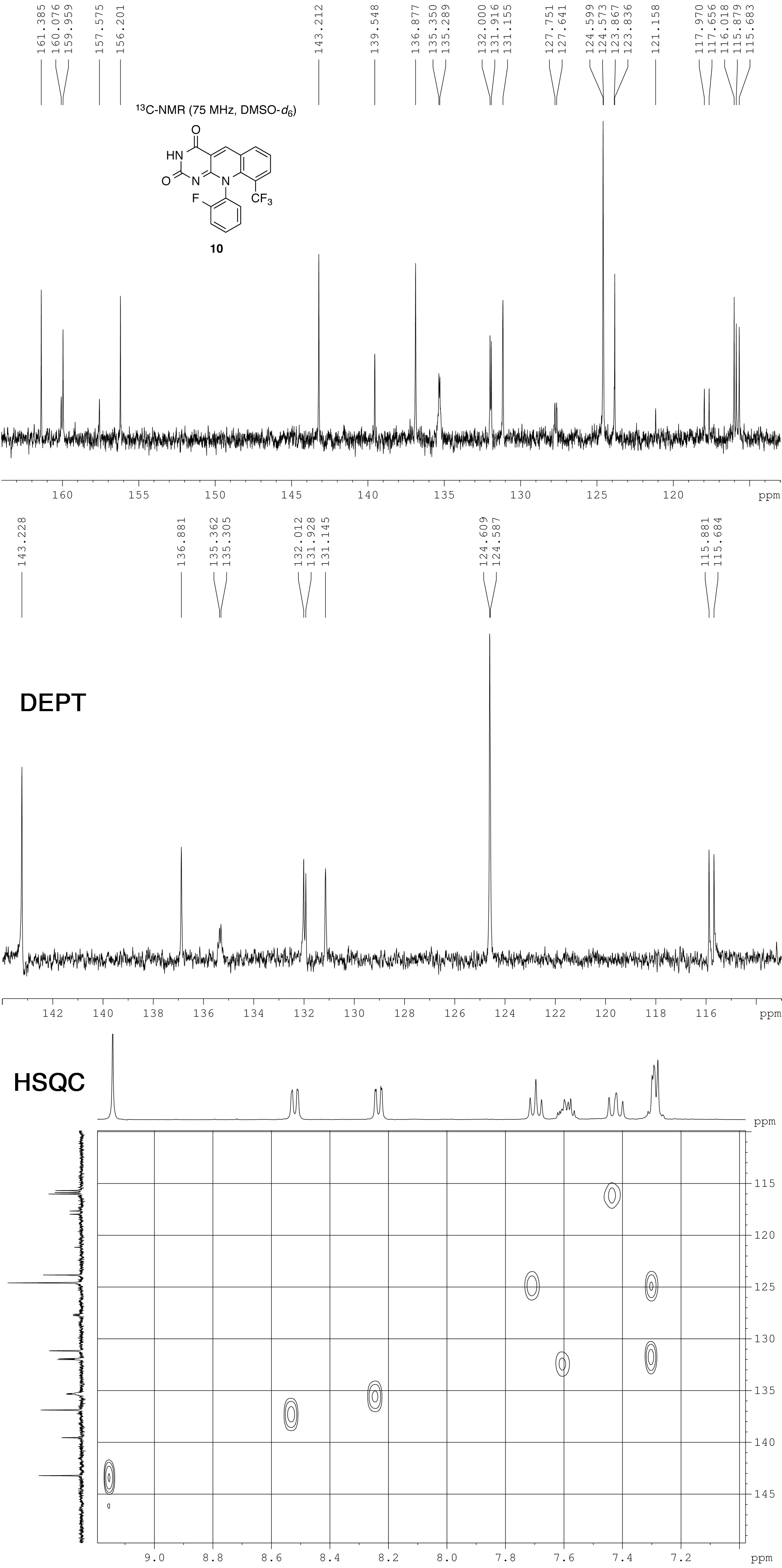
**

**
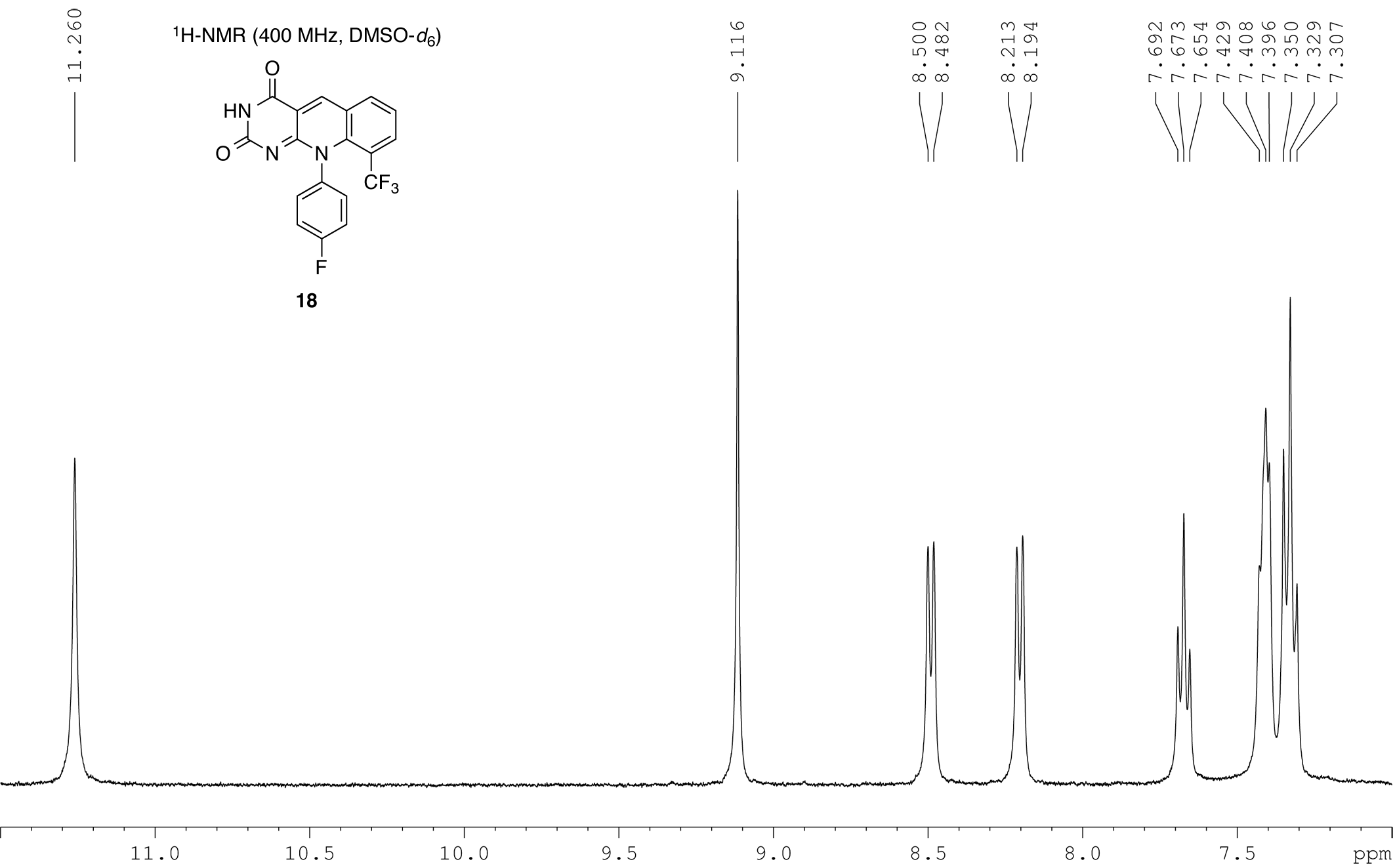
**

**
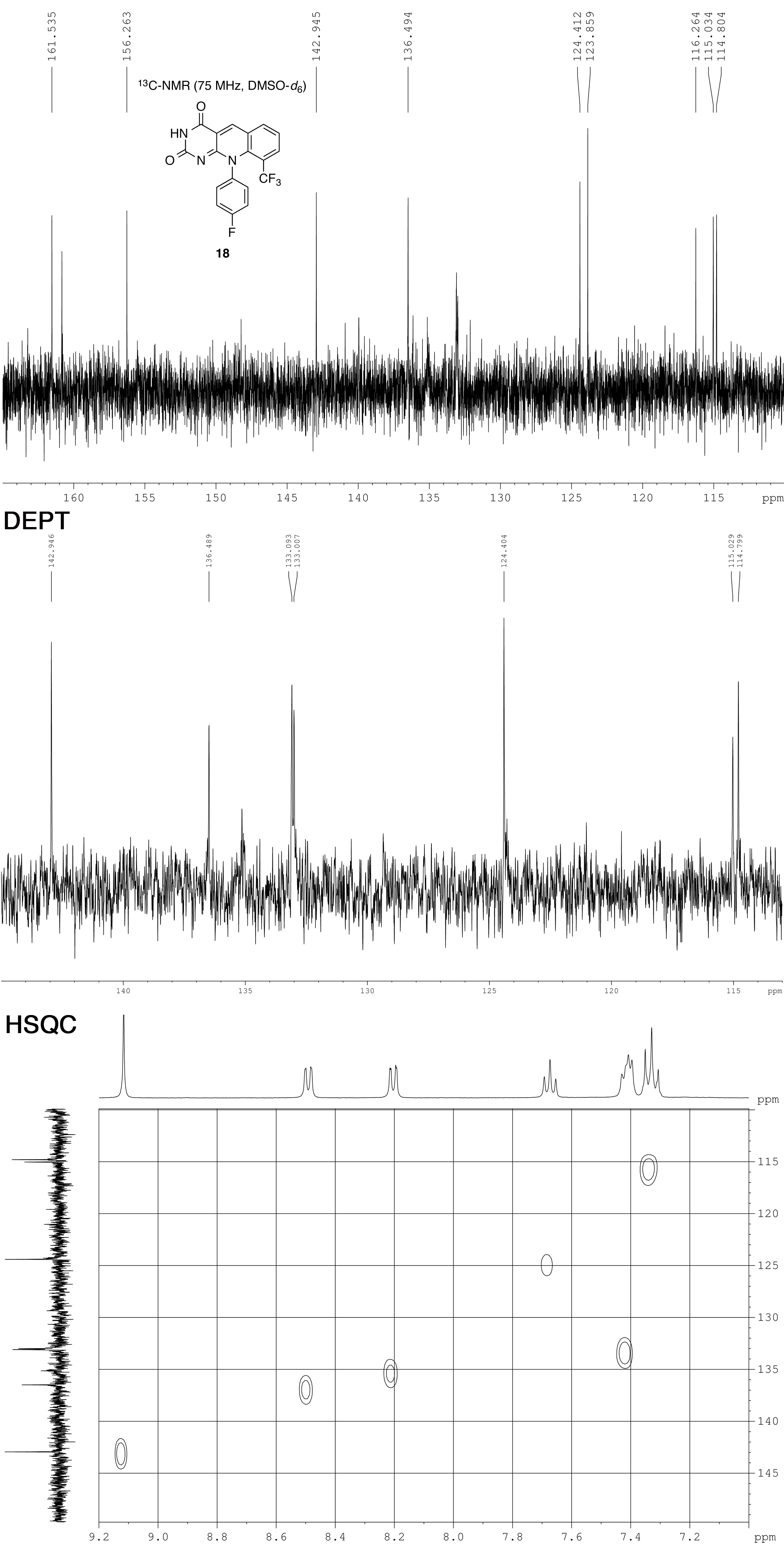
**

**
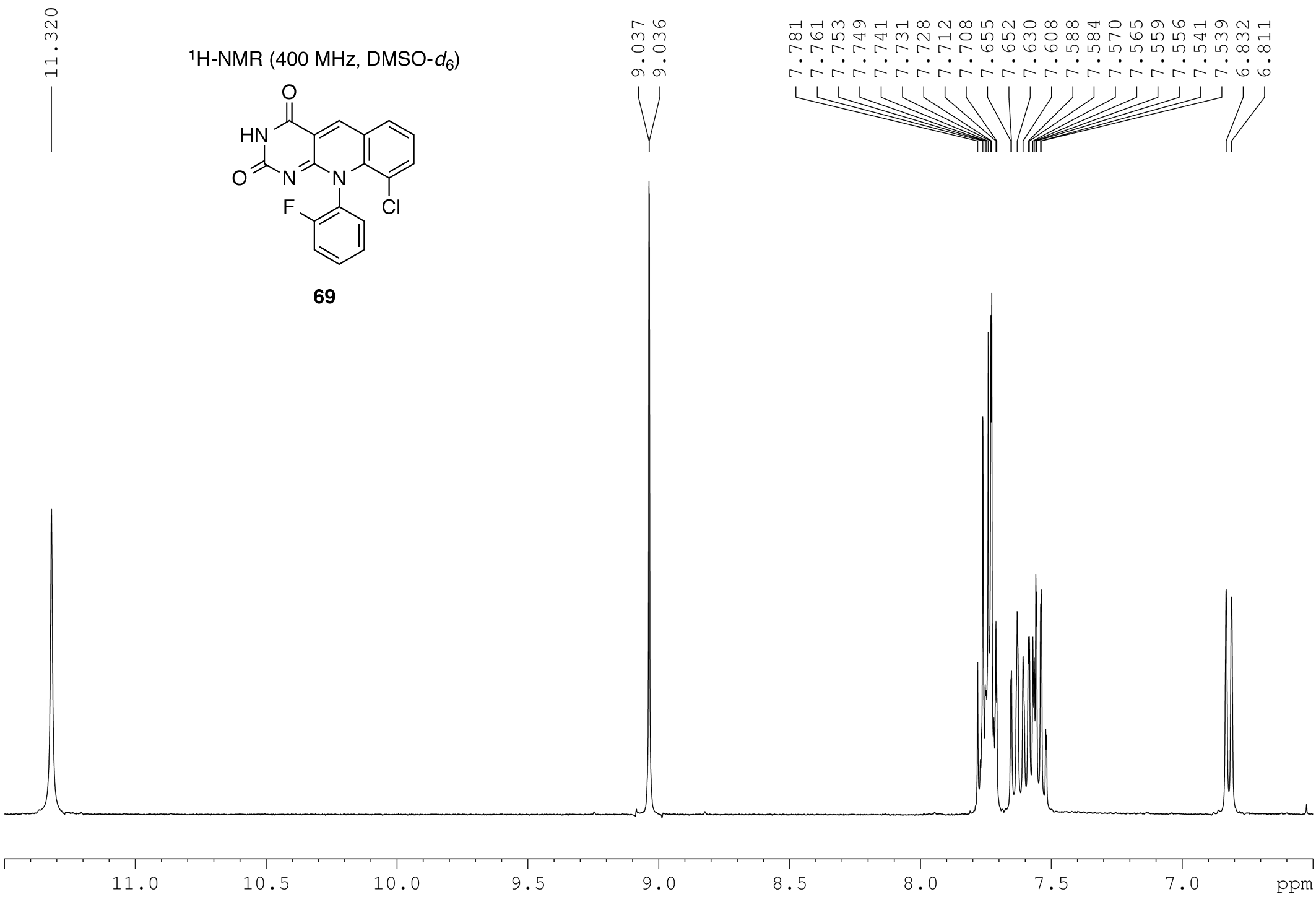
**

**
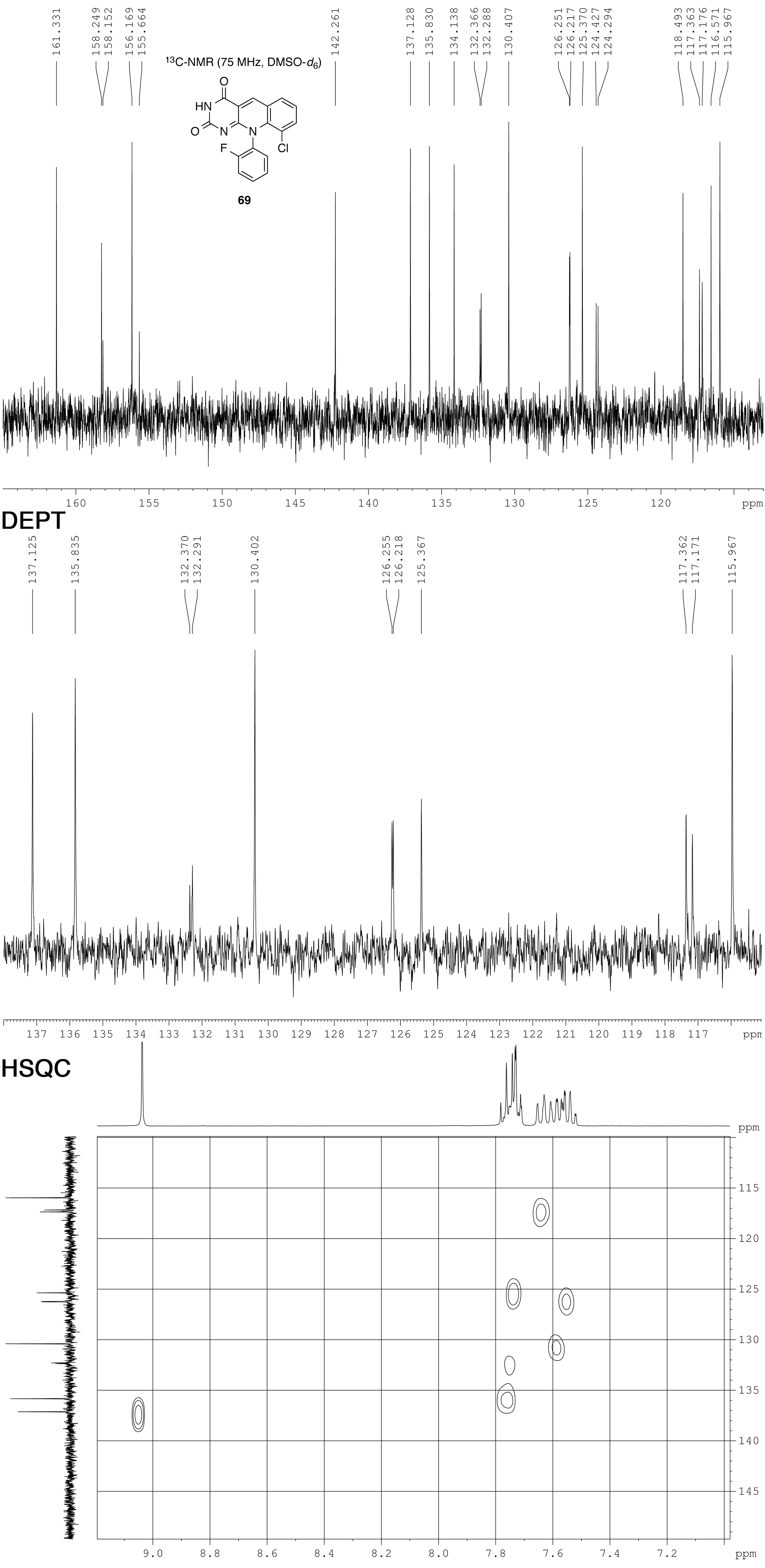
**

**Preparative and analytical data for compounds 31**–**96**

**6-Nitro-10-phenyl-2*H*,3*H*,4*H*,10*H*-pyrimido[4,5-*b*]quinoline-2,4-dione (31)**

Prepared using general method 5.7 from **3a** (R^5–7^ = H) and **4** (R^1^ = NO_2_, R^2–4^ = H, X = F). Yellow solid (66.5 mg, 67%). Mp: 348–349 ^o^C (dec.); IR (KBr): 3,434 (NH), 1,707 (C=O), 1,677 (C=O), 1,615 (C=C), 1,551 (NO_2_), 1,296 (NO_2_) cm^-1^; ^1^H-NMR: *δ* 7.08 (1H, d, *J* = 8.3 Hz, C9-H), 7.46 (2H, d, *J* = 7.0 Hz, Ph 2-H), 7.63-7.75 (3H, m, Ph-H), 7.87 (1H, t, *J* = 8.3 Hz, C8-H) 8.18 (1H, d, *J* = 8.3 Hz, C7-H), 9.20 (1H, s, C5-H), 11.30 (1H, s, N_3_-H); ^13^C-NMR: *δ* 118.35 (Cq), 121.55 (CH), 123.27 (CH), 128.80 (CH), 130.01 (Cq), 130.18 (CH), 130.92 (CH), 134.36 (CH), 136.16 (CH), 137.89 (Cq), 140.29 (Cq), 142.95 (Cq), 156.74 (Cq), 159.96 (Cq), 161.87 (Cq); anal. RP-HPLC: *t*_R_ 2.43 min (98.3%, A), 7.55 min (97.9%, B); HRMS (ESI^+^): calcd for C_17_H_11_N_4_O_4_ [M+H]^+^ 335.0780, found 335.0783.

**7-Nitro-10-phenyl-2*H*,3*H*,4*H*,10*H*-pyrimido[4,5-*b*]quinoline-2,4-dione (32)**

Prepared using general method 5.7 from **3a** (R^5–7^ = H) and **4** (R^1,3,4^ = H, R^2^ = NO_2_, X = F). Yellow solid (66.1 mg, 66%). Mp: > 350 ^o^C (lit.[^1^](#_ENREF_1) > 360 ^o^C); IR (KBr): 3,447 (NH), 1,720 (C=O), 1,659 (C=O), 1,613 (C=C), 1,521 (NO_2_), 1,357 (NO_2_) cm-1; ^1^H-NMR: *δ* 6.76 (1H, d, *J* = 9.4 Hz, C9-H), 7.36 (2H, d, *J* = 8.0 Hz, Ph 2-H), 7.53-7.65 (3H, m, Ph-H), 8.34 (1H, dd, ^4^*J* =2.6 Hz, ^3^*J* = 9.4 Hz, C8-H), 9.13 (1H, d, ^4^*J* = 2.6 Hz, C6-H), 9.20 (1H, s, C5-H), 11.19 (1H, s, N3-H); ^13^C-NMR: *δ* 118.05 (CH), 119.00 (Cq), 120.92 (Cq), 127.68 (CH), 128.75 (CH), 128.85 (CH), 130.19 (CH), 130.92 (CH), 137.75 (Cq), 142.62 (CH), 143.37 (Cq), 145.60 (Cq), 156.78 (Cq), 159.98 (Cq), 161.90 (Cq); anal. RP-HPLC: *t*_R_ 0.92 min (99.1%, A), 4.37 min (98.6%, B); HRMS (ESI^+^): calcd for C_17_H_11_N_4_O_4_ [M+H]^+^ 335.0780, found 335.0776.

**8-Nitro-10-phenyl-2*H*,3*H*,4*H*,10*H*-pyrimido[4,5-*b*]quinoline-2,4-dione (33)**

Prepared using general method 5.7 from **3a** (R^5–7^ = H) and **4** (R^1,2,4^ = H, R^3^ = NO_2_, X = Cl). Yellow solid (42.6 mg, 71%). Mp: of 342–343 ^o^C; IR (KBr): 3,430 (NH), 1,703 (C=O), 1,667 (C=O), 1,607 (C=C), 1,539 (NO_2_), 1317 (NO_2_) cm^-1^; ^1^H-NMR: *δ* 7.37 (1H, d, ^4^*J* = 1.9 Hz, C9-H), 7.49 (2H, d, *J* = 7.0 Hz, Ph-H), 7.66-7.78 (3H, m, Ph-H), 8.23 (1H, dd, ^4^*J* = 1.9 Hz, ^3^*J* = 8.6 Hz, C7-H), 8.49 (1H, d, *J* = 8.6 Hz, C6-H), 9.21 (1H, s, C5-H), 11.27 (1H, s, N3-H); ^13^C-NMR: *δ* 112.36 (CH), 118.55 (CH), 119.12 (Cq), 125.20 (Cq), 128.89 (CH), 130.35 (CH), 130.97 (CH), 133.64 (CH), 137.45 (Cq), 141.45 (CH), 142.11 (Cq), 150.45 (Cq), 156.66 (Cq), 159.61 (Cq), 161.88; anal. RP-HPLC: *t*_R_ 1.28 min (99.8%, A), 10.47 min (100%, B); HRMS (ESI^+^): calcd for C_17_H_11_N_4_O_4_ [M+H]^+^ 335.0780, found 335.0764.

**10-(2-Fluorophenyl)-6-nitro-2*H*,3*H*,4*H*,10*H*-pyrimido[4,5-*b*] quinoline-2,4-dione (34)**

Prepared using general method 5.7 from **3a** (R^5^ = F, R^6,7^ = H) and **4** (R^1^ = NO_2_, R^2–4^ = H, X = F). Yellow solid (135.3 mg, 10%). Mp: 306–308 ^o^C; IR (KBr): 3,442 (NH), 1,707 (C=O), 1,678 (C=O), 1,620 (C=C), 1,518 (NO_2_), 1,289 (NO_2_) cm^-1^; ^1^H-NMR: *δ* 7.22 (1H, d, *J* = 8.2 Hz, C9-H), 7.52-7.68 (3H, m, Ph-H), 7.72-7.80 (1H, m, Ph-H), 7.92 (1H, t, *J* = 8.2 Hz, C8-H), 8.22 (1H, d, *J* = 8.2 Hz, C7-H), 9.23 (1H, s, C5-H), 11.40 (1H, s, N3-H); ^13^C-NMR: *δ* 114.01 (Cq), 117.85 (CH, d, *J* = 19.35 Hz), 118.09 (Cq), 122.05 (CH), 122.53 (CH), 124.67 (Cq, d, *J* = 13.64 Hz), 126.83 (CH, d, *J* = 3.46 Hz), 130.91 (CH), 132.99 (CH, d, *J* = 8.01 Hz), 135.05 (CH), 136.91 (CH), 142.02 (Cq), 148.21 (Cq), 156.55 (Cq), 157.49 (Cq, d, *J* = 250.29 Hz), 158.38 (Cq), 161.63 (Cq); anal. RP-HPLC: *t*_R_ 1.20 min (97.8%, A), 9.28 min (98.7%, B); HRMS (ESI^+^): calcd for C_17_H_10_FN_4_O_4_ [M+H]^+^ 353.0686, found 353.0694.

**10-(2-Fluorophenyl)-7-nitro-2*H*,3*H*,4*H*,10*H*-pyrimido[4,5-*b*] quinoline-2,4-dione (35)**

Prepared using general method 5.7 from **3a** (R^5^ = F, R^6,7^ = H) and **4** (R^1,3,4^ = H, R^2^ = NO_2_, X = F). Yellow solid (88.9 mg, 89%). Mp: 326–327 ^o^C; IR (KBr): 3,413 (NH), 1,711 (C=O), 1,685 (C=O), 1,616 (C=C), 1,488 (NO_2_), 1,309 (NO_2_) cm^-1^; ^1^H-NMR: *δ* 6.98 (1H, d, *J* = 9.4 Hz, C9-H), 7.53-7.69 (3H, m, Ph-H), 7.73-7.80 (1H, m, Ph-H), 8.48 (1H, dd, _4_*J*= 2.3 Hz, ^3^*J* = 9.4 Hz, C8-H), 9.25 (1H, d, ^4^*J* = 2.3 Hz, C6-H), 9.33 (1H, s, C5-H), 11.38 (1H, s, N3-H); ^13^C-NMR: *δ* 117.82 (CH, d, *J* = 19.02 Hz), 117.86 (Cq), 118.26 (CH), 120.99 (Cq), 124.54 (Cq, d, *J* = 13.34 Hz), 126.81 (CH, d, *J* = 3.56 Hz), 127.98 (CH), 129.44 (CH), 130.85 (CH), 132.99 (CH, d, *J* = 7.85 Hz), 143.07 (CH), 143.79 (Cq), 144.72 (Cq), 156.65 (Cq), 157.56 (Cq, d, *J* = 242.62 Hz), 159.58 (Cq), 161.65 (Cq); anal. RP-HPLC: *t*_R_ 1.49 min (100%, A), 4.60 min (97.2%, B); HRMS (ESI^+^): calcd for C_17_H_10_FN_4_O_4_ [M+H]^+^ 353.0686, found 353.0671.

**10-(2-Fluorophenyl)-8-nitro-2*H*,3*H*,4*H*,10*H*-pyrimido[4,5-*b*] quinoline-2,4-dione (36)**

Prepared using general method 5.7 from **3a** (R^5^ = F, R^6,7^ = H) and **4** (R^1,2,4^ = H, R^3^ = NO_2_, X = Cl). Yellow solid (23.6 mg, 37%). M.p. 308–310 ^o^C; IR (KBr): 3,420 (NH), 1,711 (C=O), 1,670 (C=O), 1,628 (C=C), 1,535 (NO_2_), 1,342 (NO_2_) cm^-1^; ^1^H-NMR: *δ* 7.43 (1H, s, C9-H), 7.56-7.72 (3H, m, Ph-H), 7.75-7.83 (1H, m, Ph-H), 8.27 (1H, dd, ^4^*J* = 2.0 Hz, ^3^*J* = 8.6 Hz, C7-H), 8.52 (1H, d, *J* = 8.6 Hz, C6-H), 9.24 (1H, s, C5-H), 11.36 (1H, s, N3-H); ^13^C-NMR: *δ* 111.51 (CH), 117.96 (CH, d, *J* = 17.58 Hz), 118.91 (Cq), 119.14 (CH), 124.23 (Cq, d, *J* = 13.18 Hz), 125.25 (Cq), 126.86 (CH, d, *J* = 2.93 Hz), 130.96 (CH), 133.17 (CH, d, *J* = 7.91 Hz), 134.05 (CH), 141.20 (Cq), 141.78 (CH), 150.89 (Cq), 156.56 (Cq), 157.61 (Cq, d, *J* = 250.48 Hz), 159.26 (Cq), 161.64 (Cq); anal. RP-HPLC: *t*_R_ 1.37 min (100%, A), 4.47 min (98.7%, B); HRMS (ESI^+^): calcd for C_17_H_10_FN_4_O_4_ [M+H]^+^ 353.0686, found 353.0678.

**10-(4-Chlorophenyl)-6-nitro-2*H*,3*H*,4*H*,10*H*-pyrimido[4,5-*b*] quinoline-2,4-dione (37)**

Prepared using general method 5.7 from **3a** (R^5,6^ = H, R^7^ = Cl) and **4** (R^1^ = NO_2_, R^2–4^ = H, X = F). Yellow solid (188.9 mg, 20%). Mp: 258–260 ^o^C; IR (KBr): 3,400 (NH), 1,715 (C=O), 1,649 (C=O), 1,614 (C=C), 1,531 (NO_2_), 1,347 (NO_2_) cm^-1^; ^1^H-NMR: *δ* 7.18 (1H, d, *J* = 8.2 Hz, C9-H), 7.51 (2H, d, AA’BB’ system, *J* = 8.5 Hz, Ph2-H), 7.79 (2H, d, AA’BB’ system, *J* = 8.5 Hz, Ph 3-H), 7.87 (1H, t, *J* = 8.2 Hz, C8-H), 8.19 (1H, d, *J* = 8.2 Hz, C7-H), 9.19 (1H, s, C5-H), 11.32 (1H, s, N3-H); ^13^C-NMR: *δ* 113.86 (Cq), 118.27 (Cq), 121.64 (CH), 123.37 (CH), 130.87 (CH), 131.04 (CH), 134.43 (CH), 134.89 (Cq), 136.32 (CH), 136.68 (Cq), 142.77 (Cq), 148.05 (Cq), 156.58 (Cq), 158.77 (Cq), 161.82 (Cq); anal. RP-HPLC: *t*_R_ 1.75 min (98.7%, A), 5.22 min (99.0%, B); HRMS (ESI^+^): calcd for C_17_H_10_ClN_4_O_4_ [M+H]^+^ 369.0390, found 369.0394.

**10-(4-Chlorophenyl)-8-nitro-2*H*,3*H*,4*H*,10*H*-pyrimido[4,5-*b*] quinoline-2,4-dione (38)**

Prepared using general method 5.7 from **3a** (R^5,6^ = H, R^7^ = Cl) and **4** (R^1,2,4^ = H, R^3^ = NO_2_, X = Cl). Yellow solid (33.9 mg, 51%). M.p: 328–329 ^o^C (dec); IR (KBr): 3,429 (NH), 1,711 (C=O), 1,654 (C=O), 1,610 (C=C), 1,521 (NO_2_), 1,310 (NO_2_) cm^-1^; ^1^H-NMR: *δ* 7.40 (1H, d, ^4^*J* = 1.9 Hz, C9-H), 7.55 (2H, d, AA’BB’ system, *J* = 8.6 Hz, Ph 2-H), 7.83 (2H, d, AA’BB’ system, *J* =8.6 Hz, Ph 3-H), 8.25 (1H, dd, ^4^*J* = 1.9 Hz, ^3^*J* = 8.6 Hz, C7-H), 8.49 (1H, d, *J* = 8.6 Hz, C6-H), 9.22 (1H, s, C5-H), 11.31 (1H, s, N3-H); ^13^C-NMR: *δ* 112.21 (CH), 118.66 (CH), 119.08 (Cq), 125.24 (Cq), 130.99 (CH), 131.09 (CH), 133.72 (CH), 135.00 (Cq), 136.22 (Cq), 141.36 (CH), 141.89 (Cq), 150.55 (Cq), 156.56 (Cq), 159.69 (Cq), 161.82 (Cq); anal. RP-HPLC: *t*_R_ 2.13 min (97.4%, A), 5.42 min (100%, B); HRMS (ESI^+^): calcd for C_17_H_10_ClN_4_O_4_ [M+H]^+^ 369.0390, found 369.0348.

**10-Phenyl-2*H*,3*H*,4*H*,10*H*-pyrimido[4,5-*b*]quinoline-2,4-di-one (39)**

Prepared using general method 5.7 from **3a** (R^5–7^ = H) and **4** (R^1–4^ = H, X = F). Yellow solid (58.7 mg, 59% yield); ^1^H-NMR: *δ* 6.71 (1H, d, *J* = 8.5 Hz, C9-H), 7.43 (2H, d, *J* = 7.1 Hz, Ph 2-H), 7.50 (1H, t, *J* =8.5 Hz, C8-H), 7.60-7.78 (4H, m, C7-H & Ph-H), 8.23 (1H, d, *J* = 8.5 Hz, C6-H), 9.12 (1H, s, C5-H), 11.06 (1H, s, N3-H); HRMS (ESI^+^): calcd for C_17_H_12_N_3_O_2_ [M+H]^+^ 290.0930, found 290.0931.

**10-(4-Chlorophenyl)-2*H*,3*H*,4*H*,10*H*-pyrimido[4,5-*b*] quinol-ine-2,4-dione (40)**

Prepared using general method 5.7 from **3a** (R^5,6^ = H, R^7^ = Cl) and **4** (R^1–4^ = H, X = F). Yellow solid (55.9 mg, 56%). Mp: > 350 ^o^C (lit.[^2^](#_ENREF_2) > 300 ^o^C); IR (KBr): 3,421 (NH), 1,701 (C=O), 1,665 (C=O), 1,609 (C=C) cm^-1^; ^1^H-NMR: *δ* 6.79 (1H, d, *J* = 8.8 Hz, C9-H), 7.44-7.55 (3H, m), 7.69-7.82 (3H, m), 8.23 (1H, d, *J* = 8.8 Hz, C6-H), 9.13 (1H, s, C5-H), 11.01 (1H, br. s, N3-H); ^13^C-NMR: *δ* 116.08 (Cq), 117.56 (CH), 121.47 (Cq), 124.95 (CH), 130.81 (CH), 130.98 (CH), 131.89 (CH), 134.51 (Cq), 135.61 (CH), 136.98 (Cq), 142.05 (Cq), 143.00 (CH), 156.82 (Cq), 159.20 (Cq), 162.34 (Cq); anal. RP-HPLC: *t*_R_ 1.69 min (100%, A), 4.59 min (98.5%, B); HRMS (ESI^+^): calcd for C_17_H_11_ClN_3_O_2_ [M+H]^+^ 324.0540, found 324.0551.

**10-(2-Fluorophenyl)-2*H*,3*H*,4*H*,10*H*-pyrimido[4,5-*b*] quinol-ine-2,4-dione (41)**

Prepared using general method 5.7 from **3a** (R^5^ = F, R^6,7^ = H) and **4** (R^1–4^ = H, X = F). Yellow solid (69.9 mg, 70%). Mp: 308–310 ^o^C; IR (KBr): 3,444 (NH), 1,705 (C=O), 1,654 (C=O), 1,611 (C=C) cm^-1^; ^1^H-NMR: *δ* 6.82 (1H, d, *J* = 8.4 Hz, C9-H), 7.50-7.65 (4H, m), 7.69-781 (2H, m), 8.25 (1H, d, *J* = 8.4 Hz, C6-H), 9.16 (1H, s, C5-H), 11.16 (1H, s, N3-H); ^13^C-NMR: *δ* 115.92 (Cq), 116.74 (CH), 117.67 (CH, d, *J* = 19.20 Hz), 121.42 (Cq), 124.98 (Cq, d, *J* = 13.25 Hz), 125.33 (CH), 126.62 (CH, d, *J* = 3.75 Hz), 131.03 (CH), 132.21 (CH), 132.57 (CH, d, *J* = 10.05 Hz), 136.11 (CH), 141.38 (Cq), 143.49 (CH), 157.47 (Cq, d, *J* = 250.00 Hz), 156.83 (Cq), 158.90 (Cq), 162.16 (Cq); anal. RP-HPLC: *t*_R_ 1.11 min (98.6%, A), 3.72 min (97.6%, B); HRMS (ESI^+^): calcd for C_17_H_11_FN_3_O_2_ [M+H]^+^ 308.0835, found 308.0846.

**10-(3-Fluorophenyl)-2*H*,3*H*,4*H*,10*H*-pyrimido[4,5-*b*] quinol-ine-2,4-dione (42)**

Prepared using general method 5.7 from **3a** (R^5,7^ = H, R^6^ = F) and **4** (R^1–4^ = H, X = F). Yellow solid (20.2 mg, 20%). Mp: 348–350 ^o^C; IR (KBr): 3,501 (NH), 1,696 (C=O), 1,608 (C=O), 1,561 (C=C) cm^-1^; ^1^H-NMR: *δ* 6.78 (1H, d, *J* = 8.3 Hz, C9-H), 7.29-7.33 (1H, m), 7.43-7.54 (3H, m), 7.71-7.78 (2H, m), 8.23 (1H, dd, ^4^*J* = 1.3 Hz, ^3^*J* = 8.3 Hz, C6-H), 9.13 (1H, s, C5-H), 11.09 (1H, s, N3-H); ^13^C-NMR: *δ* 116.05 (Cq), 166.66 (CH, d, *J* = 24.05 Hz), 117.01 (CH, d, *J* = 20.76 Hz), 117.48 (CH), 121.43 (Cq), 124.97 (CH), 125.34 (CH, d, *J* = 2.95 Hz), 131.87 (CH), 132.39 (CH, d, *J* = 9.06 Hz), 135.65 (CH), 139.46 (Cq, d, *J* = 10.25 Hz), 141.95 (Cq), 143.04 (CH), 156.81 (Cq), 159.10 (Cq), 162.29 (Cq), 163.40 (Cq, d, *J* = 244.81 Hz); anal. RP-HPLC: *t*_R_ 0.99 min (98.3%, A), 9.22 min (100%, B); HRMS (ESI^+^): calcd for C_17_H_11_FN_3_O_2_ [M+H]^+^ 308.0835, found 308.0850.

**10-(4-Fluorophenyl)-2*H*,3*H*,4*H*,10*H*-pyrimido[4,5-*b*] quinol-ine-2,4-dione (43)**

Prepared using general method 5.7 from **3a** (R^5,6^ = H, R^7^ = F) and **4** (R^1–4^ = H, X = F). Yellow solid (45.8 mg, 46%). Mp: 344–346 ^o^C (dec); IR (KBr): 3,417 (NH), 1,715 (C=O), 1,655 (C=O), 1,614 (C=C) cm^-1^; ^1^H-NMR: *δ* 6.77 (1H, d, *J* = 8.2 Hz, C9-H), 7.46-7.57 (5H, m, C7-H & Ph-H), 7.74 (1H, ddd, ^4^*J* = 1.3 Hz, ^3^*J* = 8.2 Hz, C8-H), 8.23 (1H, dd, ^4^*J* = 1.3 Hz, ^3^*J* = 8.2 Hz, C6-H), 9.12 (1H, s, C5-H), 10.86 (1H, s, N3-H); ^13^C-NMR: *δ* 116.09 (Cq), 117.55 (CH, d, *J* = 4.51 Hz), 117.75 (CH), 121.47 (Cq), 124.90 (CH), 131.21 (CH, d, *J* = 8.47 Hz), 131.86 (CH), 134.28 (Cq, d, *J* = 2.94 Hz), 135.58 (CH), 142.35 (Cq), 142.97 (CH), 156.85 (Cq), 159.33 (Cq), 162.36 (Cq), 162.85 (Cq, d, *J* = 247.81 Hz); anal. RP-HPLC: *t*_R_ 0.99 min (99.6%, A), 9.15 min (100%, B); HRMS (ESI^+^): calcd for C_17_H_11_FN_3_O_2_ [M+H]^+^ 308.0835, found 308.0826.

**10-(2-Chlorophenyl)-2*H*,3*H*,4*H*,10*H*-pyrimido[4,5-*b*] quinol-ine-2,4-dione (44)**

Prepared using general method 5.7 from **3a** (R^5^ = Cl, R^6,7^ = H) and **4** (R^1–4^ = H, X = F). Yellow solid (70.2 mg, 70%). Mp: 347–348 ^o^C (dec); IR (KBr): 3,421 (NH), 1,702 (C=O), 1,676 (C=O), 1,614 (C=C) cm^-1^; ^1^H-NMR: *δ* 6.69 (1H, d, *J* = 8.0 Hz, C9-H), 7.54 (1H, t, *J* = 8.0 Hz, C7-H), 7.62-7.72 (3H, m, Ph-H), 7.71 (1H, ddd, ^4^*J* = 1.4 Hz, ^3^*J*= 8.0 Hz, C8-H), 7.83-7.87 (1H, m, Ph-H), 8.26 (1H, dd, ^4^*J* = 1.4 Hz, ^3^*J* = 8.0 Hz, C6-H), 9.17 (1H, s, C5-H), 11.16 (1H, s, N3-H); ^13^C-NMR: *δ* 115.87 (Cq), 116.68 (CH), 121.40 (Cq), 125.31 (CH), 129.77 (CH), 131.06 (CH), 131.27 (CH), 131.61 (Cq), 131.96 (CH), 132.21 (CH), 135.18 (Cq), 136.11 (CH), 141.07 (Cq), 143.54 (CH), 156.90 (Cq), 158.59 (Cq), 162.21 (Cq); anal. RP-HPLC: *t*_R_ 1.17 min (98.6%, A), 11.80 min (97.2%, B); HRMS (ESI^+^): calcd for C_17_H_11_ClN_3_O_2_ [M+H]^+^ 324.0540, found 324.0547.

**10-(3-Chlorophenyl)-2*H*,3*H*,4*H*,10*H*-pyrimido[4,5-*b*] quinol-ine-2,4-dione (45)**

Prepared using general method 5.7 from **3a** (R^5,7^ = H, R^6^ = Cl) and **4** (R^1–4^ = H, X = F). Yellow solid (54.6 mg, 55%). Mp: 346–347 ^o^C (dec); IR (KBr): 3,422 (NH), 1,703 (C=O), 1,659 (C=O), 1,614 (C=C) cm^-1^; ^1^H-NMR: *δ* 6.77 (1H, d, *J* = 7.9 Hz, C9-H), 7.43-7.47 (1H, m, Ph-H), 7.51 (1H, t, *J* = 7.9 Hz, C7-H), 7.66 (1H, s, Ph 2-H), 7.71-7.78 (3H, m, C8-H & Ph-H), 8.23 (1H, d, *J* = 7.9 Hz, C6-H), 9.12 (1H, s, C5-H), 11.10 (1H, s, N3-H); ^13^C-NMR: *δ* 116.03 (Cq), 117.50 (CH), 121.43 (Cq), 124.99 (CH), 127.94 (CH), 129.13 (CH), 130.04 (CH), 131.89 (CH), 132.33 (CH), 134.74 (Cq), 135.67 (CH), 139.38 (Cq), 141.93 (Cq), 143.08 (CH), 156.83 (Cq), 159.13 (Cq), 162.30 (Cq); anal. RP-HPLC: *t*_R_ 1.35 min (98.9%, A), 8.02 min (97.5%, B); HRMS (ESI^+^): calcd for C_17_H_11_ClN_3_O_2_ [M+H]^+^ 324.0540, found 324.0511.

**10-(2-Methylphenyl)-2*H*,3*H*,4*H*,10*H*-pyrimido[4,5-*b*] quinol-ine-2,4-dione (47)**

Prepared using general method 5.7 from **3a** (R^5^ = Me, R^6,7^ = H) and **4** (R^1–4^ = H, X = F). Yellow solid (45.6 mg, 46%). Mp: 294–296 ^o^C; IR (KBr): 3,425 (NH), 1,703 (C=O), 1,654 (C=O), 1,613 (C=C) cm^-1^; ^1^H-NMR: *δ* 2.42 (3H, s, Me), 6.73 (1H, d, *J* = 8.0 Hz, C9-H), 7.19-7.25 (2H, m, Ph-H), 7.44 (1H, m, Ph-H), 7.49 (1H, t, *J* = 7.4Hz, Ph-H), 7.57 (1H, t, *J* = 8.0 Hz, C7-H), 7.73 (1H, ddd, ^4^*J* = 1.4 Hz, ^3^*J* = 8.0 Hz, C8-H), 8.21 (1H, dd, ^4^*J* = 1.4 Hz, ^3^*J* = 8.0 Hz, C6-H), 9.11 (1H, s, C5-H), 11.06 (1H, s, N3-H); ^13^C-NMR: *δ* 21.33 (CH_3_), 116.05 (Cq), 117.66 (CH), 121.39 (Cq), 124.85 (CH), 125.80 (CH), 129.06 (CH), 130.45 (CH), 130.48 (CH), 131.78 (CH), 135.48 (CH), 138.06 (Cq), 140.39 (Cq), 142.22 (Cq), 142.83 (CH), 156.91 (Cq), 159.05 (Cq), 162,37 (Cq); anal. RP-HPLC: *t*_R_ 1.27 min (100%, A), 14.65 min (98.9%, B); HRMS (ESI^+^): calcd for C_18_H_14_N_3_O_2_ [M+H]^+^ 304.1086, found 304.1063.

**10-(3-Methylphenyl)-2*H*,3*H*,4*H*,10*H*-pyrimido[4,5-*b*] quinol-ine-2,4-dione (48)**

Prepared using general method 5.7 from **3a** (R^5,7^ = H, R^6^ = Me) and **4** (R^1–4^ = H, X = F). Yellow solid (20.8 mg, 21%). Mp: 328–329 ^o^C (dec); IR (KBr): 3,413 (NH), 1,706 (C=O), 1655 (C=O), 1,607 (C=C) cm^-1^; ^1^H-NMR: *δ* 1.89 (3H, s, Me), 6.64 (1H, d, *J* = 7.7 Hz, C9-H), 7.34-7.38 (1H, m, Ph-H), 7.47-7.58 (4H, m, C7-H & Ph-H), 7.74 (1H, ddd, ^4^*J* = 1.4 Hz, ^3^*J* = 7.7 Hz, C8-H), 8.25 (1H, dd, ^4^*J* = 1.4 Hz, ^3^*J* = 7.7 Hz, C6-H), 9.14 (1H, s, C5-H), 11.08 (1H, s, N3-H); ^13^C-NMR: *δ* 17.28 (CH_3_), 116.19 (Cq), 116.88 (CH), 121.54 (Cq), 125.08 (CH), 128.38 (CH), 128.70 (CH), 130.09 (CH), 131.98 (CH), 132.04 (CH),135.55 (Cq), 135.84 (CH), 137.01 (Cq), 141.30 (Cq), 142.94 (CH), 157.02 (Cq), 158.45 (Cq), 162.42 (Cq); anal. RP-HPLC: *t*_R_ 1.12 min (97.8, A), 11.44 min (98.0%, B); HRMS (ESI^+^): calcd for C_18_H_14_N_3_O_2_ [M+H]^+^ 304.1086, found 304.1095.

**10-(4-Methylphenyl)-2*H*,3*H*,4*H*,10*H*-pyrimido[4,5-*b*] quinol-ine-2,4-dione (49)**

Prepared using general method 5.7 from **3a** (R^5,6^ = H, R^7^ = Me) and **4** (R^1–4^ = H, X = F). Yellow solid (48.8 mg, 49%). Mp: 315–316 ^o^C (dec); IR (KBr): 3,437 (NH), 1,698 (C=O), 1,671 (C=O), 1,611 (C=C) cm^-1^; ^1^H-NMR: *δ* 2.46 (3H, s, Me), 6.76 (1H, d, *J* = 8.2 Hz, C9-H), 7.29 (2H, d, AA’BB’ system, *J* = 8.3 Hz, Ph 3-H), 7.46-7.53 (3H, m, C7-H & Ph 2-H), 7.73 (1H, ddd, ^4^*J* = 1.4 Hz, ^3^*J* = 8.2 Hz, C8-H), 8.22 (1H, dd, ^4^*J* = 1.4 Hz, ^3^*J* = 8.2 Hz, C6-H), 9.11 (1H, s, C5-H), 11.04 (1H, s, N3-H); ^13^C-NMR: *δ* 21.30 (CH_3_), 116.07 (Cq), 117.67 (CH), 121.41 (Cq), 124.86 (CH), 128.58 (CH), 131.16 (CH), 131.82 (CH), 135.43 (CH), 135.53 (Cq), 139.39 (Cq), 142.41 (Cq), 142.78 (CH), 156.87 (Cq), 159.19 (Cq), 162.43 (Cq); anal. RP-HPLC: *t*_R_ 1.34 min (98.6%, A), 16.20 min (100%, B); HRMS (ESI^+^): calcd for C_18_H_14_N_3_O_2_ [M+H]^+^ 304.1086, found 304.1082.

**2*H*,3*H*,4*H*,10*H*-pyrimido[4,5-*b*] quinoline-2,4-dione (50)**

Prepared using general method 5.7 from **3b** (R^9^ = H) and **4** (R^1–4^ = H, X = F). Yellow solid (51.1 mg, 26%). Mp: > 350 ^o^C; IR (KBr): 3,409 (NH), 3,177 (NH), 1,710 (C=O), 1,622 (C=O), 1,583 (C=C) cm^-1^; ^1^H-NMR: *δ* 7.51-7.57 (1H, m, C9-H), 7.82-7.89 (2H, m, C7-H & C8-H), 8.15 (1H, d, *J* = 8.2 Hz, C6-H), 9.01 (1H, s, C5-H), 11.51 (1H, s, N3-H), 11.71 (1H, s, N10-H); ^13^C-NMR: *δ* 111.52 (Cq), 125.10 (Cq), 125.65 (CH), 127.22 (CH), 130.33 (CH), 133.57 (CH), 139.50 (CH), 149.91 (Cq), 150.57 (Cq), 151.11 (Cq), 162.64 (Cq); anal. RP-HPLC: *t*_R_ 0.72 min (100%, A), 3.05 min (98.9%, B); HRMS (ESI^+^): calcd for C_11_H_8_N_3_O_2_ [M+H]^+^ 214.0616, found 214.0617.

**10-Methyl-2*H*,3*H*,4*H*,10*H*-pyrimido[4,5-*b*] quinoline-2,4-di-one (51)**

Prepared using general method 5.7 from **3b** (R^9^ = Me) and **4** (R^1–4^ = H, X = F). Yellow solid (95.0 mg, 48% yield). Mp: 360–363 ^o^C (lit.[^3^](#_ENREF_3) > 359 ^o^C); IR (KBr): 3,473 (NH), 1,714 (C=O), 1,624 (C=O), 1,607 (C=C) cm^-1^; ^1^H-NMR: *δ* 4.03 (3H, s, Me), 7.53 (1H, t, *J* = 7.5 Hz, C8-H), 7.89-7.97 (2H, m, C7-H & C9-H), 8.16 (1H, d, *J* = 7.5 Hz, C6-H), 8.98 (1H, s, C5-H), 11.07 (1H, s, N3-H); ^13^C-NMR: *δ* 32.69 (CH_3_), 115.59 (Cq), 117.18 (CH), 121.34 (Cq), 124.82 (CH), 132.05 (CH), 135.77 (CH), 141.24 (Cq), 141.94 (CH), 156.90 (Cq), 157.98Cq) 162.54 (Cq); anal. RP-HPLC: *t*_R_ 0.60 min (100%, A), 4.05 min (97.8%, B); HRMS (ESI^+^): calcd for C_12_H_10_N_3_O_2_ [M+H]^+^ 228.0773, found 228.0771.

**10-Benzyl-2*H*,3*H*,4*H*,10*H*-pyrimido[4,5-*b*] quinoline-2,4-di-one (52)**

Prepared using general method 5.7 from **3b** (R^9^ = Bn) and **4** (R^1–4^ = H, X = F). Yellow solid (71.4 mg, 71%). Mp: 346–347 ^o^C; IR (KBr): 3,447 (NH), 1,702 (C=O), 1,652 (C=O), 1,608 (C=C) cm^-1^; ^1^H-NMR: *δ* 6.00 (2H, s, CH_2_), 7.21-7.34 (5H, m, Ph-H), 7.49 (1H, t, *J* = 7.9 Hz, C7-H), 7.68 (1H, d, *J* = 7.9 Hz, C9-H), 7.82 (1H, ddd, ^4^*J* = 1.5 Hz, ^3^*J* = 7.9 Hz, C8-H), 8.20 (1H, dd, ^4^*J* = 1.5 Hz, ^3^*J* = 7.9 Hz, C6-H), 9.07 (1H, s, C5-H), 11.15 (1H, s, N3-H); ^13^C-NMR: *δ* 47.72 (CH_2_), 115.87 (Cq), 117.40 (CH), 121.74 (Cq), 124.91 (CH), 126.93 (CH), 127.74 (CH), 129.15 (CH), 132.32 (CH), 135.72 (CH), 136.02 (Cq), 140.46 (Cq), 142.51 (CH), 157.07 (Cq), 158.38 (Cq), 162.56 (Cq); anal. RP-HPLC: *t*_R_ 1.17 min (100%, A), 12.82 min (99.2%, B); HRMS (ESI^+^): calcd for C_18_H_14_N_3_O_2_ 304.1086, found 304.1065.

**General method for the alkylation of 10-aryl-2*H*,3*H*,4*H*,10*H*-pyrimido[4,5-*b*] quinoline-2,4-diones**

To the appropriate 10-aryl-2*H*,3*H*,4*H*,10*H*-pyrimido[4,5-*b*] quinoline-2,4-dione (1 eq) in MeOH was added NaOMe (2.05 eq). After stirring for 30 min, R^8^I (4 eq) was added and the solution was heated under reflux for 24 h. After cooling, the solution was evaporated and the residue was fractionated by preparative TLC (98:2 CH_2_Cl_2_–MeOH) to afford the desired 3-alkyl-10-aryl-2*H*,3*H*,4*H*,10*H*-pyrimido[4,5-*b*] quinoline-2,4-di-one.

**3-Methyl-10-phenyl-2*H*,3*H*,4*H*,10*H*-pyrimido[4,5-*b*] quinol-ine-2,4-di-one (53)**

Prepared using the above general alkylation method from **16** and MeI. Yellow solid (17.4 mg, 33%). Mp: > 350 ^o^C (lit.[^4^](#_ENREF_4) > 360 ^o^C); IR (KBr): 1,637 (C=O), 1,616 (C=O), 1,566 (C=C) cm^-1^; ^1^H-NMR: *δ* 3.23 (3H, s, Me), 6.74 (1H, d, *J* = 8.2 Hz, C9-H), 7.40-7.78 (4H, m, C7-H & Ph-H), 7.62-7.73 (2H, m, Ph 2-H), 7.75 (ddd, ^4^*J* = 1.5 Hz, ^3^*J* = 8.2 Hz, C8-H), 8.28 (1H, dd, ^4^*J* = 1.5 Hz, ^3^*J* = 8.2 Hz, C6-H), 9.19 (1H, s, C5-H); ^13^C-NMR: *δ* 28.01 (CH_3_), 115.60 (CH), 117.48 (Cq), 121.61 (Cq), 124.99 (CH), 128.81 (CH), 129.94 (CH), 130.82 (CH), 131.88 (CH), 135.58 (CH), 137.83 (Cq), 142.09 (Cq), 143.47 (CH), 156.30 (Cq), 157.62 (Cq), 161.94 (Cq); anal. RP-HPLC: *t*_R_ 1.40 min (97.9%, A), 14.05 min (100%, B); HRMS (ESI^+^): calcd for C_18_H_14_N_3_O_2_ 304.1086, found 304.1066.

**3-Ethyl-10-phenyl-2*H*,3*H*,4*H*,10*H*-pyrimido[4,5-*b*] quinoline-2,4-di-one (54)**

Prepared using the above general alkylation method from **16** and EtI. Yellow solid (10.0 mg, 18%). Mp: > 360 ^o^C; IR (KBr): 1,692 (C=O), 1,638 (C=O), 1,623 (C=C) cm^-1^; ^1^H-NMR: *δ* 1.11 (3H, t, *J* = 7.0 Hz, Me), 3.89 (2H, q, *J* = 7.0 Hz, CH_2_), 6.73 (1H, d, *J* = 8.0 Hz, C9-H), 7.43 (2H, d, *J* = 7.4 Hz, Ph 2-H), 7.51 (1H, t, *J* = 8.0 Hz, C7-H), 7.61-7.80 (4H, m, C8-H & Ph-H), 8.27 (1H, dd, ^4^*J* = 1.2 Hz, ^3^*J* = 8.0 Hz, C6-H), 9.18 (1H, s, C5-H); ^13^C-NMR: *δ* 8.40 (CH_3_), 13.48 (CH_2_), 115.83 (CH), 118.05 (Cq), 122.16 (Cq), 125.57 (CH), 127.93 (CH), 130.09 (CH), 131.13 (CH), 131.77 (CH), 135.51 (CH), 138.03 (Cq), 142.25 (Cq), 143.67 (CH), 155.20 (Cq), 158.32 (Cq), 162.93 (Cq); anal. RP-HPLC: *t*_R_ 1.99 min (100%, A), 22.15 min (100%, B); HRMS (ESI^+^): calcd for C_19_H_16_N_3_O_2_ [M+H]^+^ 318.1242, found 318.1251.

**10-Phenyl-6-(trifluoromethyl)-2*H*,3*H*,4*H*,10*H*-pyrimido[4,5-*b*]quinoline-2,4-dione (55)**

Prepared using general method 5.7 from **3a** (R^5–7^ = H) and **4** (R^1^ = CF_3_, R^2-4^ = H, X = F). Yellow solid (30.2 mg, 30%). Mp: 342–344 ^o^C (dec); IR (KBr): 3,441 (NH), 1,713 (C=O), 1,679 (C=O), 1,627 (C=C) cm^-1^; ^1^H-NMR: *δ* 7.06 (1H, d, *J* = 8.0 Hz, C9-H), 7.47 (2H, d, *J* = 7.4 Hz, Ph 2-H), 7.64-7.76 (3H, m, Ph-H), 7.88 (1H, t, *J* = 8.0 Hz, C8-H), 7.96 (1H, d, *J* = 8.0 Hz, C7-H), 8.87 (1H, s, C5-H), 11.31 (1H, s, N3-H); ^13^C-NMR: *δ* 116.91 (Cq), 118.12 (Cq), 122.86 (CH), 123.14 (CH), 123.20 (Cq),), 128.78 (CH), 130.13 (CH), 130.89 (CH), 134.57 (CH), 135.55 (CH), 137.94 (Cq), 143.48 (Cq), 156.78 (Cq), 158.78 (Cq), 162.03 (Cq); anal. RP-HPLC: *t*_R_ 2.30 min (97.4%, A), 5.45 min (100%, B); HRMS (ESI^+^): calcd for C_18_H_11_F_3_N_3_O_2_ [M+H]^+^ 358.0803, found 358.0833.

**10-Phenyl-7-(trifluoromethyl)-2*H*,3*H*,4*H*,10*H*-pyrimido[4,5-*b*]quinoline-2,4-dione (56)**

Prepared using general method 5.7 from **3a** (R^5–7^ = H) and **4** (R^1,3,4^ = H, R^2^ = CF_3_, X = F). Yellow solid (87.2 mg, 87%). Mp: 304-306 ^o^C; IR (KBr): 3,419 (NH), 1,706 (CO), 1,666 (CO), 1,619 (C=C) cm^-1^; ^1^H-NMR: *δ* 6.87 (1H, d, *J* = 9.3 Hz, C9-H), 7.46 (2H, d, *J* = 7.1 Hz, Ph 2-H), 7.63-7.74 (3H, m, Ph-H), 8.01 (1H, d, *J* = 9.3 Hz, C8-H), 8.73 (1H, s, C6-H), 9.23 (1H, s, C5-H), 11.22 (1H, s, N3-H); ^13^C-NMR: *δ* 117.57 (Cq), 118.88 (CH), 121.04 (Cq), 124.99 (Cq), 125.58 (Cq), 128.82 (CH), 129.34 (CH), 130.07 (CH), 130.84 (CH), 137.82 (CH), 142.48 (CH), 144.24 (Cq), 156.80 (Cq), 159.72 (Cq), 162.05 (Cq); anal. RP-HPLC: *t*_R_ 3.36 min (100%, A), 6.30 min (99.6%, B); HRMS (ESI^+^): calcd for C_18_H_11_F_3_N_3_O_2_ [H+H]^+^ 358.0803, found 358.0791.

**10-Phenyl-8-(trifluoromethyl)-2*H*,3*H*,4*H*,10*H*-pyrimido[4,5-*b*]quinoline-2,4-dione (57)**

Prepared using general method 5.7 from **3a** (R^5–7^ = H) and **4** (R^1,2,4^ = H, R^3^ = CF_3_, X = F). Yellow solid (66.7 mg, 67%). Mp: 347-348 ^o^C (dec); IR (KBr): 3,417 (NH), 1,706 (C=O), 1,666 (C=O), 1,616 (C=C) cm^-1^; ^1^H-NMR: *δ* 6.82 (1H, s, C9-H), 7.49 (1H, d, *J* = 8.3 Hz, C7-H), 7.65-7.76 (3H, m, Ph-H), 7.84 (2H, d, *J* = 8.6 Hz, Ph 2-H), 8.48 (1H, d, *J* = 8.3 Hz, C6-H), 9.22 (1H, s, C5-H), 11.26 (1H, s, N3-H); ^13^C-NMR: *δ* 113.91 (CH), 118.46 (Cq), 120.58 (CH), 123.81 (Cq), 128.85 (CH), 130.27 (CH), 130.95 (CH), 133.52 (CH), 133.72 (Cq), 137.54 (Cq), 141.83 (CH), 142.01 (Cq), 156.76 (Cq), 159.50 (Cq), 162.02 (Cq); anal. RP-HPLC: *t*_R_ 3.15 min (96.2%, A), 6.02 min (97.8%, B); HRMS (ESI^+^): calcd for C_18_H_11_F_3_N_3_O_2_ [H+H]^+^ 358.0803, found 358.0818.

**10-(2-Fluorophenyl)-6-(trifluoromethyl)-2*H*,3*H*,4*H*,10*H*-pyrimido[4,5-*b*]quinoline-2,4-dione (58)**

Prepared using general method 5.7 from **3a** (R^5^ = F, R^6,7^ = H) and **4** (R^1^ = CF_3_, R^2-4^ = H, X = F). Yellow solid (26.0 mg, 26%). Mp: 299–300 ^o^C; IR (KBr): 3,433 (NH), 1,720 (C=O), 1,660 (C=O), 1,625 (C=C) cm^-1^; ^1^H-NMR: *δ* 7.20 (1H, d, *J* = 8.6 Hz, C9-H), 7.52-7.69 (3H, m, Ph-H), 7.72-7.81 (1H, m, Ph-H), 7.93 (1H, t, *J* = 8.6 Hz, C8-H), 8.00 (1H, d, *J* = 8.6 Hz, C7-H), 8.87 (1H, s, C5-H), 11.37 (1H, s, N3-H); ^13^C-NMR: *δ* 116.86 (Cq), 117.82 (CH, d, *J* = 19.42 Hz), 118.02 (Cq), 122.10 (CH), 123.63 (CH), 124.59 (Cq), 124.72 (Cq), 126.82 (CH, d, *J* = 3.64 Hz), 130.88 (CH), 132.94 (CH, d, *J* = 7.93 Hz), 135.29 (CH), 136.02 (CH), 147.52 (Cq), 156.60 (Cq), 157.59 (Cq, d, *J* = 248.65 Hz), 158.43 (Cq), 161.73 (Cq); anal. RP-HPLC: *t*_R_ 2.55 min (100%, A), 5.87 min (99.5%, B); HRMS (ESI^+^): calcd for C_18_H_10_F_4_N_3_O_2_ [M+H]^+^ 376.0709, found 376.0685.

**10-(2-Fluorophenyl)-7-(trifluoromethyl)-2*H*,3*H*,4*H*,10*H*-pyrimido[4,5-*b*]quinoline-2,4-dione (59)**

Prepared using general method 5.7 from **3a** (R^5^ = F, R^6,7^ = H) and **4** (R^1,3,4^ = H, R^2^ = CF_3_, X = F). Yellow solid (63.1 mg, 63%). Mp: 308–309 ^o^C; IR (KBr): 3,415 (NH), 1,712 (C=O), 1,655 (C=O), 1,617 (C=C) cm^-1^; ^1^H-NMR: *δ* 6.99 (1H, d, *J* = 8.9 Hz, C9-H), 7.52-7.67 (3H, m, Ph-H), 7.71-7.78 (1H, m, Ph-H), 8.05 (1H, dd, ^4^*J* = 1.6 Hz, ^3^*J* = 8.9 Hz, C8-H), 8.75 (1H, d, ^4^*J* = 1.6 Hz, C6-H), 9.24 (1H, s, C5-H), 11.32 (1H, s, N3-H); ^13^C-NMR: *δ* 117.39 (Cq), 117.78 (CH, d, *J* = 18.91 Hz), 118.11 (CH), 121.11 (CH), 124.36 (Cq, d, *J* = 13.59 Hz), 125.45 (Cq), 126.75 (CH, d, *J*= 3.14 Hz), 129.71 (CH), 130.91 (CH), 131.46 (Cq), 132.87 (CH, d, *J* = 7.74 Hz), 142.98 (CH), 143.37 (Cq), 156.73 (Cq), 157.48 (Cq, d, *J* = 250.50 Hz), 159.38 (Cq), 161.82 (Cq); anal. RP-HPLC: *t*_R_ 3.47 min (98.2%, A), 6.74 min (96.8%, B); HRMS (ESI^+^): calcd for C_18_H_10_F_4_N_3_O_2_ [M+H]^+^ 376.0709, found 376.0727.

**10-(2-Chlorophenyl)-9-(trifluoromethyl)-2*H*,3*H*,4*H*,10*H*-pyrimido[4,5-*b*]quinoline-2,4-dione (60)**

Prepared using general method 5.7 from **3a** (R^5^ = Cl, R^6,7^ = H) and **4** (R^1–3^ = H, R^4^ = CF_3_, X = F). Yellow solid (10.7 mg, 11%). Mp: 324–325 ^o^C (dec); IR (KBr): 3,421 (NH), 1,703 (C=O), 1,679 (C=O), 1,623 (C=C) cm^-1^; ^1^H-NMR: *δ* 7.50-7.64 (3H, m), 7.66-7.73 (2H, m), 8.26 (1H, dd, ^4^*J* = 1.5 Hz, ^3^*J* = 7.8 Hz, C8-H), 8.56 (1H, dd, ^4^*J* = 1.5 Hz, ^3^*J* = 7.8 Hz, C6-H), 9.16 (1H, s, C5-H), 11.32 (1H, s, N3-H); ^13^C-NMR: *δ* 116.71 (Cq), 124.08 (Cq), 125.05 (CH), 127.36 (CH), 130.31 (CH), 131.86 9 (CH), 132.52 (Cq), 134.75 (CH), 135.94 (CH), 136.01 (Cq), 136.88 (Cq), 137.86 (CH), 139.92 (Cq), 143.80 (CH), 156.52 (Cq), 160.74 (Cq), 161.78 (Cq); anal. RP-HPLC: *t*_R_ 2.88 min (97.8%, A), 34.63 min (98.0%, B); HRMS (ESI^+^): calcd for C_18_H_10_ClF_3_N_3_O_2_ [M+H]^+^ 392.0414, found 392.0449.

**10-(2-Methylphenyl)-9-(trifluoromethyl)-2*H*,3*H*,4*H*,10*H*-pyrimido[4,5-*b*]quinoline-2,4-dione (61)**

Prepared using general method 5.7 from **3a** (R^5^ = Me, R^6,7^ = H) and **4** (R^1–3^ = H, R^4^ = CF_3_, X = F). Yellow solid (24.0 mg, 24%). Mp: 347–348 ^o^C (dec); IR (KBr): 3,421 (NH), 1,703 (C=O), 1,677 (C=O), 1,623 (C=C) cm^-1^; ^1^H-NMR: *δ* 1.89 (3H, s, Me), 7.18 (1H, d, *J* = 7.6 Hz, Ph 3-H), 7.28 (1H, ddd, ^4^*J* = 1.5 Hz, ^3^*J* = 7.6 Hz, Ph 4-H), 7.33-7.43 (2H, m, Ph-H), 7.67 (1H, t, *J* = 7.7 Hz, C7-H), 8.22 (1H, dd, ^4^*J* = 1.3 Hz, ^3^*J* = 7.7 Hz, C8-H), 8.51 (1H, dd, ^4^*J* = 1.3 Hz, ^3^*J* = 7.7 Hz, C6-H), 9.12 (1H, s, C5-H), 11.23 (1H, s, N3-H); ^13^C-NMR: *δ* 18.32 (CH_3_), 116.89 (Cq), 117.99 (Cq), 118.62 (Cq), 124.19 (CH), 124.73 (Cq), 126.33 (CH), 129.92 (CH), 130.82 (CH), 131.19 (CH), 137.45 (CH), 137.78 (CH), 139.51 (Cq), 140.36 (Cq), 143.20 (CH), 156.86 (Cq), 160.61 (Cq), 162.10 (Cq); anal. RP-HPLC: *t*_R_ 2.68 min (98.5%, A), 6.22 min (97.5%, B); HRMS (ESI^+^): calcd for C_19_H_13_F_3_N_3_O_2_ [M+H]^+^ 372.0960, found 372.0938.

**10-(4-Chlorophenyl)-3-methyl-9-(trifluoromethyl)-2*H*,3*H*, 4*H*,10*H*-pyrimido[4,5-*b*]quinoline-2,4-dione (62)**

Prepared using the above general alkylation method from **43** and MeI. Yellow solid (6.8 mg, 22%). Mp: > 360 ^o^C; IR (KBr): 1,700 (C=O), 1,681 (C=O), 1,620 (C=C) cm^-1^; ^1^H-NMR: *δ* 5.75 (3H, s, Me), 7.39 (2H, d, AA’BB’ system, *J* = 8.7 Hz, Ph 2-H), 7.58 (2H, d, AA’BB’ system, *J* = 8.7 Hz, Ph 3-H), 7.70 (1H, t, *J* = 7.8 Hz, C7-H), 8.23 (1H, dd, ^4^*J* = 1.4 Hz, ^3^*J* = 7.8 Hz, C8-H), 8.54 (1H, dd, ^4^*J* = 1.4 Hz, ^3^*J* = 7.8 Hz, C6-H), 9.18 (1H, s, C5-H); ^13^C-NMR: *δ* 21.08 (CH_3_), 115.84 (Cq), 118.34 (Cq), 123.98 (Cq), 125.12 (CH), 128.68 (CH), 133.86 (CH), 134.57 (Cq), 135.21 (CH), 137.77 (CH), 139.85 (Cq), 140.45 (Cq), 144.17 (CH), 157.04 (Cq), 161.84 (Cq), 162.27 (Cq); anal. RP-HPLC: *t*_R_ 6.43 min (99.1%, A), 5.12 min (96.8%, B); HRMS (ESI^+^): calcd for C_19_H_12_ClF_3_N_3_O_2_ [M+H]^+^ 406.0570, found 406.0571.

**10-(4-Chlorophenyl)-3-ethyl-9-(trifluoromethyl)-2*H*,3*H*,4*H*, 10*H*-pyrimido[4,5-*b*]quinoline-2,4-dione (63)**

Prepared using the above general alkylation method from **43** and EtI. Yellow solid (9.3 mg, 43%). Mp: 278–280 ^o^C (dec); IR (KBr): 1,646 (C=O), 1,611 (C=O), 1,538 (C=C) cm^-1^; ^1^H-NMR: *δ* 1.10 (3H, t, *J* = 7.9 Hz, Me), 3.87 (2H, q, *J* = 7.9 Hz, CH_2_), 7.39 (2H, d, AA’BB’ system, *J* = 8.6 Hz, Ph 2-H), 7.57 (2H, d, AA’BB’ system, *J* = 8.6 Hz, Ph 3-H), 7.69 (1H, t, *J* = 7.8 Hz, C7-H), 8.22 (1H, dd, ^4^*J* = 1.4 Hz, ^3^*J* = 7.8 Hz, C8-H), 8.53 (1H, dd, ^4^*J* = 1.4 Hz, ^3^*J* = 7.8 Hz, C6-H), 9.17 (1H, s, C5-H); ^13^C-NMR: *δ* 9.31 (CH_3_), 14.88 (CH_2_), 115.88 (Cq), 117.50 (Cq), 124.28 (Cq), 125.11 (CH), 128.52 (CH), 133.32 (CH), 134.67 (Cq), 136.16 (CH), 137.61 (CH), 139.47 (Cq), 140.94 (Cq), 144.28 (CH), 157.10 (Cq), 161.74 (Cq), 162.16 (Cq); anal. RP-HPLC: *t*_R_ 0.75 min (98.3%, A), 19.62 min (99.4%, B); HRMS (ESI^+^): calcd for C_20_H_14_ClF_3_N_3_O_2_ [H+H]^+^ 420.0727, found 420.0692

**9-(Trifluoromethyl)-2*H*,3*H*,4*H*,10*H*-pyrimido[4,5-*b*] quinol-ine-2,4-dione (64)**

Prepared using general method 5.7 from **3b** (R^9^ = H) and **4** (R^1–3^ = H, R^4^ = CF_3_, X = F). Yellow solid (73.8 mg, 74%). Mp: > 350 ^o^C; IR (KBr): 3,429 (NH), 3,071 (NH), 1,711 (C=O), 1,654 (C=O), 1,610 (C=C) cm^-1^; ^1^H-NMR: *δ* 7.69 (1H, t, *J* = 7.6 Hz, C7-H), 8.25 (1H, d, *J* = 7.6 Hz, C8-H), 8.45 (1H, d, *J* = 7.6 Hz, C6-H), 9.14 (1H, s, C5-H), 11.64 (1H, s, N3-H), 11.80 (1H, s, N10-H); ^13^C-NMR: *δ* 112.57 (Cq), 124.32 (CH), 125.65 (Cq), 131.76 (CH), 131.86 (Cq), 135.30 (CH), 139.94 (CH), 146.13 (Cq), 151.02 (Cq), 151.11 (Cq), 162.36 (Cq); anal. RP-HPLC: *t*_R_ 1.59 min (99.2%, A), 6.74 min (100%, B); HRMS (ESI^+^): calcd for C_12_H_7_F_3_N_3_O_2_ [M+H]^+^ 282.0490, found 282.0505.

**10-Methyl-9-(trifluoromethyl)-2*H*,3*H*,4*H*,10*H*-pyrimido[4,5-*b*]quinol-ine-2,4-dione (65)**

Prepared using general method 5.7 from **3b** (R^9^ = Me) and **4** (R^1–3^ = H, R^4^ = CF_3_, X = F). Purified by prep. RP-HPLC (Phenomenex Kromasil C18 column, 250 × 21.2 mm; flow rate 14.10 mL/min, linear gradient of from 0 to 30% MeCN in H_2_O over 10 min. Yellow solid (3.39 mg, 3%). Mp: 318–320 ^o^C; IR (KBr): 3,438 (NH), 1,713 (C=O), 1,664 (C=O), 1,618 (C=C) cm^-1^; ^1^H-NMR: *δ* 3.85 (3H, s, Me), 7.67 (1H, t, *J* = 7.8 Hz, C7-H), 8.35 (1H, d, *J* = 7.8 Hz, C8-H), 8.44 (1H, d, *J* = 7.8 Hz, C6-H), 9.00 (1H, s, C5-H), 11.59 (1H, s, N3-H); ^13^C-NMR: *δ* 45.11 (CH_3_), 116.56 (Cq), 124.10 (Cq), 124.45 (CH), 132.47 (Cq), 135.92 (CH), 137.03 (CH), 140.62 (Cq), 142.01 (CH), 157.01 (Cq), 160.74 (Cq), 162.19 (Cq); anal. RP-HPLC: *t*_R_ 0.79 min (99.4%, A), 7.95 min (99.6%, B); HRMS (ESI^+^): calcd for C_13_H_9_F_3_N_3_O_2_ [M+H]^+^ 296.0647, found 296.0643.

**6-Chloro-10-phenyl-2*H*,3*H*,4*H*,10*H*-pyrimido[4,5-*b*]quinol-ine-2,4-dione (66)**

Prepared using general method 5.7 from **3a** (R^5–7^ = H) and **4** (R^1^ = Cl, R^2–4^ = H, X = F). Yellow solid (19.7 mg, 20%). Mp 339–341 ^o^C (lit.[^1^](#_ENREF_1) 338–340 ^o^C, dec); IR (KBr): 3,419 (NH), 1,707 (C=O), 1,674 (C=O), 1,613 (C=C) cm^-1^; ^1^H-NMR: *δ* 6.69 (1H, dd, ^4^*J* = 1.2 Hz, ^3^*J* = 7.8 Hz, C9-H), 7.43 (2H, d, *J* = 7.3 Hz, Ph 2-H), 7.62-7.74 (5H, m, C8-H & C7-H & Ph-H), 9.03 (1H, s, C5-H), 11.25 (1H, s, N3-H); ^13^C-NMR: *δ* 117.23 (Cq), 117.27 (CH), 118.98 (Cq), 125.35 (CH), 128.82 (CH), 130.03 (CH), 130.79 (CH), 134.24 (Cq), 135.64 (CH), 137.02 (CH), 138.05 (Cq), 143.66 (Cq), 156.77 (Cq), 159.03 (Cq), 162.07 (Cq); anal. RP-HPLC: *t*_R_ 1.49 min (98.4%, A), 4.52 min (96.3%, B); HRMS (ESI^+^): calcd for C_17_H_11_ClN_3_O_2_ [M+H]^+^ 324.0540, found 324.0518.

**7-Chloro-10-phenyl-2*H*,3*H*,4*H*,10*H*-pyrimido[4,5-*b*]quinol-ine-2,4-dione (67)**

Prepared using general method 5.7 from **3a** (R^5–7^ = H) and **4** (R^1,3,4^ = H, R^2^ = Cl, X = F). Yellow solid (65 mg, 65%). Mp: 295–296 ^o^C (dec); IR (KBr): 3,420 (NH), 1,702 (C=O), 1,663 (C=O), 1,614 (C=C) cm^-1^; ^1^H-NMR: *δ* 6.70 (1H, d, *J* = 9.1 Hz, C9-H), 7.43 (2H, d, *J* = 7.3 Hz, Ph 2-H), 7.61-7.72 (3H, m, Ph-H), 7.75 (1H, dd, ^4^*J* = 2.5 Hz, ^3^*J* = 9.1 Hz, C8-H), 8.37 (1H, d, ^4^*J* = 2.5 Hz, C6-H), 9.09 (1H, s, C5-H), 11.16 (1H, s, N3-H); ^13^C-NMR: *δ* 117.24 (Cq), 119.64 (CH), 122.47 (Cq), 128.80 (CH), 128.87 (Cq), 129.97 (CH), 130.23 (CH), 130.79 (CH), 135.03 (CH), 137.92 (Cq), 141.05 (Cq), 141.72 (CH), 156.82 (Cq), 159.08 (Cq), 162.17 (Cq); anal. HPLC: *t*_R_ 1.85 min (97.5%, A), 4.97 min (100%, B); HRMS (ESI^+^): calcd for C_17_H_11_ClN_3_O_2_ [M+H]^+^ 324.0540, found 324.0531.

**8-Chloro-10-phenyl-2*H*,3*H*,4*H*,10*H*-pyrimido[4,5-*b*]quinol-ine-2,4-dione (68)**

Prepared using general method 5.7 from **3a** (R^5–7^ = H) and **4** (R^1,2,4^ = H, R^3^ = Cl, X = F). Yellow solid (35.5 mg, 36%). Mp: > 350 ^o^C (lit.[^1^](#_ENREF_1) > 360 ^o^C); IR (KBr): 3,429 (NH), 1,702 (C=O), 1,663 (C=O), 1,608 (C=C) cm^-1^; ^1^H-NMR: *δ* 6.57 (1H, s, C9-H), 7.45 (2H, d, *J* = 7.1 Hz, Ph 2-H), 7.58 (1H, dd, ^4^*J* = 1.5 Hz, ^3^*J* = 8.6 Hz, C7-H), 7.63-7.74 (3H, m, Ph-H), 8.27 (1H, d, *J* = 8.6 Hz, C6-H), 9.13 (1H, s, C5-H), 11.16 (1H, s, N3-H); ^13^C-NMR: *δ* 116.43 (Cq), 116.68 (CH), 120.30 (Cq), 125.21 (CH), 128.81 (CH), 130.13 (CH), 130.89 (Cq), 133.71 (CH), 137.67 (Cq), 139.84 (Cq), 142.20 (CH), 142.98 (Cq), 156.82 (Cq), 159.33 (Cq), 162.20 (Cq); anal. RP-HPLC: *t*_R_ 1.68 min (96.2%, A), 4.82 min (97.5%, B); HRMS (ESI^+^): calcd for C_17_H_11_ClN_3_O_2_ [M+H]^+^ 324.0540, found 324.0565.

**6-Chloro-10-(2-fluorophenyl)-2H,3H,4H,10H-pyrimido- [4,5-b]quinoline-2,4-dione (69)**

Prepared using general method 5.7 from **3a** (R^5,6^ = H, R^7^ = Cl; 65 mg, 0.29 mmol)[^2^](#_ENREF_2) and **4** (R^1^ = F, R^2–4^ = H, X = F; 55 mg, 0.35 mmol). Yellow solid (28.0 mg, 28%). Mp: 322–323 ^o^C (lit.[^1^](#_ENREF_1) 323–324 ^o^C); IR (KBr): 3,464 (NH), 1,710 (C=O), 1,678 (C=O), 1,620 (C=C) cm^-1^; ^1^H-NMR: *δ* 6.82 (1H, d, *J* = 8.4 Hz, C9-H), 7.51-7.66 (3H, m), 7.70-7.79 (3H, m), 9.04 (1H, s, C5-H),11.34 (1H, s, N3-H); ^13^C-NMR: *δ* 116.44 (CH), 117.05 (Cq), 117.75 (CH, d, *J* = 18.7 Hz), 118.98 (Cq), 124.84 (Cq, d, *J* =12.9 Hz), 125.85 (CH), 126.71 (CH, d, *J* = 3.6 Hz), 130.90 (CH), 132.81(CH, d, *J* = 7.5 Hz), 134.61 (Cq), 136.31 (CH), 137.61 (CH), 142.77 (Cq), 156.65 (Cq), 157.44 (Cq, d, *J* = 249.9 Hz), 158.74 (Cq), 161.81 (Cq); anal. RP-HPLC: *t*_R_ 1.70 min (100%, A), 4.89 min (96.8%, B); HRMS (ESI^+^): calcd for C_17_H_10_ClFN_3_O_2_ [M+H]^+^ 342.0446, found 342.0414.

**8-Chloro-10-(2-fluorophenyl)-2*H*,3*H*,4*H*,10*H*-pyrimido[4,5-*b*]quinoline-2,4-dione (70)**

Prepared using general method 5.7 from **3a** (R^5^ = F, R^6,7^ = H) and **4** (R^1,2,4^ = H, R^3^ = Cl, X = F). Yellow solid (48.3 mg, 48%). Mp: > 350 ^o^C; IR (KBr): 3,432 (NH), 1,700 (C=O), 1,668 (C=O), 1,608 (C=C) cm^-1^; ^1^H-NMR: *δ* 6.73 (1H, s, C9-H), 7.55 (1H, t, *J* = 7.4 Hz, Ph 5-H), 7.61-7.67 (3H, m, C7-H & Ph-H), 7.72-7.79 (1H, m, Ph-H), 8.31 (1H, d, *J* = 8.5 Hz, C6-H), 9.17 (1H, s, C5-H), 11.25 (1H, s, N3-H); ^13^C-NMR: *δ* 115.90 (CH), 116.21 (Cq), 117.86 (CH, d, *J* = 19.01 Hz), 120.29 (Cq), 124.43 (Cq, d, *J* = 13.26 Hz), 125.74 (CH), 126.75 (CH), 130.88 (CH), 132.90 (CH, d, *J* = 8.01 Hz), 134.07 (CH), 140.49 (Cq), 142.11 (Cq), 142.78 (CH), 156.75 (Cq), 157.32 (Cq, d, *J* = 250.76 Hz), 159.04 (Cq), 161.96 (Cq); anal. RP-HPLC: *t*_R_ 1.93 min (98.4%, A), 5.10 min (97.3%, B); HRMS (ESI^+^): calcd for C_17_H_10_ClFN_3_O_2_ [M + H]^+^ 342.0445, found 342.0467.

**9-Chloro-10-(2-fluorophenyl)-2*H*,3*H*,4*H*,10*H*-pyrimido[4,5-*b*]quinoline-2,4-dione (71)**

Prepared using general method 5.7 from **3a** (R^5^ = F, R^6,7^ = H) and **4** (R^1–3^ = H, R^4^ = Cl, X = F). Yellow solid (12.4 mg, 12%). Mp: 298–300 ^o^C; IR (KBr): 3,423 (NH), 1,709 (C=O), 1,678 (C=O), 1,619 (C=C) cm^-1^; ^1^H-NMR: *δ* 7.34 (1H, t, *J* = 7.55 Hz, Ph 5-H), 7.41-7.48 (2H, m, Ph-H), 7.52 (1H, t, *J* = 8.0 Hz, C7-H), 7.58-7.65 (1H, m, Ph-H), 7.88 (1H, dd, ^4^*J* = 1.2 Hz, ^3^*J* = 8.0 Hz, C8-H), 8.26 (1H, dd, ^4^*J* = 1.2 Hz, ^3^*J* = 8.0 Hz, C6-H), 9.12 (1H, s, C5-H), 11.27 (1H, s, N3-H); ^13^C-NMR: *δ* 116.27 (CH, d, *J* = 19.36 Hz), 116.31 (Cq), 120.66 (Cq), 124.56 (Cq), 125.32 (CH, d, *J* = 2.72 Hz), 126.07 (CH), 127.30 (Cq, d, *J* = 13.69 Hz), 131.24 (CH), 132.23 (CH, d, *J* = 7.90 Hz), 132.61 (CH), 137.19 (Cq), 139.20 (CH), 143.65 (CH), 156.65 (Cq), 158.98 (Cq, d, *J* = 248.75 Hz), 160.30 (Cq), 161.86 (Cq); anal. RP-HPLC: *t*_R_ 1.80 min (96.8%, A), 4.95 min (99.1%, B); HRMS (ESI^+^): calcd for C_17_H_10_ClFN_3_O_2_ [M+H]^+^ 342.0445, found 342.0446.

**7-Chloro-10-(4-chlorophenyl)-2*H*,3*H*,4*H*,10*H*-pyrimido[4,5-*b*]quinoline-2,4-dione (72)**

Prepared using general method 5.7 from **3a** (R^5,6^ = H, R^7^ = Cl) and **4** (R^1,3,4^ = H, R^2^ = Cl, X = F). Yellow solid (47.3 mg, 47%). Mp: > 350 ^o^C; IR (KBr): 3,406 (NH), 1,704 (C=O), 1,665 (C=O), 1,615 (C=C) cm^-1^; ^1^H-NMR: *δ* 6.80 (1H, d, *J* = 9.0 Hz, C9-H), 7.47-7.53 (2H, d, AA’BB’ system, *J* = 8.4 Hz, Ph 2-H), 7.72-7.79 (3H, m, C8-H & Ph 3-H), 8.38 (1H, d, ^4^*J* = 2.1 Hz, C6-H), 9.09 (1H, s, C5-H), 11.19 (1H, s, N3-H); ^13^C-NMR: *δ* 117.21 (Cq), 119.64 (CH), 122.48 (Cq), 128.97 (Cq), 130.28 (CH), 130.86 (CH), 130.90 (CH), 134.67 (Cq), 135.04 (CH), 136.72 (Cq), 140.80 (Cq), 141.79 (CH), 156.67 (Cq), 159.11 (Cq), 162.01 (Cq); anal. RP-HPLC: *t*_R_ 3.42 min (100%, A), 6.90 min (98.6%, B); HRMS (ESI^+^): calcd for C_17_H_10_Cl_2_N_3_O_2_ [M+H]^+^ 358.0150, found 358.0152.

**8-Chloro-10-(4-chlorophenyl)-2*H*,3*H*,4*H*,10*H*-pyrimido[4,5-*b*]quinoline-2,4-dione (73)**

Prepared using general method 5.7 from **3a** (R^5,6^ = H, R^7^ = Cl) and **4** (R^1,2,4^ = H, R^3^ = Cl, X = F). Yellow solid (22.7 mg, 23%). Mp: > 350 ^o^C (lit.[^2^](#_ENREF_2) > 300 ^o^C); IR (KBr): 3,432 (NH), 1,701 (C=O), 1,664 (C=O), 1,609 (C=C) cm^-1^; ^1^H-NMR: *δ* 6.70 (1H, d, ^4^*J* = 1.7 Hz, C9-H), 7.51 (2H, d, AA’BB’ system, *J* = 8.6 Hz, Ph 2-H), 7.60 (1H, dd, ^4^*J* = 1.7 Hz, ^3^*J* = 8.6 Hz, C7-H), 7.78 (2H, d, AA’BB’ system, *J* = 8.6 Hz, Ph 3-H), 8.28 (1H, d, *J* = 8.6 Hz, C6-H), 9.13 (1H, s, C5-H), 11.61 (1H, s, N3-H); ^13^C-NMR: *δ* 116.39 (Cq), 116.65 (CH), 120.32 (Cq), 125.37 (CH), 130.90 (CH), 131.02 (CH), 133.74 (CH), 134.78 (Cq), 136.44 (Cq), 140.05 (Cq), 142.29 (CH), 142.81 (Cq), 156.74 (Cq), 159.45 (Cq), 162.16 (Cq); anal. RP-HPLC: *t*_R_ 3.54 min (100%, A), 6.82 min (100%, B); HRMS (ESI^+^): calcd for C_17_H_10_Cl_2_N_3_O_2_ [M+H]^+^ 358.0150, found 358.0146.

**9-Chloro-10-(2-chlorophenyl)-2*H*,3*H*,4*H*,10*H*-pyrimido[4,5-*b*]quinoline-2,4-dione (74)**

Prepared using general method 5.7 from **3a** (R^5^ = Cl, R^6,7^ = H) and **4** (R^1–3^ = H, R^4^ = Cl, X = F). Yellow solid (16.1 mg, 16%). Mp: 312–313 ^o^C (dec); IR (KBr): 3,434 (NH), 1,702 (C=O), 1,675 (C=O), 1,618 (C=C) cm^-1^; ^1^H-NMR: *δ* 7.49-7.54 (2H, m), 7.55-7.62 (2H, m), 7.65-7.69 (1H, m), 7.89 (1H, dd, ^4^*J* = 1.3 Hz, ^3^*J* = 7.9 Hz, C8-H), 8.27 (1H, dd, ^4^*J* = 1.3 Hz, ^3^*J* = 7.9 Hz, C6-H), 9.15 (1H, s, C5-H), 11.28 (1H, s, N3-H); ^13^C-NMR: *δ* 116.24 (Cq), 120.37 (Cq), 124.35 (Cq), 126.01 (CH), 128.37 (CH), 129.89 (CH), 131.66 (CH), 132.06 (CH), 132.83 (CH), 133.50 (Cq), 136.71 (Cq), 137.01 (Cq), 139.44 (CH), 143.83 (CH), 156.60 (Cq), 160.02 (Cq), 161.82 (Cq); anal. RP-HPLC: *t*_R_ 2.13 min (100%, A), 26.75 min (99.2%, B); HRMS (ESI^+^): calcd for C_17_H_10_Cl_2_N_3_O_2_ [M+H]^+^ 358.0150, found 358.0148.

**9-Chloro-10-(4-fluorophenyl)-2*H*,3*H*,4*H*,10*H*-pyrimido[4,5-*b*]quinoline-2,4-dione (75)**

Prepared using general method 5.7 from **3a** (R^5,6^ = H, R^7^ = F) and **4** (R^1–3^ = H, R^4^ = Cl, X = F). Yellow solid (37.1 mg, 37%). Mp: > 360 ^o^C; IR (KBr): 3,416 (NH), 1,716 (C=O), 1,657 (C=O), 1,614 (C=C) cm^-1^; ^1^H-NMR: *δ* 7.33-7.40 (2H, m, Ph 2-H), 7.43-7.52 (3H, m, C7-H & Ph 3-H), 7.85 (1H, dd, ^4^*J* = 1.4 Hz, ^3^*J* = 7.9 Hz, C8-H), 8.24 (1H, dd, ^4^*J* = 1.40 Hz, ^3^*J* = 7.9 Hz, C6-H), 9.09 (1H, s, C5-H), 11.18 (1H, s, N3-H); ^13^C-NMR: *δ* 115.98 (CH, d, *J* = 22.72 Hz), 116.42 (Cq), 121.10 (Cq), 124.58 (Cq), 125.74 (CH), 132.37 (CH, d, *J* = 4.06 Hz), 132.43 (CH), 135.80 (Cq, d, *J* = 3.27 Hz), 137.68 (Cq), 139.28 (CH), 143.32 (CH), 156.70 (Cq), 160.94 (Cq), 161.70 (Cq, d, *J* = 244.07 Hz), 162.05 (Cq); anal. RP-HPLC: *t*_R_ 1.63 min (97.4%, A), 20.08 min (98.7%, B); HRMS (ESI^+^): calcd for C_17_H_10_ClFN_3_O_2_ [M+H]^+^ 342.0445, found 342.0465.

**9-Chloro-10-(2-methylphenyl)-2*H*,3*H*,4*H*,10*H*-pyrimido[4,5-*b*]quinoline-2,4-dione (77)**

Prepared using general method 5.7 from **3a** (R^5^ = Me, R^6,7^ = H) and **4** (R^1–3^ = H, R^4^ = Cl, X = F). Yellow solid (56.5 mg, 57%). Mp: 292–293 ^o^C (dec); IR (KBr): 3,441 (NH), 1,715 (C=O), 1,656 (C=O), 1,613 (C=C) cm^-1^; ^1^H-NMR: *δ* 2.36 (3H, s, Me), 7.18-7.24 (2H, m, Ph-H), 7.31-7.36 (1H, m, Ph-H), 7.38-7.43 (1H, m, Ph-H), 7.47 (1H, t, *J* = 7.7 Hz, C7-H), 7.83 (1H, dd, ^4^*J* = 1.4 Hz, ^3^*J* = 7.7 Hz, C8-H), 8.23 (1H, dd, ^4^*J* = 1.4 Hz, ^3^*J* = 7.7 Hz, C6-H), 9.08 (1H, s, C5-H), 11.16 (1H, s, N3-H); ^13^C-NMR: *δ* 21.30 (CH_3_), 116.41 (Cq), 121.29 (Cq), 124.56 (Cq), 125.69 (CH), 127.65 (CH), 128.86 (CH), 130.08 (CH), 130.45 (CH), 132.32 (CH), 137.66 (Cq), 138.52 (Cq), 139.26 (CH), 139.48 (Cq), 143.21 (CH), 156.74 (Cq), 160.73 (Cq), 162.04 (Cq); anal. RP-HPLC: *t*_R_ 2.20 min (97.6%, A), 32.08 min (96.5%, B); HRMS (ESI^+^): calcd for C_18_H_13_ClN_3_O_2_ [M+H]^+^ 338.0696, found 338.0718.

**9-Chloro-10-(3-methylphenyl)-2*H*,3*H*,4*H*,10*H*-pyrimido[4,5-*b*]quinoline-2,4-dione (78)**

Prepared using general method 5.7 from **3a** (R^5,7^ = H, R^6^ = Me) and **4** (R^1–3^ = H, R^4^ = Cl, X = F). Yellow solid (16.9 mg, 17%). Mp: 342–343 ^o^C; IR (KBr): 3,417 (NH), 1,712 (C=O), 1,671 (C=O), 1,619 (C=C) cm^-1^; ^1^H-NMR: *δ* 2.04 (3H, s, Me), 7.19 (1H, dd, *J* = 1.0 Hz, *J* = 7.6 Hz, Ph 4-H), 7.31 (1H, t, *J* = 7.6 Hz, Ph 5-H), 7.36-7.45 (2H, m, Ph-H), 7.49 (1H, t, *J* = 7.8 Hz, C7-H), 7.84 (1H, dd, ^4^*J* = 1.4 Hz, ^3^*J* = 7.8 Hz, C8-H), 8.25 (1H, dd, ^4^*J* = 1.4 Hz, ^3^*J* = 7.8 Hz, C6-H), 9.11 (1H, s, C5-H), 11.18 (1H, s, N3-H); ^13^C-NMR: *δ* 18.10 (CH_3_), 116.53 (Cq), 120.90 (Cq), 124.58 (Cq), 125.74 (CH), 127.07 (CH), 129.38 (CH), 129.83 (CH), 130.65 (CH), 132.53 (CH), 137.26 (Cq), 137.61 (Cq), 139.02 (Cq), 139.23 (CH), 143.23 (CH), 156.87 (Cq), 159.97 (Cq), 162.10 (Cq); anal. RP-HPLC: *t*_R_ 1.97 min (96.8%, A), 24.47 min (97.2%, B); HRMS (ESI^+^): calcd for C_18_H_13_ClN_3_O_2_ [M+H]^+^ 338.0696, found 338.0653.

**9-Chloro-10-(4-methylphenyl)-2*H*,3*H*,4*H*,10*H*-pyrimido[4,5-*b*]quinoline-2,4-dione (79)**

Prepared using general method 5.7 from **3a** (R^5,6^ = H, R^7^ = Me) and **4** (R^1–3^ = H, R^4^ = Cl, X = F). Yellow solid (19.0 mg, 19%). Mp: > 350 ^o^C; IR (KBr): 3,418 (NH), 1,717 (C=O), 1,660 (C=O), 1,615 (C=C) cm^-1^; ^1^H-NMR: *δ* 2.42 (3H, s, Me), 7.25 (2H, d, AA’BB’ system, *J* = 8.2 Hz, Ph 3-H), 7.31 (2H, d, AA’BB’ system, *J* = 8.2 Hz, Ph 2-H), 7.47 (1H, t, *J* = 7.7 Hz, C7-H), 7.82 (1H, dd, ^4^*J* = 1.4 Hz, ^3^*J* = 7.7 Hz, C8-H), 8.22 (1H, dd, ^4^*J* = 1.4 Hz, ^3^*J* = 7.7 Hz, C6-H), 9.07 (1H, s, C5-H), 11.15 (1H, s, N3-H); ^13^C-NMR: *δ* 21.38 (CH_3_), 116.39 (Cq), 121.40 (Cq), 124.57 (Cq), 125.63 (CH), 129.58 (CH), 130.09 (CH), 132.31 (CH), 137.05 (Cq), 137.83 (Cq), 138.88 (Cq), 139.22 (CH), 143.17 (CH), 156.76 (Cq), 160.88 (Cq), 162.16 (Cq); anal. RP-HPLC: *t*_R_ 2.38 min (97.6%, A), 5.90 min (100%, B); HRMS (ESI^+^): calcd for C_18_H_13_ClN_3_O_2_ [M+H]^+^ 338.0696, found 338.0701.

**6-Methyl-10-phenyl-2*H*,3*H*,4*H*,10*H*-pyrimido[4,5-*b*]quinol-ine-2,4-dione (80)**

Prepared using general method 5.7 from **3a** (R^5–7^ = H) and **4** (R^1^ = Me, R^2–4^ = H, X = F). Yellow solid (382.9 mg, 48%). Mp: 342–344 ^o^C (dec); IR (KBr): 3,371 (NH), 1,700 (C=O), 1,651 (C=O), 1,598 (C=C) cm^-1^; ^1^H-NMR: *δ* 2.75 (3H, s, Me), 6.55 (1H, d, *J* = 8.8 Hz, C9-H), 7.33-7.43 (3H, m, C7-H & Ph 2-H), 7.57-7.72 (4H, m, C8-H & Ph-H), 9.02 (1H, s, C5-H), 11.09 (1H, s, N3-H); ^13^C-NMR: *δ* 18.15 (CH_3_), 114.85 (Cq), 115.51 (CH), 119.88 (Cq), 125.61 (CH), 128.41 (CH), 129.34 (CH), 130.24 (CH), 134.87 (CH), 137.96 (Cq), 138.45 (CH), 139.15 (Cq), 142.42 (Cq), 156.42 (Cq), 158.30 (Cq), 162.06 (Cq); anal. RP-HPLC: *t*_R_ 1.18 min (98.8%, A), 4.03 min (97.7%, B); HRMS (ESI^+^): calcd for C_18_H_14_N_3_O_2_ [M+H]^+^ 304.1086, found 304.1085.

**7-Methyl-10-phenyl-2*H*,3*H*,4*H*,10*H*-pyrimido[4,5-*b*]quinol-ine-2,4-dione (81)**

Prepared using general method 5.7 from **3a** (R^5–7^ = H) and **4** (R^1,3,4^ = H, R^2^ = Me, X = F). Yellow solid (54.8 mg, 55%). Mp: 342–344 ^o^C (dec); IR (KBr): 3,437 (NH), 1,703 (C=O), 1,649 (C=O), 1,609 (C=C) cm^-1^; ^1^H-NMR: *δ* 2.40 (3H, s, Me), 6.62 (1H, d, *J* = 8.8 Hz, C9-H), 7.41 (2H, d, *J* = 7.7 Hz, Ph 2-H), 7.57 (1H, dd, ^4^*J* = 1.74 Hz, ^3^*J* = 8.8 Hz, C8-H), 7.60-7.72 (3H, m, Ph-H), 8.00 (1H, s, C6-H), 9.02 (1H, s, C5-H), 11.04 (1H, s, N3-H); ^13^C-NMR: *δ* 20.64 (CH_3_), 115.99 (Cq), 117.50 (CH), 121.41 (Cq), 128.84 (CH), 129.77 (CH), 130.66 (CH), 130.84 (CH), 134.41 (Cq), 136.95 (CH), 138.18 (Cq), 140.46 (Cq), 142.51 (CH), 158.74 (Cq), 158.74 (Cq), 162.41 (Cq); anal. RP-HPLC: *t*_R_ 1.37 min (96.2%, A), 4.24 min (96.8%, B); HRMS (ESI^+^): calcd for C_18_H_14_N_3_O_2_ [M+H]^+^ 304.1086, found 304.0836.

**8-Methyl-10-phenyl-2*H*,3*H*,4*H*,10*H*-pyrimido[4,5-*b*]quinol-ine-2,4-dione (82)**

Prepared using general method 5.7 from **3a** (R^5–7^ = H) and **4** (R^1,2,4^ = H, R^3^ = Me, X = F). Yellow solid (64.7 mg, 65%). Mp: 299–301 ^o^C; IR (KBr): 3,433 (NH), 1,709 (C=O), 1,672 (C=O), 1,608 (C=C) cm^-1^; ^1^H-NMR: *δ* 2.32 (3H, s, Me), 6.48 (1H, s, C9-H), 7.35 (1H, d, *J* = 8.1 Hz, C7-H), 7.41 (2H, d, *J* = 7.2 Hz, Ph 2-H), 7.59-7.72 (3H, m, Ph-H), 8.12 (1H, d, *J* = 8.1 Hz, C6-H), 9.07 (1H, s, C5-H), 11.03 (1H, s, N3-H); ^13^C-NMR: *δ* 22.66 (CH_3_), 115.06 (Cq), 117.05 (CH), 119.58 (Cq), 126.56 (CH), 128.88 (CH), 129.81 (CH), 130.71 (CH), 131.73 (CH), 138.10 (Cq), 142.45 (Cq), 142.67 (CH), 146.79 (Cq), 156.91 (Cq), 159,19 (Cq), 162.49 (Cq); anal. RP-HPLC: *t*_R_ 1.20 min (100%, A), 4.02 min (97.8%, B); HRMS (ESI^+^): calcd for C_18_H_14_N_3_O_2_ [M+H]^+^ 304.1086, found 304.1021.

**9-Methyl-10-phenyl-2*H*,3*H*,4*H*,10*H*-pyrimido[4,5-*b*]quinol-ine-2,4-dione (83)**

Prepared using general method 5.7 from **3a** (R^5–7^ = H) and **4** (R^1–3^ = H, R^4^ = Me, X = F). Yellow solid (37.8 mg, 38%). Mp: 337–338 ^o^C (dec); IR (KBr): 3,441 (NH), 1,703 (C=O), 1,665 (C=O), 1,619 (C=C) cm^-1^; ^1^H-NMR: *δ* 1.59 (3H, s, Me), 7.39-7.47 (3H, m, C8-H & Ph 2-H), 7.53-7.61 (4H, m, C7-H & Ph-H), 8.10 (1H, d, *J* = 7.9 Hz, C6-H), 9.07 (1H, s, C5-H), 11.07 (1H, s, N3-H); ^13^C-NMR: *δ* 22.34 (CH_3_), 115.23 (Cq), 123.04 (Cq), 125.08 (CH), 127.13 (Cq), 129.24 (CH), 129.53 (CH), 130.64 (CH), 131.31 (CH), 140.55 (CH), 140.77 (Cq), 140.95 (Cq), 144.01 (CH), 156.84 (Cq), 160.62 (Cq), 162.35 (Cq); anal. RP-HPLC: *t*_R_ 1.28 min (97.2%, A), 14.40 min (97.9%, B); HRMS (ESI^+^): calcd for C_18_H_14_N_3_O_2_ [M+H]^+^ 304.1086, found 304.1097.

**10-(4-Chlorophenyl)-6-methyl-2*H*,3*H*,4*H*,10*H*-pyrimido[4,5-*b*]quinol-ine-2,4-dione (84)**

Prepared using general method 5.7 from **3a** (R^5,6^ = H, R^7^ = Cl) and **4** (R^1^ = Me, R^2–4^ = H, X = F). Yellow solid (301.8 mg, 30%). Mp: 339–341 ^o^C (dec); IR (KBr): 3,424 (NH), 1,701 (C=O), 1,674 (C=O), 1,599 (C=C) cm^-1^; ^1^H-NMR: *δ* 2.75 (3H, s, Me), 6.62 (1H, d, *J* = 7.9 Hz, C9-H), 7.37 (1H, d, *J* = 7.9 Hz, C7-H), 7.47 (2H, d, AA’BB’ system, *J* = 8.7 Hz, Ph 2-H), 7.62 (1H, t, *J* = 7.9 Hz, C8-H), 7.76 (2H, d, AA’BB’ system, *J* = 8.7 Hz, Ph 3-H), 9.02 (1H, s, C5-H), 11.11 (1H, s, N3-H); ^13^C-NMR: *δ* 19.01 (CH_3_), 115.30 (Cq), 115.93 (CH), 120.33 (Cq), 126.15 (CH), 130.79 (CH), 130.93 (CH), 134.47 (Cq), 135.45 (CH), 137.23 (Cq), 139.01 (CH), 139.68 (Cq), 142.68 (Cq), 156.79 (Cq), 158.82 (Cq), 162.46 (Cq); anal. RP-HPLC: *t*_R_ 2.00 min (97.2%, A), 5.37 min (98.2%, B); HRMS (ESI^+^): calcd for C_18_H_13_ClN_3_O_2_ [M+H]^+^ 338.0697, found 338.0702.

**10-(4-Chlorophenyl)-7-methyl-2*H*,3*H*,4*H*,10*H*-pyrimido[4,5-*b*]quinol-ine-2,4-dione (85)**

Prepared using general method 5.7 from **3a** (R^5,6^ = H, R^7^ = Cl) and **4** (R^1,3,4^ = H, R^2^ = Me, X = F). Yellow solid (47.6 mg, 48%). Mp: > 350 ^o^C; IR (KBr): 3,434 (NH), 1,701 (C=O), 1,668 (C=O), 1,608 (C=C) cm^-1^; ^1^H-NMR: *δ* 2.49 (3H, s, Me), 6.70 (1H, d, *J* = 8.8 Hz, C9-H), 7.47 (2H, d, AA’BB’ system, *J* = 8.6 Hz, Ph 2-H), 7.58 (1H, dd, ^4^*J* = 1.9 Hz, ^3^*J* = 8.8 Hz, C8-H), 7.76 (2H, d, AA’BB’ system, *J* = 8.6 Hz, Ph 3-H), 8.01 (1H, s, C6-H), 9.03 (1H, s, C5-H), 11.07 (1H, s, N3-H); ^13^C-NMR: *δ* 20.68 (CH_3_), 116.01 (Cq), 117.48 (CH), 121.44 (Cq), 130.23 (Cq), 130.76 (CH), 130.93 (CH), 134.46 (Cq), 134.49 (Cq), 137.02 (CH), 137.04 (CH), 140.28 (Cq), 142.61 (CH), 156.74 (Cq), 158.85 (Cq), 162.36 (Cq); anal. RP-HPLC: *t*_R_ 2.50 min (97.4%, A), 5.74 min (99.1%, B); HRMS (ESI^+^): calcd for C_18_H_13_ClN_3_O_2_ [M+H]^+^ 338.0697, found 338.0670.

**10-(4-Chlorophenyl)-9-methyl-2*H*,3*H*,4*H*,10*H*-pyrimido[4,5-*b*]quinol-ine-2,4-dione (86)**

Prepared using general method 5.7 from **3a** (R^5,6^ = H, R^7^ = Cl) and **4** (R^1–3^ = H, R^4^ = Me, X = F). Yellow solid (26.4 mg, 26%). Mp: > 350 ^o^C; IR (KBr): 3,428 (NH), 1,715 (C=O), 1,656 (C=O), 1,617 (C=C) cm^-1^; ^1^H-NMR: *δ* 1.67 (3H, s, Me), 7.42 (1H, t, *J* = 7.6 Hz, C7-H), 7.51 (2H, d, AA’BB’ system, *J* = 8.6 Hz, Ph 2-H), 7.59-7.66 (3H, m, C8-H & Ph 3-H), 8.11 (1H, d, *J* = 7.6 Hz, C6-H), 9.07 (1H, s, C5-H), 11.09 (1H, s, N3-H); ^13^C-NMR: *δ* 22.81 (CH_3_), 115.25 (Cq), 123.04 (Cq), 125.18 (CH), 126.82 (Cq), 129.29 (CH), 131.38 (CH), 132.52 (CH), 134.11 (Cq), 139.81 (Cq), 140.56 (Cq), 140.60 (CH), 144.15 (CH), 156.75 (Cq), 160.68 (Cq), 162.26 (Cq); anal. RP-HPLC: *t*_R_ 2.12 min (100%, A), 2.70 min (98.4%, B); HRMS (ESI^+^): calcd for C_18_H_13_ClN_3_O_2_ [M+H]^+^ 338.0697, found 338.0664.

**10-(2-Fluorophenyl)-6-methyl-2*H*,3*H*,4*H*,10*H*-pyrimido[4,5-*b*]quinol-ine-2,4-dione (87)**

Prepared using general method 5.7 from **3a** (R^5^ = F, R^6,7^ = H) and **4** (R^1^ = Me, R^2–4^ = H, X = F). Yellow solid (129.6 mg, 16%). Mp: 309–310 ^o^C; IR (KBr): 3,467 (NH), 1,702 (C=O), 1,653 (C=O), 1,597 (C=C) cm^-1^; ^1^H-NMR: 2.76 (3H, s, Me), 6.66 (1H, d, *J* = 8.0 Hz, C9-H), 7.40 (1H, d, *J* = 8.0 Hz, C7-H), 7.49-7.76 (5H, m, C8-H & Ph-H), 9.05 (1H, s, C5-H), 11.19 (1H, s, N3-H); ^13^C-NMR: *δ* 19.04 (CH_3_), 115.01 (CH), 115.06 (Cq), 117.66 (CH, d, *J* = 19.01 Hz), 120.31 (Cq), 125.26 (Cq, d, *J* = 13.60 Hz), 126.11 (CH, d, *J* = 3.51 Hz), 126.53 (CH), 130.98 (CH), 132.54 (CH, d, *J* = 8.10 Hz), 135.95 (CH), 139.63 (CH), 140.17 (Cq), 142.02 (Cq), 156.81 (Cq), 157.43 (Cq, d, *J* = 247.76 Hz), 158.55 (Cq), 162.24 (Cq); anal. RP-HPLC: *t*_R_ 1.34 min (100%, A), 4.28 min (99.0%, B); HRMS (ESI^+^): calcd for C_18_H_13_FN_3_O_2_ [M+H]^+^ 322.0992, found 322.0978;

**10-(2-Fluorophenyl)-7-methyl-2*H*,3*H*,4*H*,10*H*-pyrimido[4,5-*b*]quinol-ine-2,4-dione (88)**

Prepared using general method 5.7 from **3a** (R^5^ = F, R^6,7^ = H) and **4** (R^1,3,4^ = H, R^2^ = Me, X = F). Yellow solid (67.9 mg, 68%). Mp: 317–319 ^o^C; IR (KBr): 3,423 (NH), 1,705 (C=O), 1,657 (C=O), 1,609 (C=C) cm^-1^; ^1^H-NMR: *δ* 2.42 (3H, s, Me), 6.74 (1H, d, *J* = 8.7 Hz, C9-H), 7.49-7.65 (4H, m, C8-H & Ph-H), 7.69-7.76 (1H, m, Ph-H), 8.04 (1H, s, C6-H), 9.07 (1H, s, C5-H), 11.14 (1H, s, N3-H); ^13^C-NMR: *δ* 20.63 (CH_3_), 115.86 (Cq), 116.65 (CH), 117.65 (CH, d, *J* = 18.79 Hz), 121.42 (Cq), 125.06 (Cq, d, *J* = 13.71 Hz), 126.56 (CH, d, *J* = 3.05 Hz), 130.98 (CH), 131.26 (CH), 132.50 (CH, d, *J* = 6.38 Hz), 134.95 (Cq), 137.50 (CH), 139.62 (Cq), 143.12 (CH), 156.77 (Cq), 157.61 (Cq, d, *J* = 248.01 Hz), 158.59 (Cq), 162.17 (Cq); anal. RP-HPLC: *t*_R_ 1.53 min (98.1%, A), 4.49 min (100%, B); HRMS (ESI^+^): calcd for C_18_H_13_FN_3_O_2_ [M+H]^+^ 322.0992, found 322.0960.

**10-(2-Fluorophenyl)-8-methyl-2*H*,3*H*,4*H*,10*H*-pyrimido[4,5-*b*]quinol-ine-2,4-dione (89)**

Prepared using general method 5.7 from **3a** (R^5^ = F, R^6,7^ = H) and **4** (R^1,2,4^ = H, R^3^ = Me, X = F). Yellow solid (16.3 mg, 16%). Mp: > 350 ^o^C; IR (KBr): 3,442 (NH), 1,707 (C=O), 1,673 (C=O), 1,608 (C=C) cm^-1^; ^1^H-NMR: *δ* 2.37 (3H, s, Me), 6.60 (1H, s, C9-H), 7.39 (1H, d, *J* = 8.0 Hz, C7-H), 7,49-7.65 (3H, m, Ph-H), 7.69-7.77 (1H, m, Ph-H), 8.15 (1H, d, *J* = 8.0 Hz, C6-H), 9.10 (1H, s, C5-H), 11.10 (1H, s, N3-H); ^13^C-NMR (CDCl_3_): *δ* 114.17 (CH, d, *J* = 4.32 Hz), 118.07 (CH, d, *J* = 19.46 Hz), 119.12 (Cq), 122.01 (CH, d, *J* = 4.32 Hz), 123.48 (Cq), 124.73 (Cq, d, *J* = 12.96 Hz), 127.18 (CH, d, *J* = 4.62 Hz), 130.38 (CH), 133.26 (CH, d, *J* = 8.89 Hz), 134.23 (CH), 134.67 (Cq), 140.81 (Cq), 143.37 (CH), 156.84 (Cq), 158.78 (Cq, d, *J* = 263.14 Hz), 159.64 (Cq), 162.24 (Cq); anal. RP-HPLC: *t*_R_ 1.34 min (100%, A), 5.09 min (98.8%, B); HRMS (ESI^+^): calcd for C_18_H_13_FN_3_O_2_ [M+H]^+^ 322.0992, found 322.0995.

**10-(2-Fluorophenyl)-9-methyl-2*H*,3*H*,4*H*,10*H*-pyrimido[4,5-*b*]quinol-ine-2,4-dione (90)**

Prepared using general method 5.7 from **3a** (R^5^ = F, R^6,7^ = H) and **4** (R^1–3^ = H, R^4^ = Me, X = F). Yellow solid (53.1 mg, 53%). Mp: 287–290 ^o^C; IR (KBr): 3,442 (NH), 1,712 (C=O), 1,657 (C=O), 1,619 (C=C) cm^-1^; ^1^H-NMR: *δ* 1.68 (3H, s, Me), 7.36-7.54 (4H, m, C8-H & Ph-H), 7.62-7.70 (2H, m, C7-H & Ph-H), 8.13 (1H, d, *J* = 7.4 Hz, C6-H), 9.10 (1H, s, C5-H), 11.16 (1H, s, N3-H); ^13^C-NMR: *δ* 21.37 (CH_3_), 115.11 (Cq), 116.63 (CH, d, *J* = 19.5 6Hz), 123.03 (Cq), 125.40 (Cq), 125.50 (CH, d, *J* = 3.19 Hz), 126.28 (Cq), 128.52 (Cq, d, *J* = 13.26 Hz) 131.63 (CH), 131.84 (CH), 132.38 (CH, d, *J* = 8.08 Hz), 140.35 (Cq), 140.74 (CH), 144.51 (Cq), 156.81 (Cq), 158.84 (Cq, d, *J* = 249.14 Hz), 160.25 (Cq), 162.16 (Cq); anal. RP-HPLC: *t*_R_ 1.40 min (97.4%, A), 15.64 min (96.8% B); HRMS (ESI^+^): calcd for C_18_H_13_FN_3_O_2_ [M+H]^+^ 322.0992, found 322.0959.

**9-Hydroxy-10-phenyl-2*H*,3*H*,4*H*,10*H*-pyrimido[4,5-*b*]quinol-ine-2,4-dione (91)**

Prepared using general method 5.7 from **3a** (R^5–7^ = H) and **4** (R^1–3^ = H, R^4^ = OH, X = F). Yellow solid (44.6 mg, 45%). Mp: 207–208 ^o^C; IR (KBr): 3,475 (NH), 1,695 (C=O), 1,650 (C=O), 1,602 (C=C) cm^-1^; ^1^H-NMR: *δ* 7.09 (1H, dd, ^4^*J* = 1.2 Hz, ^3^*J* = 7.8 Hz, C8-H), 7.28-7.34 (3H, m), 7.41-7.48 (3H, m), 7.65 (1H, dd, ^4^*J* = 1.2 Hz, ^3^*J* = 7.8 Hz, C6-H), 9.00 (1H, s, C5-H), 10.00 (1H, s, OH), 10.98 (1H, s, N3-H); ^13^C-NMR: *δ* 115.68 (Cq), 121.79 (CH), 122.63 (CH), 123.67 (Cq), 125.70 (CH), 128.06 (CH), 128.35 (CH), 128.70 (CH), 130.24 (Cq), 142.38 (Cq), 143.58 (Cq), 147.07 (Cq), 156.78 (Cq), 159.96 (Cq), 162.42 (Cq); anal. HPLC: *t*_R_ 0.63 min (100%, A), 6.18 min (97.4%, B); HRMS (ESI^+^): calcd for C_17_H_12_N_3_O_3_ [M+H]^+^ 306.0878, found 306.0844.

**10-(4-Chlorophenyl)-9-hydroxy-2H,3H,4H,10H-pyrimi-do[4,5-b]quinoline-2,4-dione (92)**

Prepared using general method 5.7 from **3a** (R^5,6^ = H, R^7^ = Cl; 136 mg, 0.57 mmol)[^2^](#_ENREF_2) and **4** (R^1–3^ = H, R^4^ = OH, X = Cl; 107 mg, 0.68 mmol). Yellow solid (17.5 mg, 9%). Mp 316–318 ^o^C; IR (KBr): 3,434 (NH), 1,702 (C=O), 1,655 (C=O), 1,603 (C=C) cm^-1^; ^1^H-NMR: *δ* 7.08 (1H, d, *J* = 7.6 Hz, C8-H), 7.31 (1H, t, *J* = 7.6 Hz, C7-H), 7.37 (2H, d, *J* = 8.2 Hz, Ph 2-H), 7.50 (2H, d, *J* = 8.2 Hz, Ph 3-H), 7.76 (1H, d, *J* = 7.6 Hz, C6-H), 8.99 (1H, s, C5-H), 10.16 (1H, s, OH), 11.00 (1H, s, N3-H); ^13^C-NMR: *δ* 115.68 (Cq), 121.84 (CH), 122.70 (CH), 123.69 (Cq), 125.79 (CH), 128.34 (CH), 130.05 (Cq), 130.64 (CH), 132.74 (Cq), 141.26 (Cq), 143.71 (CH), 146.95 (Cq), 156.70 (Cq), 159.95 (Cq), 162.34 (Cq); anal. RP-HPLC: *t*_R_ 0.80 min (99.8%; A), 10.85 min (100%; B); HRMS (ESI^+^): calcd for C_17_H_11_ClN_3_O_3_ [M+H]^+^ 340.0489, found 340.0513.

**9-Fluoro-10-phenyl-2*H*,3*H*,4*H*,10*H*-pyrimido[4,5-*b*]quinol-ine-2,4-dione (93)**

Prepared using general method 5.7 from **3a** (R^5–7^ = H) and **4** (R^1–3^ = H, R^4^ = X = F). Yellow solid (29.3 mg, 29%). Mp: 260–262 ^o^C (dec); IR (KBr): 3,410 (NH), 1,703 (C=O), 1,667 (C=O), 1,613 (C=C) cm^-1^; ^1^H-NMR: *δ* 7.43-7.57 (6H, m, C8-H & Ph-H), 7.60 (1H, ddd, ^4^*J* = 1.5 Hz, ^3^*J* = 6.9 Hz, C7-H), 8.07 (1H, d, *J* = 6.9 Hz, C6-H), 9.12 (1H, s, C5-H), 11.14 (1H, s, N3-H); ^13^C-NMR: *δ* 116.78 (Cq), 122.61 (CH, d, *J* = 21.93 Hz), 123.84 (Cq), 125.42 (CH, d, *J* = 8.01 Hz), 128.22 (CH, d, *J* = 4.70 Hz), 128.41 (CH, d, *J* = 3.77 Hz), 129.14 (CH), 129.41 (CH), 130.32 (Cq, d, *J* = 3.79 Hz), 140.98 (Cq, d, *J* = 3.88 Hz), 142.83 (CH, d, *J* = 2.21 Hz), 150.08 (Cq, d, *J* = 250.69 Hz), 156.69 (Cq), 159.98 (Cq), 162.10 (Cq); anal. RP-HPLC: *t*_R_ 1.02 min (99.5%, A), 9.57 min (97.6%, B); HRMS (ESI^+^): calcd for C_17_H_11_FN_3_O_2_ [M+H]^+^ 308.0835, found 308.0813.

**10-(4-Chlorophenyl)-9-fluoro-2*H*,3*H*,4*H*,10*H*-pyrimido[4,5-*b*]quinoline-2,4-dione (94)**

Prepared using general method 5.7 from **3a** (R^5,6^ = H, R^7^ = Cl) and **4** (R^1–3^ = H, R^4^ = X = F). Yellow solid (20.6 mg, 21%). Mp: 268–269 ^o^C (dec); IR (KBr): 3,431 (NH), 1,716 (C=O), 1,657 (C=O), 1,614 (C=C) cm^-1^; ^1^H-NMR: *δ* 7.46-7.52 (3H, m, C7-H & Ph 2-H), 7.59-7.66 (3H, m, C8 & Ph 3-H), 8.07 (1H, d, *J* = 7.8 Hz, C6-H), 9.12 (1H, s, C5-H), 11.16 (1H, s, N3-H); ^13^C-NMR: *δ* 116.76 (Cq), 122.65 (CH, d, *J* = 21.88 Hz), 123.84 (Cq), 125.49 (CH, d, *J* = 8.02 Hz), 128.48 (CH, d, *J* = 3.47 Hz), 129.50 (CH), 130.15 (Cq, d, *J* = 4.53 Hz), 130.25 (CH, d, *J* = 4.47 Hz), 133.82 (Cq), 139.84 (Cq, d, *J* = 3.79 Hz), 142.97 (CH), 150.02 (Cq, d, *J* = 251.49 Hz), 156.64 (Cq), 160.05 (Cq), 162.08 (Cq); anal. RP-HPLC: *t*_R_ 1.82 min (99.8%, A), 19.97 min (98.2%, B); HRMS (ESI^+^): calcd for C_17_H_10_ClFN_3_O_2_ [M+H]^+^ 342.0445, found 342.0435.

- 1. **10-(3-Chlorophenyl)-2,4-dioxo-2H,3H,4H,10H-pyrimido [4,5-b]quinoline-9-carbonitrile (95)**

Prepared using general method 5.7 from **3a** (R^5,7^ = H, R^6^ = Cl; 102 mg, 0.43 mmol) and **4** (R^1–3^ = H, R^4^ = CN, X = OTs; 156 mg, 0.52 mmol). Yellow solid (96.0 mg, 64%). Mp > 350 ^o^C; IR (KBr): 3,420 (NH), 2,213 (CN), 1,703 (C=O), 1,664 (C=O), 1,619 (C=C) cm^-1^; ^1^H-NMR: *δ* 7.53 (1H, d, *J* = 8.1 Hz, Ph 6-H), 7.61-7.66 (2H, m), 7.72-7.76 (2H, m), 8.23 (1H, dd, ^4^*J* = 1.5 Hz, ^3^*J* = 7.8 Hz, C8-H), 8.55 (1H, dd, ^4^*J* = 1.5 Hz, ^3^*J* = 7.8 Hz, C6-H), 9.16 (1H, s, C5-H), 11.28 (1H, s, N3-H); ^13^C-NMR: *δ* 101.06 (Cq), 114.89 (Cq), 117.10 (Cq), 122.87 (Cq), 124.90 (CH), 129.74 (CH), 130.76 (CH), 131.44 (CH), 131.78 (CH), 134.32 (Cq), 137.80 (CH), 139.07 (Cq), 141.37 (Cq), 142.98 (CH), 143.66 (CH), 156.55 (Cq), 160.12 (Cq), 161.90 (Cq); anal. RP-HPLC: *t*_R_ 0.90 min (99.5%; A), 6.78 min (98.7%; B); HRMS (ESI^+^): calcd for C_18_H_10_ClN_4_O_2_ [M+H]^+^ 349.0492, found 349.0627.

**10-(4-Chlorophenyl)-2,4-dioxo-2*H*,3*H*,4*H*,10*H*-pyrimido[4,5-*b*]quinoline-9-carbonitrile (96)**

Prepared using general method 5.7 from **3a** (R^5,6^ = H, R^7^ = Cl) and **4** (R^1–3^ = H, R^4^ = CN, X = OTs). Yellow solid (37.2 mg, 25%). Mp: > 350 ^o^C; IR (KBr): 3,416 (NH), 2,198 (CN), 1,704 (C=O), 1,666 (C=O), 1,621 (C=C) cm^-1^; ^1^H-NMR: *δ* 7.58 (2H, d, AA’BB’ system, *J* = 8.7 Hz, Ph 2-H), 7.62 (1H, t, *J* = 7.7 Hz, C7-H), 7.68 (2H, d, AA’BB’ system, *J* = 8.7 Hz, Ph 3-H), 8.23 (1H, dd, ^4^*J* = 1.5 Hz, ^3^*J* = 7.7 Hz, C8-H), 8.54 (1H, dd, ^4^*J* = 1.5 Hz, ^3^*J* = 7.7 Hz, C6-H), 9.15 (1H, s, C5-H), 11.26 (1H, s, N3-H); ^13^C-NMR: *δ* 101.15 (Cq), 114.87 (Cq), 117.12 (Cq), 122.88 (Cq), 124.85 (CH), 130.23 (CH), 132.75 (CH), 135.99 (Cq), 136.81 (Cq), 137.69 (CH), 141.47 (Cq), 142.95 (CH), 143.56 (CH), 156.56 (Cq), 160.23 (Cq), 161.88 (Cq); anal. RP-HPLC: *t*_R_ 0.92 min (100%, A), 4.92 min (100%, B). HRMS (ESI^+^): calcd for C_18_H_10_ClN_4_O_2_ [M+H]^+^ 349.0492, found 349.0627.

References and notes

1. Wilson, J. M.; Henderson, G.; Black, F.; Sutherland, A.; Ludwig, R. L.; Vousden, K. H.; Robins, D. J. *Bioorg. Med. Chem.* **2007,** *15*, 77.

2. Nagamatsu, T.; Hashiguchi, Y.; Yoneda, F. *J. Chem. Soc. Perkin Trans. 1* **1984**, 561.

3. Ali, H. I.; Ashida, N.; Nagamatsu, T. *Bioorg. Med. Chem.* **2007,** *15*, 6336.

4. Yoneda, F.; Tsukuda, K.; Shinozuka, K.; Hirayama, F.; Uekama, K.; Koshiro, A. *Chem. Pharm. Bull.* **1980,** *28*, 3049.

1. ^*^ Corresponding author. Tel.: +44-115-846-6242; fax: +44-115-951-3412; e-mail: peter.fischer@nottingham.ac.uk [↑](#footnote-ref-2)
